# Clinical and Pharmacogenetic Factors Associated with Response to JAK Inhibitors in Patients with Rheumatoid Arthritis: A Real-World Study of *JAK1*, *JAK2*, and *JAK3* Gene Variants

**DOI:** 10.3390/pharmaceutics18070846

**Published:** 2026-07-11

**Authors:** Alicia Martín Roldán, Noelia Márquez Pete, María del Mar Sánchez Suárez, Susana Rojo Tolosa, Alberto Jiménez Morales

**Affiliations:** 1 Pharmacy Service, Pharmacogenetics Unit, University Hospital Virgen de las Nieves, Avda. de las Fuerzas Armadas 2, 18014 Granada, Spain; alicia.martin.roldan.sspa@juntadeandalucia.es (A.M.R.); alberto.jimenez.morales.sspa@juntadeandalucia.es (A.J.M.); 2 Pharmacy Service, Hospital Comarcal de Baza, Carretera de Murcia S/N, 18800 Granada, Spain; 3 Respiratory Medicine Department, Hospital Universitario Virgen de las Nieves, 18014 Granada, Spain; 4 Ibs. GRANADA, Instituto de Investigación Biosanitaria, 18012 Granada, Spain

**Keywords:** rheumatoid arthritis, janus kinase inhibitors, pharmacogenetics, genetic polymorphism, precision medicine

## Abstract

**Background**: Janus kinase inhibitors (JAK inhibitors) have expanded therapeutic options for rheumatoid arthritis (RA), although factors associated with treatment response in routine clinical practice remain incompletely defined. **Objectives**: This study aimed to evaluate clinical, treatment-related and pharmacogenetic factors associated with response to different JAK inhibitors in patients with RA. **Methods**: An ambispective observational real-world cohort study was conducted in patients with RA treated with tofacitinib, baricitinib, filgotinib, or upadacitinib. Disease activity was assessed at 3 and 6 months using the Disease Activity Score in 28 joints based on C-reactive protein (DAS28-CRP). Clinical response was evaluated according to European Alliance of Associations for Rheumatology (EULAR) response criteria, low disease activity (LDA), and remission thresholds. Clinical, laboratory, and treatment-related variables were collected, and selected single-nucleotide polymorphisms (SNPs) in *JAK1*, *JAK2*, and *JAK3* genes were genotyped. Bivariate and multivariable analyses were performed to identify variables associated with treatment outcomes. **Results**: Lower baseline inflammatory burden and lower disease activity were consistently associated with higher probabilities of EULAR response, LDA, and remission across JAK inhibitors. Treatment-related factors were also associated with improved outcomes. Pharmacogenetic associations were heterogeneous and drug-specific, with the most recurrent exploratory signals involving *JAK2* variants. However, these genetic findings showed variability across outcomes and time points. **Conclusions**: In this real-world RA cohort, clinical and treatment-related factors were the most consistent variables associated with response to JAK inhibitors. Pharmacogenetic variation within the JAK pathway, particularly involving *JAK2*, may contribute to drug-specific variability in response, but these findings should be considered exploratory because of the limited sample size, multiple comparisons, and sparse genotype subgroups. Larger independent studies are required before JAK genotyping can be incorporated into individualized treatment strategies.

## 1. Introduction

RA is a multifactorial autoimmune chronic disease in which the joints become inflamed, causing pain, deformity, and difficulty in movement, although it can also affect other parts of the body. Nowadays, there is a complex and incompletely understood pathogenesis in which genetic susceptibility plays a central role. First-degree relatives of affected individuals have a substantially increased risk of developing RA. Genome-wide association studies have further expanded the number of genetic loci associated with RA, highlighting the polygenic nature of the disease. In addition to genetic factors, epigenetic mechanisms and environmental exposures contribute to promoting a loss of immune tolerance, leading to autoantibody production and chronic inflammation, although the precise gene–environment interactions remain unclear [1].

Clinical remission is the primary therapeutic goal in the management of RA in routine practice. International guidelines broadly agree on treatment strategies involving disease-modifying antirheumatic drugs (DMARDs), which include conventional synthetic (csDMARDs), biologic (bDMARDs), and targeted synthetic agents (tsDMARDs). Guidelines emphasize the consideration of DMARD dose reduction once sustained remission has been achieved [2]. Although effective disease control is crucial to prevent long-term damage and systemic complications, many patients do not respond adequately to csDMARDs or bDMARDs, including methotrexate (MTX). Janus kinase inhibitors (JAK inhibitors) offer a validated alternative for patients who fail to achieve remission with these therapies [3].

Due to the central role of the JAK/STAT pathway in disease pathogenesis, several JAK inhibitors have been approved for treating immune-mediated disorders and hematological malignancies, aiming to counteract JAK/STAT hyperactivation [4]. For RA, the approved drugs include baricitinib, filgotinib, tofacitinib, and upadacitinib [5]. Despite belonging to the same drug class, these agents show distinct efficacy and safety profiles, likely linked to differences in their selectivity for specific JAK isoforms [6].

The JAK family, together with the STAT pathway, governs cellular responses to cytokines, interferons, and growth factors by transmitting intracellular signals to the nucleus and initiating gene expression. JAKs are intracellular nonreceptor protein tyrosine kinases and comprise four members (*JAK1*, *JAK2*, *JAK3*, and *TYK2*). In vitro, studies of *STAT* phosphorylation inhibition demonstrate a preferential targeting of *JAK1*-dependent pathways. However, inhibition of *JAK2* and *JAK3* pathways can also contribute to clinical responses. *JAK2* blockade may enhance the efficacy of non-selective JAK inhibitors through platelet modulation relevant to synovitis, while *JAK3* inhibition impacts lymphocyte proliferation by disrupting IL-7 and IL-15 signaling, potentially improving outcomes [7].

Over 200 variants in *JAK* and *STAT* genes are implicated in human disease. Germline mutations in *JAK1*, *JAK3*, and *TYK2*, as well as in *STAT1*–*STAT5*, drive immune dysregulation, while somatic mutations in *JAK2*, *TYK2*, *STAT3*, *STAT5*, and *STAT6* are associated with malignancies [8]. Numerous pharmacogenetic studies have examined associations between SNPs and response to biologic therapies (BTs) in RA. Candidate genes have primarily been selected from pathways involved in RA-related cytokine signaling. Several studies suggest that specific SNPs may be associated with treatment response and could serve as potential predictors, particularly for *TNF-α* inhibitors, anti-IL-6 receptor agents, and CD20 inhibitors. However, these associations have rarely been replicated in large patient cohorts or confirmed through meta-analyses, underscoring their limited robustness. In addition, few studies have explored the relationship between SNPs and clinical outcomes in RA patients treated with anti-CD80/CD86 agents, anti-interleukin-1 receptor therapies or JAK inhibitors [9].

The goal of this study is to analyze genetic, clinical, and treatment-related factors that may influence the response to JAK inhibitors in patients with RA. Specifically, the study seeks to identify selected SNPs in RA patients treated with upadacitinib, baricitinib, tofacitinib, or filgotinib; assess treatment effectiveness using the European Alliance of Associations for Rheumatology (EULAR) response criteria at 3 and 6 months through biochemical markers and physical measures; and evaluate the impact of demographic, clinical, and therapeutic variables.

## 2. Materials and Methods

### 2.1. Study Design

An ambispective observational cohort study was conducted.

### 2.2. Study Population

The present study included 115 patients over the age of 18 years diagnosed with RA in the Rheumatology Department of the HUVN, according to the American College of Rheumatology (ACR) classification criteria who were treated or had been treated with JAK inhibitors (tofacitinib, upadacitinib, baricitinib and filgotinib) at the Hospital Universitario Virgen de las Nieves (HUVN) in Granada, Spain since the introduction of these drugs at the hospital through March 2025.

Within this cohort, a total of 150 distinct treatment exposures were identified. Patients could receive multiple consecutive therapies over time, each of which was analyzed as an independent treatment course. Specifically, response to treatment was evaluated in 50 exposures for tofacitinib, 44 for baricitinib, 36 for upadacitinib, and 20 for filgotinib. All evaluations were conducted at baseline and at 3 and 6 months post-initiation. Patients with unavailable or incomplete medical records for follow-up, those under the age of 18 and those who did not sign the consent form or withdrew their consent during the study were excluded [10].

### 2.3. Ethics Statements

The study was carried out with the approval of the Ethics and Research Committee of the HUVN (code: SICEIA-2024-001467) in accordance with the Declaration of Helsinki. The subjects who participated in the study signed informed consent for the collection and genetic analysis of saliva samples and for their donation to the Andalusian Public Health System Biobank. The samples were identified by alphanumeric codes.

### 2.4. Sociodemographic and Clinical Variables

Sociodemographic, treatment-related, and clinical data were obtained from patient clinical records. The sociodemographic variables included sex, smoking status, age at RA diagnosis, disease duration, age at JAK inhibitor treatment initiation, treatment duration, biologic-naïve status, number and duration of previous BTs, dose modifications, treatment discontinuation and its reasons, adverse events (AE) to JAK inhibitors and concomitant treatment with glucocorticoids (GCs), statins, vitamin D or csDMARDs (methotrexate [MTX], hydroxychloroquine [HXQ], leflunomide [LFN] or sulfasalazine).

Clinical variables included disease activity score in 28 joints measures (DAS28) [11], C-reactive protein (CRP), erythrocyte sedimentation rate (ESR), Tender Joint Count (TJC), Swollen Joint Count (SJC), patient visual analogue scale (PVAS), physician visual analogue scale (MVAS), serological markers (rheumatoid factor (RF), anti-citrullinated protein antibodies (ACPA)), and metabolic parameters (total cholesterol levels (TC), triglyceride levels (TG), low density lipoprotein cholesterol (LDL) levels, body mass index (BMI)), as well as comorbidity burden assessed by the Charlson Comorbidity Index (CCI).

### 2.5. Genetic Variables

#### 2.5.1. DNA Isolation

Following the patients’ inclusion and signing of the informed consent, saliva samples were collected with buccal swabs (OCR-100, DNA Genotek, Ottawa, QC, Canada). The DNA was extracted using the QlAamp DNA Mini Kit (Qiagen GmbH, Hilden, Germany), following the manufacturer’s instructions for purifying DNA from saliva, and stored at −40 °C. The DNA concentration and purity were measured using a NanoDrop 2000 UV spectrophotometer (NanoDrop Technologies Inc., Wilmington, DE, USA)with the absorbance ratios at 280/260 and 280/230.

#### 2.5.2. Detection of Gene Polymorphisms

A total of 13 SNPs were analyzed, including *JAK1* (rs2230587, rs2230588, rs2780815, rs10889504, rs310241), *JAK2* (rs10119004, rs7857730, rs2274472, rs2230722, rs2230724) and *JAK3* (rs3212780, rs3212752, rs3008).

The analyzed SNPs were selected using a literature-based candidate-gene approach focused on *JAK1*, *JAK2*, and *JAK3* because of their central involvement in JAK/STAT signaling and their relevance to the mechanism of action of JAK inhibitors. Variants were prioritized according to previous associations with RA or other immune-mediated diseases, their genomic location and potential functional or regulatory consequence, their allele frequency and the availability of validated commercial TaqMan assays (Supplementary Materials Table S1).

Genotyping was performed by real-time polymerase chain reaction (PCR) using TaqMan^®^ probes (Applied Biosystems, Waltham, MA, USA) according to the manufacturer’s instructions. PCR amplification was carried out using the QuantStudio 3 Real-Time PCR System (96-well format). Genotype calling and analysis were performed using QuantStudio Design and Analysis Software v1.5.2 and QuantStudio 12K Flex Software v1.4.

The assay IDs used were as follows: *JAK1* (rs2230587 (C___1766240_20), rs2230588 (C__22273175_10), rs2780815 (C___1766225_20), rs10889504 (C___1766309_10), rs310241 (C___1767439_10)), *JAK2* (rs10119004 (C__30016880_10), rs7857730 (C__29340600_20), rs2274472 (C__16181933_10), rs2230722 (C__30502833_10), rs2230724 (C__22273141_10)) and *JAK3* (rs3212780 (C__32396501_10), rs3212752 (C__32396521_10), rs3008 (C___2677324_10)).

### 2.6. Response Variables

Clinical response was evaluated at 3 and 6 months according to EULAR response criteria, LDA, and remission. EULAR response was classified as satisfactory, comprising good or moderate response, or unsatisfactory, corresponding to no response, according to the change in DAS28-CRP from baseline and the DAS28-CRP value at follow-up. LDA was defined as 2.6 ≤ DAS28-CRP ≤ 3.2, and remission as DAS28-CRP < 2.6. DAS28-CRP was calculated according to standard methodology [12,13].

### 2.7. Statistical Analysis

The descriptive analysis was performed using R4.5.3 software. The quantitative variables were expressed as the mean (±standard deviation) for those that complied with normality and as the median and percentiles (p25–p75) for the variables that did not follow a normal distribution. Normality was assessed using the Shapiro–Wilk test *(n* ≤ 50) and the Kolmogorov–Smirnov test (*n* > 50). The bivariate analysis between the response and the genetic variables was performed using Pearson’s chi-squared test or applying Fisher’s exact test for the qualitative variables. For the quantitative variables, Student’s *t*-test was applied to the variables that complied with normality. The Mann–Whitney *U* test was applied for non-normal variables. Variables with *p* < 0.05 in bivariate analyses, together with covariates considered clinically relevant a priori, were considered for inclusion in the multivariable models.

Multivariable logistic regression analysis was used to estimate adjusted odds ratios (ORs) and 95% confidence intervals (95% CIs) for factors associated with EULAR response, LDA, and remission. All effectiveness, bivariate, and multivariable analyses were performed separately for each JAK inhibitor. Within each drug-specific analysis, each patient contributed a single treatment episode and one observation at each analyzed follow-up time point.

The goodness of fit for each model was analyzed with the Hosmer–Lemeshow test and the omnibus test of coefficients, and the Cox–Snell and Nagelkerke *r*^2^ coefficients were also calculated. To account for multiple comparisons, *p*-values from multivariable analyses were adjusted using the Benjamini–Hochberg procedure to control the false discovery rate (FDR). The correction was applied independently within each model, defined by clinical outcome and assessment time point (3 and 6 months). FDR-adjusted *p*-values below 0.05 were considered statistically significant.

Nominal two-sided *p*-values < 0.05 were considered statistically significant in bivariate analyses, whereas FDR-adjusted *p*-values < 0.05 were considered statistically significant in multivariable analyses. Analyses were performed using R 4.5.3 or the PLINK toolset free-access software for whole-genome association analysis.

We determined the Hardy–Weinberg equilibrium and the haplotype frequencies and calculated Lewontin’s D-prime (D′) and the linkage disequilibrium coefficient (*r*^2^). The linkage disequilibrium (LD) for each SNP was calculated with the PLINK genome association analysis program [13]. The haplotype frequencies and their association with the response variable were analyzed using the SNPstats program, a web-based tool for analysis of association studies.

## 3. Results

### 3.1. Sociodemographic and Clinical Variables

A total of 115 patients with RA treated with JAK inhibitors were included. Overall, data on 150 lines of treatment were collected: 50 with tofacitinib, 44 with baricitinib, 36 with upadacitinib, and 20 with filgotinib.

Baseline clinical and sociodemographic characteristics are shown in Table 1. Median age at diagnosis was 43 years (33–51), 81.7% were women, and median disease duration was 12 years (8–20). The median interval between RA diagnosis and JAK inhibitor initiation was 8.7 years (4.3–15.6). The overall duration of JAK inhibitor treatment was 36.1 months (13.4–61.7). At baseline, mean DAS28 was 4.5 ± 1.3, median CRP was 3.9 mg/L (1.2–9.3), and median ESR was 19 mm/h (9–35.5). Only 9.6% of patients were biologic-naïve at JAK inhibitor initiation, indicating that most had received prior advanced therapy. At treatment initiation, median BMI was 27.3 kg/m^2^ (24.3–31.2).

### 3.2. Clinical Effectiveness of JAK Inhibitors

Clinical effectiveness outcomes across the different JAK inhibitors are summarized in Table 2 and Table S2. After 3 months of treatment, satisfactory EULAR response rates ranged from 27.8% for upadacitinib to 40.9% for baricitinib. After 6 months of treatment, numerically higher response rates were observed for tofacitinib and filgotinib. Remission rates generally increased over time across all treatment groups, particularly for upadacitinib and filgotinib.

Overall, treatment effectiveness patterns were broadly comparable among JAK inhibitors, although direct comparisons should be interpreted cautiously due to differences in sample size and treatment history between groups. Notably, all patients treated with filgotinib had previously received advanced therapy, including at least one prior JAK inhibitor, indicating that this subgroup represented a particularly treatment-experienced population.

### 3.3. Genotype Distribution

All polymorphisms evaluated in the cohorts treated with the four JAK inhibitors showed genotype distributions consistent with the Hardy–Weinberg equilibrium (HWE) model. In the groups, all SNPs fulfilled HWE criteria (*p* ≥ 0.05), and the concordance between observed and expected heterozygosity values indicated adequate genotyping quality (Table S3).

Minor allele frequencies (MAF) for all loci were above the 1% inclusion threshold across treatments. Only *JAK3* rs3212752 showed a particularly low MAF value in the filgotinib (MAF = 0.02) and upadacitinib (MAF = 0.05) cohorts, which limited the statistical power of analyses involving this variant (Table S4).

Linkage disequilibrium (LD) analysis revealed a consistent LD structure across the four treatment groups (Table S5). On chromosome 1, a strong LD signal was observed between rs310241 and rs2230588 (D′ ≈ 0.881–1; r^2^ = 0.727–0.953 across cohorts). Moderate LD was also detected between rs2230587 and rs10889504 (D′ ≈ 0.640–1; r^2^ = 0.503–0.690 across cohorts), as well as between rs310241 and rs2780815 and rs2230588 and rs2780815. On chromosome 9, the strongest LD was detected between rs7857730 and rs2230724 (D′ ≈ 1; r^2^ = 0.751–0.926), suggesting the presence of a stable haplotype block in this region. Additional moderate LD signals were observed between rs10119004 and rs7857730, and between rs10119004 and rs2230724, whereas rs2274472 and rs2230724 showed comparatively low LD values. Importantly, the LD patterns were highly similar among JAK inhibitors, indicating that the haplotypic structure of the analyzed loci remained consistent across treatment groups.

Haplotype frequency estimates are presented in Tables S6–S11 for baricitinib, Tables S12–S17 for filgotinib, Tables S18–S23 for tofacitinib and Tables S24–S29 for upadacitinib.

### 3.4. Clinical, Treatment-Related, and Pharmacogenetic Factors Associated with Response

#### 3.4.1. Clinical and Treatment-Related Bivariate Associations

At 3 months in the tofacitinib cohort, a lower baseline inflammatory burden was significantly associated with a satisfactory EULAR response. Specifically, lower baseline TJC (OR = 0.59; 95% CI = 0.38–0.80; *p* < 0.001), PVAS (OR = 0.72; 95% CI = 0.53–0.94; *p* = 0.026), and MVAS (OR = 0.57; 95% CI = 0.38–0.79; *p* = 0.002) were associated with improved response.

Higher baseline DAS28, TJC, PVAS, and MVAS values significantly reduced the probability of achieving low disease activity (LDA) (Table S32). At this time, bivariate analysis of achieving remission showed associations with lower baseline DAS28 (OR = 41.27; 95% CI = 4.36–1788.46; *p* < 0.001), RF negativity (OR = 16.0; 95% CI = 2.06–334.40; *p* = 0.013). Patients who achieved remission had lower baseline TC levels than those who did not achieve remission (165 ± 22.5 vs. 214.5 ± 42.1 mg/dL; *p* = 0.003). Higher baseline TC levels were associated with increased odds of non-remission (OR = 1.05; 95% CI = 1.01–1.10; *p* = 0.003) (Table S34).

At 6 months, lower baseline TJC (OR = 0.41; 95% CI = 0.20–0.68; *p* = 0.003), SJC (OR = 0.20; 95% CI = 0.05–0.55; *p* = 0.009), PVAS (OR = 0.64; 95% CI = 0.43–0.89; *p* = 0.014), and MVAS (OR = 0.47; 95% CI = 0.27–0.71; *p* = 0.002) remained significantly associated with satisfactory EULAR response (Table S30). Achievement of LDA was negatively influenced by higher baseline DAS28 (OR = 2.82; 95% CI = 1.08–10.78; *p* = 0.002), ESR (OR = 1.16; 95% CI = 1.04–1.39; *p* < 0.001) and RF levels (OR = 1.01; 95% CI = 1.00–1.05; *p* = 0.011) (Table S32).

In the baricitinib cohort at 3 months, the absence of concomitant GC was strongly associated with both a satisfactory EULAR response (OR = 9.58; 95% CI = 2.29–51.64; *p* = 0.001) and remission (OR = 15.18; 95% CI = 3.42–84.26; *p* < 0.001) (Tables S34 and S38). Lower baseline TJC was associated with improved LDA rates (OR = 0.55; 95% CI = 0.28–0.85; *p* = 0.001) (Table S38). At 6 months, lower BMI was associated with satisfactory EULAR response (OR = 0.79; 95% CI = 0.60–0.98; *p* = 0.038), while higher BMI reduced the probability of remission (OR = 0.76; 95% CI = 0.56–0.96; *p* = 0.034). The absence of concomitant GC continued to favor satisfactory response (OR = 6.38; 95% CI = 1.51–31.77; *p* = 0.011) and remission (OR = 15.00; 95% CI = 3.08–96.56; *p* < 0.001) (Tables S34 and S38).

In the filgotinib cohort at 3 months, patients who achieved satisfactory EULAR response exhibited significantly lower baseline TJC (OR = 0.28; 95% CI = 0.05–0.65; *p* < 0.001), PVAS (OR = 2.31; 95% CI = 1.25–6.95; *p* = 0.007), and MVAS (OR = 1.76; 95% CI = 1.07–3.45; *p* = 0.027) (Table S42). Lower baseline DAS28, TJC, and SJC were also associated with achieving LDA and remission (Tables S42 and S44). At 6 months, higher baseline DAS28 (*p* < 0.001), TJC (*p* = 0.003), and SJC (*p* = 0.010) were associated with poorer outcomes. Furthermore, lower remission rates were associated with higher baseline MVAS (OR = 6.04; 95% CI = 1.55–131.35; *p* = 0.001), CRP (OR = 2.32; 95% CI = 1.11–9.46; *p* = 0.023), and baseline TG levels (OR = 1.07; 95% CI = 1.01–1.18; *p* = 0.004) (Table S46).

Finally, for the upadacitinib cohort at 3 months, shorter RA duration was significantly associated with a higher probability of achieving a satisfactory EULAR response (OR = 0.91; 95% CI = 0.81–1.00; *p* = 0.029) and LDA achievement (OR = 0.89; 95% CI = 0.69–1.03; *p* = 0.009). Lower CRP (OR = 0.82; 95% CI = 0.53–0.98; *p* = 0.013) and lower RF levels (OR = 0.89; 95% CI = 0.69–0.97; *p* = 0.001) also favored LDA (Tables S46 and S48). At 6 months, the absence of concomitant GC was associated with satisfactory response (OR = 8.40; 95% CI = 1.75–51.54; *p* = 0.007) and remission (OR = 10.0; 95% CI = 1.87–80.79; *p* = 0.007). Lower baseline SJC (OR = 0.78; 95% CI = 0.56–0.99; *p* = 0.042), ESR (OR = 0.94; 95% CI = 0.88–1.01; *p* = 0.040), and TG levels (OR = 0.97; 95% CI = 0.93–0.99; *p* = 0.016) also correlated with improved clinical outcomes (Tables S46 and S50).

Overall, lower baseline disease activity and inflammatory burden were the most recurrent clinical factors associated with favorable outcomes across JAK inhibitors. Absence of concomitant GC use was particularly relevant in the baricitinib and upadacitinib cohorts, whereas BMI and previous treatment exposure showed drug-specific associations.

#### 3.4.2. Pharmacogenetic Bivariate Associations

In the tofacitinib cohort, *JAK2* rs7857730 GG homozygotes showed lower odds of satisfactory EULAR response in the bivariate analysis at 3 months (OR = 0.20; 95% CI = 0.02–0.89; *p* = 0.045). Additionally, at this time, several *JAK2* SNPs showed associations with remission (rs7857730 (*p* = 0.002), rs2274472 (*p* = 0.002), and rs2230724 (*p* = 0.016)) (Table S35) while at 6 months, *JAK2* rs10119004 was associated with remission, specifically for AA (OR = 16.50; 95% CI = 2.19–201.83; *p* = 0.011) and AG genotypes (OR = 14.0; 95% CI = 2.12–138.41; *p* = 0.011) (Table S35). *JAK1* rs2230588 was associated with LDA at 6 months, with the C allele showing a higher likelihood of achieving LDA than the T allele (OR = 15.33; 95% CI = 2.20–311.97; *p* = 0.008) (Table S33).

In baricitinib-treated patients *JAK1* rs10889504 C allele carriers were more likely to achieve a satisfactory EULAR response at 3 months (OR = 6.13; 95% CI = 1.45–32.79; *p* = 0.013) (Table S37).

*JAK3* rs3008 was associated with both remission and LDA at 3 months, with the A allele showing lower probability of LDA (OR = 0.10; 95% CI = 0.01–0.72; *p* = 0.034) and higher remission frequencies (*p* = 0.042) (Tables S37 and S39). At 6 months, several *JAK1* variants, including rs310241 (OR = 0.20; 95% CI = 0.04–0.87; *p* = 0.033), rs2230588 (OR = 0.20; 95% CI = 0.04–0.87; *p* = 0.033), and rs2780815 (OR = 0.13; 95% CI = 0.02–0.73; *p* = 0.017), were associated with a lower probability of remission (Table S41). Additional borderline *JAK1* associations were observed at 6 months for EULAR response (Table S37).

In the upadacitinib cohort, *JAK2* rs2274472 C allele was associated with reduced odds of satisfactory response at 3 months (OR = 0.18; 95% CI = 0.03–0.91; *p* = 0.032) and lower remission probability (OR = 0.05; 95% CI = 0.01–0.40; *p* = 0.009) (Tables S47 and S51). Conversely, the *JAK2* rs2230722 T allele was associated with improved EULAR response (OR = 11.25; 95% CI = 2.26–73.48; *p* = 0.003) and remission (OR = 5.83; 95% CI = 1.20–35.07; *p* = 0.028) at 6 months (Tables S47 and S51). In addition, *JAK2* rs2230724 was consistently associated with LDA at both 3 and 6 months, with the A allele inversely associated with LDA achievement at 3 months (OR = 0.11; 95% CI = 0.01–1.00; *p* = 0.040) and remaining associated at 6 months (Table S51).

In filgotinib-treated patients, no consistent pharmacogenetic associations with EULAR response, LDA, or remission were identified, although a nominal association with *JAK3* rs3008 was observed at 3 months (Tables S41, S43 and S45).

Overall, *JAK2* variants showed the most recurrent exploratory signals across drugs and outcomes, whereas *JAK1* and *JAK3* signals were generally weaker, time-dependent, and less consistently replicated.

#### 3.4.3. Multivariable Analysis

Multivariable logistic regression analyses were performed to identify variables independently associated with EULAR response, LDA, and remission at 3 and 6 months for each JAK inhibitor (Table 3, Table 4 and Table 5). Associations with a nominal *p*-value < 0.05 but an FDR-adjusted *p*-value ≥ 0.05 were considered nominal and exploratory.

Overall, clinical and treatment-related variables showed greater consistency across drugs and follow-up assessments than individual pharmacogenetic variants, although several genetic associations remained statistically significant after FDR adjustment.

Regarding satisfactory EULAR response, lower baseline joint burden and selected metabolic parameters were associated with improved outcomes in the tofacitinib cohort.

At 3 months, lower baseline number of TJC (OR = 0.43; 95% CI = 0.18–0.70; *p* = 0.007; *p*-value [FDR] = 0.028) and lower TG levels (OR = 0.96; 95% CI = 0.93–0.99; *p* = 0.020; *p*-value [FDR] = 0.028) were associated with higher odds of satisfactory response. RF positivity was associated with markedly lower odds of response (OR = 0.05; 95% CI = 0.001–0.59; *p* = 0.030; *p*-value[FDR] = 0.030), whereas *JAK2* rs7857730 T-allele carriers showed higher odds of response (OR = 61.81; 95% CI = 3.45–7187.40; *p* = 0.020; *p*-value [FDR] = 0.028). All four associations remained significant after FDR adjustment, although the estimate for rs7857730 was highly imprecise. At 6 months, lower TJC (OR = 0.40; 95% CI = 0.13–0.81; *p* = 0.030) and lower LDL levels (OR = 0.93; 95% CI = 0.85–0.98; *p* = 0.040) showed nominal associations with satisfactory EULAR response, but neither remained significant after FDR adjustment (*p*-value [FDR] = 0.060 for both) (Table 3).

In the baricitinib cohort, absence of concomitant GC use was associated with satisfactory EULAR response at both 3 months (OR = 16.93; 95% CI = 3.30–121.74; *p* = 0.001; *p*-value [FDR] = 0.002) and 6 months (OR = 8.86; 95% CI = 1.75–63.42; *p* = 0.014; *p*-value [FDR] = 0.028). *JAK1* rs10889504 C-allele carriers also showed higher odds of satisfactory response at 3 months (OR = 12.16; 95% CI = 2.22–90.69; *p* = 0.007; *p*-value [FDR] = 0.007). At 6 months, higher BMI was associated with lower odds of satisfactory EULAR response (OR = 0.76; 95% CI = 0.57–0.96; *p* = 0.038; *p*-value [FDR] = 0.038). All these associations remained significant after FDR adjustment (Table 4).

In the upadacitinib cohort, *JAK2* rs2274472 C-allele carriers showed lower odds of satisfactory EULAR response at 3 months than TT homozygotes (OR = 0.06; 95% CI = 0.003–0.49; *p* = 0.022; *p*-value [FDR] = 0.044). At 6 months, absence of concomitant GC use (OR = 17.21; 95% CI = 2.07–397.74; *p* = 0.022; *p*-value [FDR] = 0.033) and *JAK2* rs2230722 T-allele carriage (OR = 21.45; 95% CI = 2.57–508.06; *p* = 0.014; *p*-value [FDR] = 0.033) were associated with higher odds of satisfactory response. Although these associations remained significant after FDR adjustment, the genetic and treatment-related estimates had wide confidence intervals and should therefore be interpreted cautiously (Table 5).

No predictors of LDA remained statistically significant after FDR adjustment. In the tofacitinib cohort (Table 3), the *JAK1* rs2230588 TT genotype showed a nominal association with lower odds of achieving LDA at 6 months compared with C-allele carriers (OR = 0.06; 95% CI = 0.002–0.64; *p* = 0.040; *p*-value [FDR] = 0.058). Similarly, in the upadacitinib cohort (Table 5), *JAK2* rs2230724 A-allele carriers showed nominally lower odds of achieving LDA at 3 months than GG homozygotes (OR = 0.06; 95% CI = 0.002–0.69; *p* = 0.042; *p*-value [FDR] = 0.084). These associations should therefore be regarded as exploratory.

For remission, several associations remained significant after FDR adjustment. In tofacitinib-treated patients (Table 3), lower baseline TC levels were associated with remission at 3 months (OR = 0.92; 95% CI = 0.83–0.97; *p* = 0.020; *p*-value [FDR] = 0.020), whereas *JAK2* rs2230724 A-allele carriers showed lower odds of remission (OR = 0.01; 95% CI = 0.00004–0.25; *p* = 0.020; *p*-value [FDR] = 0.020).

In baricitinib-treated patients, higher BMI was associated with lower odds of remission at 6 months (OR = 0.67; 95% CI = 0.44–0.91; *p* = 0.027; FDR = 0.041), whereas absence of concomitant GC use was associated with higher odds of remission (OR = 25.42; 95% CI = 3.30–391.82; *p* = 0.005; *p*-value [FDR] = 0.015).

In upadacitinib-treated patients, *JAK2* rs2274472 C-allele carriers showed lower odds of remission at 3 months than TT homozygotes (OR = 0.04; 95% CI = 0.001–0.40; *p* = 0.020; FDR = 0.040). At 6 months, absence of concomitant GC use (OR = 14.99; 95% CI = 1.76–350.19; *p* = 0.030; *p*-value [FDR] = 0.048), absence of concomitant vitamin D supplementation (OR = 21.60; 95% CI = 1.67–996.96; *p* = 0.048; *p*-value [FDR] = 0.048), and *JAK2* rs2230722 T-allele carriage (OR = 14.08; 95% CI = 1.56–347.57; *p* = 0.039; *p*-value [FDR] = 0.048) were associated with higher odds of remission. Although these associations remained significant after FDR adjustment, their wide confidence intervals indicate substantial imprecision. In particular, the vitamin D association may also reflect residual confounding and should be considered hypothesis-generating.

No independent predictors of EULAR response, LDA, or remission were identified in the filgotinib cohort after multivariable adjustment. Overall, the adjusted analyses indicate that clinical and treatment-related factors were more consistently associated with response across drugs and follow-up assessments, whereas the pharmacogenetic findings were drug-, outcome-, and time-specific and require validation in larger independent cohorts.

## 4. Discussion

JAK inhibitors have expanded the therapeutic options available to RA patients with inadequate responses to conventional or biologic DMARDs; however, the determinants of response to individual agents remain incompletely understood. In this study, we evaluated clinical, treatment-related, and pharmacogenetic factors associated with response to four JAK inhibitors at 3 and 6 months in real-world clinical practice. As regards the effectiveness of JAK inhibitors, lower baseline disease activity and inflammatory burden, concomitant treatment, BMI, and previous treatment exposure showed recurrent or drug-specific associations with response [14].

Clinical variables showed a relatively coherent pattern across the drug-specific cohorts. Lower baseline disease activity and lower inflammatory burden were recurrently associated with a higher probability of achieving satisfactory EULAR response, LDA, or remission. This observation is consistent with treat-to-target principles and with previous evidence showing that patients who start treatment with lower disease activity are more likely to achieve and sustain stricter outcomes over time (*p* < 0.05, Table 3 and Table 5). The persistence of these associations across models suggests baseline clinical status remains an important determinant of JAK inhibitor effectiveness [15,16].

Regarding biochemical markers, RF positivity was associated with a lower probability of satisfactory EULAR response to tofacitinib (*p*-value [FDR] = 0.030, Table 3) and with failure to achieve LDA with upadacitinib (OR = 16.20, 95% CI = 1.70–368.08, Table S50). These findings support the hypothesis that seropositivity may also condition the therapeutic response to JAK inhibitors. It has been established that seropositive RA is associated with greater disease severity, more joint damage, and a distinct immunopathological profile, which may translate into reduced responsiveness to targeted therapies [17].

In line with these observations, real-world evidence has also highlighted the central role of clinical context in shaping baricitinib effectiveness. In a multicenter registry study, Takahashi et al. reported that response to baricitinib at 24 weeks was primarily determined by lower baseline disease activity (OR = 0.28, 95% CI = 0.13–0.62) and by treatment positioning, with more favorable outcomes observed in targeted-DMARD–naïve patients (OR = 33.40, 95% CI= 2.53–442.62) and in those not receiving concomitant GC (OR = 0.24, 95% CI = 0.10–0.56) [15].

In the baricitinib cohort, absence of concomitant GC use was independently associated with satisfactory EULAR response and remission at both 3 and 6 months (*p*-value [FDR] < 0.05, Table 4 and Table S38). However, patients requiring concomitant GC tend to have higher baseline disease activity, reducing their probability of reaching remission regardless of the JAK inhibitor used, so this finding should be interpreted cautiously. This is a recognized limitation of observational pharmacological studies and further underscores the importance of multivariable adjustment in real-world analyses. On the other hand, the number of previous BTs was identified as a predictor of an unsatisfactory EULAR response to filgotinib at 3 months, in keeping with the well-established progressive loss of efficacy across lines of advanced therapy in RA. Notably, all patients treated with filgotinib in our cohort had previously received at least one other JAK inhibitor, which may partly explain the lower response rates observed with this drug compared to others in the series [18,19].

In addition, BMI appeared as a negative predictor of response to baricitinib, with higher BMI independently associated with a lower probability of a satisfactory EULAR response at 6 months (*p*-value [FDR] = 0.038, Table 4). Consistently, real-world data in patients treated with baricitinib or tofacitinib have shown that obesity is associated with a lower probability of achieving LDA within 6 months. The proinflammatory state associated with obesity, driven by adipokine dysregulation and chronic low-grade inflammation, may attenuate the anti-inflammatory effects of JAK inhibitors by maintaining cytokine-mediated pathway activation independently of the therapeutic target. Furthermore, previous evidence has shown that obesity in RA is associated with higher CRP and ESR levels, mainly reflecting adiposity rather than joint inflammatory activity itself, which may contribute to an unfavorable inflammatory background and to more difficult attainment of DAS28-based targets. In this context, our findings suggest that BMI may act as a clinically relevant modifier of JAK inhibitor effectiveness [20].

The pharmacogenetic findings should be interpreted within the context of prior genetic association research on the *JAK–STAT* pathway [21,22,23]. The most notable pharmacogenetic signals in our study were concentrated in the *JAK2* gene, particularly around the rs7857730 SNP and variants located within the same region. *JAK2* is a key intracellular tyrosine kinase involved in cytokine- and growth factor-mediated signaling, including pathways relevant to RA inflammation and JAK inhibitor activity. Specifically, the *JAK2* rs7857730 was independently associated with satisfactory EULAR response to tofacitinib at 3 months (T vs. GG: *p*-value [FDR] = 0.028, Table 3). Functional variability in this gene, potentially amplified by alternative splicing and the generation of distinct isoforms, may contribute to heterogeneity in drug response [22]. This intronic variant has previously been evaluated in immune-mediated diseases. Chen et al. reported that a *JAK2* haplotype including rs7857730 was less frequent in patients with ankylosing spondylitis (AS) than in healthy controls (5.5% vs. 9.5%; *p* = 0.0323), suggesting a possible involvement of this genomic region in inflammatory disease susceptibility. While these findings cannot be directly extrapolated to pharmacological response in RA, they provide biological support for further investigation of *JAK2* variation in the context of JAK inhibitor therapy [22].

Moreover, *JAK2* rs2230724, a synonymous variant, was associated with remission at 3 months in the tofacitinib cohort, with A-allele carriers showing lower odds of remission (*p*-value [FDR] = 0.030, Table 3). This variant also showed bivariate associations with LDA in the upadacitinib cohort (*p*-value = 0.040, Table S50). The SNP *JAK2* rs2230722, another synonymous variant, was associated with EULAR response (*p*-value = 0.003, Table S48) and remission (*p*-value = 0.028, Table S53) at 6 months in patients treated with upadacitinib, with T-allele carriers showing more favorable outcomes, whereas rs2274472 and rs10119004 showed more limited or non-independent associations [22,24]. This interpretation is supported by the LD structure observed in our cohort. *JAK2* rs7857730 and rs2230724 showed strong LD across treatment groups (D′ ≈ 1; r^2^ = 0.75–0.93 across cohorts). In line with this, Chen et al. previously reported a *JAK2* haplotype including rs10119004 and rs7857730 associated with ankylosing spondylitis susceptibility, raising the possibility that the observed associations are driven by a shared haplotypic background rather than by the individual SNPs [24,25,26].

The predominance of *JAK2*-related pharmacogenetic signals may also be considered in relation to the different pharmacological selectivity profiles of the evaluated JAK inhibitors. Baricitinib directly inhibits *JAK1* and *JAK2*, whereas tofacitinib has a broader JAK inhibition profile and upadacitinib and filgotinib preferentially inhibit *JAK1*. Nevertheless, the observed *JAK2* associations were not restricted to agents with direct or broader *JAK2* inhibition and did not show a consistent pattern. Differences in JAK selectivity may therefore represent one possible biological explanation, but these associations should consequently be regarded as exploratory and require external and functional validation [27].

In contrast to the *JAK2* findings, associations involving *JAK1* and *JAK3* variants were less consistent in our study. Given the selectivity profile of upadacitinib and filgotinib as preferential *JAK1* inhibitors, genetic variation in *JAK1* could contribute to variability in treatment response. However, in our cohort, *JAK1*-associated signals were heterogeneous across drugs and outcomes [26]. The SNP *JAK1* rs10889504 was associated with satisfactory EULAR response to baricitinib at 3 months (C vs. GG: *p*-value [FDR] = 0.007, Table 4), whereas rs2230588 was associated with LDA in the tofacitinib cohort (TT vs. C: *p*-value [FDR] = 0.058, Table 5). Other *JAK1* variants showed only nominal or bivariate associations that were not consistently retained after adjustment. The strong LD observed between rs2230588 and rs310241 in our cohort (D′ ≈ 0.88–1; r^2^ = 0.73–0.95) suggests that these signals may reflect a broader haplotypic background rather than independent variant-specific effects [24,28].

*JAK3* findings were limited to nominal associations involving rs3008 in baricitinib Tables S37 and S39) and filgotinib cohorts (Tables S41 and S43). Given the small number of genotypes and the instability of the estimates obtained across the different JAK inhibitors, further studies with larger cohorts would be needed to investigate the role of *JAK1* and *JAK3* variants as pharmacogenetic markers of response [23].

Several considerations should be taken into account when interpreting these findings. Although this is one of the few real-world studies integrating clinical, treatment-related, and pharmacogenetic factors associated with response to individual JAK inhibitors, some drug- and genotype-specific subgroups were small.

This is an inherent challenge in pharmacogenetic studies conducted in routine clinical practice, particularly when several treatments, outcomes, and follow-up time points are evaluated. Consequently, some effect estimates, especially those involving less frequent genotypes, were accompanied by wide confidence intervals and should be regarded as preliminary, hypothesis-generating signals requiring validation in larger independent cohorts.

The evaluation of multiple clinical, treatment-related, and genetic variables across different outcomes and follow-up assessments also increased the risk of chance findings. To reduce the risk of overinterpretation, multivariable results were adjusted using the Benjamini–Hochberg false discovery rate procedure, and associations that did not remain significant after adjustment were interpreted as nominal and exploratory. The limited replication of pharmacogenetic signals between 3 and 6 months may reflect differences between early and sustained response, treatment persistence, changes in concomitant therapy, and variation in the number of evaluable patients and genotype distribution at each assessment. Although this temporal heterogeneity does not establish or exclude a biological effect, it emphasizes the need for longitudinal validation and for considering treatment response as a dynamic phenotype.

Observational design reflects routine clinical practice and enhances the external relevance of the findings, although it does not allow causal inference or completely exclude residual confounding. Multivariable models were used to account for clinically relevant covariates, while the number of variables included in each model was restricted according to the available outcome events to avoid excessive model complexity. Some patients received different JAK inhibitors sequentially and could therefore contribute to more than one drug-specific cohort. However, each patient contributed only one treatment episode to each drug-specific analysis, and all inferential analyses were conducted separately for each JAK inhibitor. Accordingly, the study was designed to identify within-drug factors associated with response rather than to provide direct head-to-head comparisons between treatments.

Most participants had previously received biologic or targeted therapies. Although this limits the direct extrapolation of the findings to treatment-naïve populations, it also reflects the clinical setting in which JAK inhibitors are frequently used and provides evidence from a difficult-to-treat RA population that is often underrepresented in clinical trials. This was particularly relevant for filgotinib, as all patients in this cohort had received previous advanced therapies, including at least one prior JAK inhibitor. Treatment history may therefore have influenced the observed response rates through treatment-line and channeling effects. Nevertheless, this subgroup provides clinically relevant information on the effectiveness of filgotinib in highly treatment-experienced patients.

Finally, pharmacogenetic evidence regarding response to JAK inhibitors in RA remains limited, as most previous studies have focused on genetic susceptibility to immune-mediated diseases rather than on treatment outcomes. This lack of directly comparable evidence highlights both the novelty and the exploratory nature of the present study. By integrating clinical, treatment-related, and genetic variables across four JAK inhibitors, this study provides an initial framework for identifying potential drug-specific response markers. Larger multicenter studies incorporating independent replication, larger genotype subgroups, pharmacokinetic data, and functional assessment of *JAK/STAT* pathway activity are needed to determine the clinical relevance of the associations identified.

## 5. Conclusions

Patients with RA treated with JAK inhibitors showed better effectiveness when they had lower baseline disease activity, reduced inflammatory burden, a lower BMI, fewer previous treatment exposures, and concomitant therapy. Regarding the pharmacogenetic association, SNPs in the *JAK2* gene were most frequently associated with the effectiveness of JAK inhibitors (rs7857730, rs2230724, rs227447 and rs2230722). The results for variants in *JAK1* and *JAK3* were highly heterogeneous. Altogether, these findings underscore the multifactorial nature of treatment response in RA. Integrating both clinical baselines and specific *JAK2* pharmacogenetic markers could pave the way toward a more personalized approach, ultimately optimizing therapeutic selection and improving outcomes for patients treated with JAK inhibitors.

## Figures and Tables

**Table 1:** Sociodemographic and clinical characteristics of patients with RA treated with JAK inhibitors.

Variables (Full Cohort *n* = 115)			Initial Level	
	N	(%)	Reference Values	Mean ± SD/p_50_ (p_25_–p_75_)
**Sex**				
Women	94	81.7	-	-
**Number of treatment exposures to JAK inhibitor (150)**				
Tofacitinib	50	33.3	-	-
Baricitinib	44	29.3	-	-
Upadacitinib	36	24.1	-	-
Filgotinib	20	13.3	-	-
**Smoking**				
Smoker	20	17.3	-	-
Exsmoker	23	20	-	-
Non-smoker	72	62.6	-	-
Age at diagnosis (years)		-	-	43 (33–51)
Disease duration (years)		-	-	12 (8–20)
Time from diagnosis to JAK inhibitor initiation (years)		-	-	8.7 (4.3–15.6)
JAK inhibitor treatment duration (months)		-	-	36.1 (13.4–61.7)
BMI (kg/m^2^)		-	(18.5–24.9)	27.3 (24.3–31.2)
Number of previous BT		-	-	2 (1–3)
Duration of previous BT (years)		-	-	4.6 (1.4–10.7)
Baseline DAS28-CRP		-	**-**	4.5 ± 1.3
Baseline CRP (mg/L)		-	(0.4–5)	3.9 (1.2–9.3)
Baseline ESR (mm/h)		-	(1–10)	19 (9–35.5)
Baseline TC (mg/dL)		-	(120–200)	199.8 ± 39.7
Baseline LDL (mg/dL)		-	(10–115)	120.7 ± 31.5
Baseline TG (mg/dL)		-	(40–150)	95 (70.5–131.5)
Baseline PVAS		-	0–10	6 (5–8)
Baseline MVAS		-	0–10	5 (3–7)
Baseline RF (IU/mL)		-	(0–20)	-
Patients with positive RF (>20 IU/mL)	87	75.6	-	-
Patients previously treated with GC	110	95.6	-	-

Abbreviations: BMI, body mass index; BT, biologic therapy; CRP, C-reactive protein; DAS28, Disease Activity Score in 28 joints; ESR, erythrocyte sedimentation rate; GC, glucocorticoids; JAK, Janus kinase; LDL, low-density lipoprotein cholesterol; MVAS, physician visual analogue scale; PVAS, patient visual analogue scale; RA, rheumatoid arthritis; RF, rheumatoid factor; SD, standard deviation; TC, total cholesterol; TG, triglycerides. The full cohort comprised 115 patients. N denotes the number of patients with available data for the corresponding variable. For continuous variables, data are presented as mean ± SD or median (p25–p75), as appropriate. Reference values for laboratory variables were those established by the laboratory performing the analyses. The number of treatment exposures exceeds the total number of patients because some individuals received more than one JAK inhibitor sequentially during their disease management; therefore, patients could be included in more than one treatment group.

**Table 2:** Clinical effectiveness of JAK inhibitors for RA patients at 3 and 6 months.

	3 Months					6 Months		
Drugs	Evaluated Patients (n)	Satisfactory EULAR Response n (%)	LDA (2.6 ≤ DAS28 ≤ 3.2) n (%)	Remission (DAS28 < 2.6)	Evaluated Patients (n)	Satisfactory EULAR Response n (%)	LDA (2.6 ≤ DAS28 ≤ 3.2) n (%)	Remission (DAS28 < 2.6)
Tofacitinib	50	17 (34.0)	12 (24.0)	5 (10.0)	39	18 (46.2)	7 (17.9)	11 (28.2)
Baricitinib	44	18 (40.9)	5 (11.4)	13 (29.5)	36	15 (41.7)	7 (19.4)	12 (33.3)
Filgotinib	20	7 (35.0)	4 (20.0)	4 (20.0)	17	7 (41.2)	7 (41.2)	6 (35.3)
Upadacitinib	36	10 (27.8)	4 (11.1)	5 (13.9)	31	8 (25.8)	2 (6.5)	10 (32.3)

DAS28: disease activity score in 28 joints; EULAR: European Alliance of Associations for Rheumatology criteria; LDA: low-activity disease. No biologic-naïve patients were included in the filgotinib cohort.

**Table 3:** Predictors of response at 3 and 6 months in patients with RA treated with tofacitinib: multivariable analysis.

Independent Variable	B	OR	*p*-Value (Variable)	95% CI	R^2^	Goodness of Fit	*p*-Value [FDR]
**3 MONTHS**							
**EULAR response**							
TJC	−0.84	0.43	0.007	[0.18–0.70]	Cox Snell R^2^ = 0.502 Nagelkerke R^2^ = 0.694	X^2^ = 1.872 *p* = 0.984	0.028
TG levels	−0.03	0.96	0.020	[0.93–0.99]			0.028
*JAK2* rs7857730 (T carriers)	4.12	61.81	0.020	[3.45–7187.4]			0.028
RF positivity	−2.95	0.05	0.030	[0.001–0.59]			0.030
**Remission**							
TC levels	−0.07	0.92	0.020	[0.83–0.97]	Cox Snell R^2^ =0.308 Nagelkerke R^2^ = 0.644	X^2^ = 0.750 *p* = 0.999	0.020
*JAK2* rs2230724 (A carriers)	−4.47	0.01	0.020	[0.00004–0.25]			0.020
**6 MONTHS**							
**EULAR response**							
TJC	−0.91	0.40	0.030	[0.13–0.81]	Cox Snell R^2^ = 0.598 Nagelkerke R^2^ = 0.799	X^2^ = 2.881 *p* = 0.941	0.060
LDL levels	−0.06	0.93	0.040	[0.85–0.98]			0.060
SJC	−2.56	0.07	0.080	[0.001–0.66]			0.080
**LDA**							
ESR value	−0.17	0.84	0.058	[0.65–0.95]	Cox Snell R^2^ = 0.343 Nagelkerke R^2^ = 0.563	X^2^ = 5.456 *p* = 0.707	0.058
*JAK1* rs2230588 (TT vs. C carriers)	−2.68	0.06	0.040	[0.002–0.64]			0.058

TJC: tender joint count; TG: triglycerides; TC: total cholesterol; LDL: low-density lipoprotein; ESR: erythrocyte sedimentation rate; SJC: Swollen Joint Count; OR: odds ratio; CI: confidence interval; B: logistic regression coefficient; R^2^: Cox–Snell and Nagelkerke pseudo-R^2^ measures; Goodness of Fit: Hosmer–Lemeshow χ^2^ and *p*-value; *p*-value [FDR]: False Discovery Rate.

**Table 4:** Predictors of response at 3 and 6 months in patients with RA treated with baricitinib: multivariable analysis.

Independent Variable	B	OR	*p*-Value (Variable)	95% CI	R^2^	Goodness of Fit	*p*-Value [FDR]
**3 MONTHS**							
**EULAR response**							
Concomitant GC (no vs. yes)	2.82	16.93	0.001	3.30–121.74	Cox Snell R^2^ =0.346 Nagelkerke R^2^ = 0.467	X^2^ = NA *p* = NA	0.002
*JAK1* rs10889504 (C carriers vs. GG)	2.49	12.16	0.007	2.22–90.69			0.007
**6 MONTHS**							
**EULAR response**							
Concomitant GC (no vs. yes)	2.18	8.86	0.014	1.75–63.42	Cox Snell R^2^ =0.281 Nagelkerke R^2^ = 0.379	X^2^ = 7.165 *p* = 0.518	0.028
BMI	−0.27	0.76	0.038	0.57–0.96			0.038
**Remission**							
BMI	−0.39	0.67	0.027	0.44–0.91	Cox Snell R^2^ =0.439 Nagelkerke R^2^ = 0.610	X^2^ = 15.318 *p* = 0.053	0.041
Concomitant GC (no vs. yes)	3.23	25.42	0.005	3.30–391.82			0.015
*JAK1* rs2780815 (G vs. TT)	−1.55	0.21	0.190	0.01–2.19			0.190

GC: glucocorticoids; BMI: Body mass index; NA: not available (indicates non-estimable values due to sparse data or quasi-complete separation); OR: odds ratio; CI: confidence interval; B: logistic regression coefficient; R^2^: Cox–Snell and Nagelkerke pseudo-R^2^ measures; Goodness of Fit: Hosmer–Lemeshow χ^2^ and *p*-value; *p*-value [FDR]: False Discovery Rate.

**Table 5:** Predictors of response at 3 and 6 months in patients with RA treated with upadacitinib: multivariable analysis.

Independent Variable	B	OR	*p*-Value (Variable)	95% CI	R^2^	Goodness of Fit	*p*-Value [FDR]
**3 MONTHS**							
**EULAR response**							
Years of RA	−0.171	0.84	0.051	0.68–0.97	Cox Snell R^2^ =0.256 Nagelkerke R^2^ = 0.369	X^2^ = 1.735 *p* = 0.988	0.051
*JAK2* rs2274472 (C vs. TT)	−2.770	0.06	0.022	0.003–0.49			0.044
**LDA**							
CRP	−0.277	0.76	0.164	0.42–0.96	Cox Snell R^2^ =0.226 Nagelkerke R^2^ = 0.450	X^2^ = 5.543 *p* = 0.698	0.164
*JAK2* rs2230724 (A vs. GG)	−2.839	0.06	0.042	0.002–0.69			0.084
**Remission**							
BMI	0.211	1.24	0.085	0.98–1.65	Cox Snell R^2^ = 0.270 Nagelkerke R^2^ = 0.489	X^2^ = 4.839 *p* = 0.774	0.085
*JAK2* rs2274472 (C vs. TT)	−3.275	0.04	0.020	0.001–0.40			0.040
**6 MONTHS**							
**EULAR response**							
Concomitant GC (No vs. Yes)	2.85	17.21	0.022	2.07–397.74	Cox Snell R^2^ =0.527 Nagelkerke R^2^ = 0.715	X^2^ = 3.668 *p* = 0.885	0.033
ESR	−0.067	0.93	0.157	0.83–1.01			0.157
*JAK2* rs2230722 (T vs. CC)	3.07	21.45	0.014	2.57–508.06			0.033
**Remission**							
Concomitant vitamin D (No vs. Yes)	3.073	21.60	0.048	1.67–996.96	Cox Snell R^2^ = 0.434 Nagelkerke R^2^ = 0.606	X^2^ = 2.793 *p* = 0.946	0.048
Concomitant GC (No vs. Yes)	2.707	14.99	0.030	1.76–350.19			0.048
*JAK2* rs2230722 (T vs. CC)	2.645	14.08	0.039	1.56–347.57			0.048

RA: rheumatoid arthritis; ESR: Erythrocyte sedimentation rate; BMI: body mass index; GC: GlucocorticoidsGenetic associations are presented according to dominant or recessive inheritance models. OR: odds ratio; CI: confidence interval; B: logistic regression coefficient; R^2^: Cox–Snell and Nagelkerke pseudo-R^2^ measures; Goodness of Fit: Hosmer–Lemeshow χ^2^ and *p*-value; *p*-value [FDR]: False Discovery Rate.

## Data Availability

The original contributions presented in this study are included in the article/Supplementary Materials. Further inquiries can be directed to the corresponding authors.

## Supplementary Materials

The following supporting information can be downloaded at: https://www.mdpi.com/article/10.3390/pharmaceutics18070846/s1. Table S1: Genomic characteristics, minor allele frequencies, previous evidence, and rationale for selection of the analyzed SNPs; Table S2. Clinical effectiveness of JAK inhibitors for RA bio-naïve patients at 3 and 6 months; Table S3: Hardy–Weinberg Equilibrium Results; Table S4: Minor allele frequencies of SNPs; Table S5: Linkage disequilibrium; Table S6: Haplotype frequency estimation EULAR response at 3 months of Baricitinib; Table S7: Haplotype frequency estimation LDA at 3 months of Baricitinib; Table S8: Haplotype frequency estimation remission at 3 months of Baricitinib; Table S9: Haplotype frequency estimation EULAR response at 6 months of Baricitinib; Table S10: Haplotype frequency estimation LDA at 6 months of Baricitinib; Table S11: Haplotype frequency estimation of remission at 6 months Baricitinib; Table S12: Haplotype frequency estimation EULAR response at 3 months filgotinib; Table S13: Haplotype frequency estimation LDA at 3 months filgotinib; Table S14: Haplotype frequency estimation remission at 3 months filgotinib; Table S15: Haplotype frequency estimation EULAR response at 6 months filgotinib; Table S16: Haplotype frequency estimation LDA at 6 months Filgotinib; Table S17: Haplotype frequency estimation remission at 6 months Filgotinib; Table S18: Haplotype frequency estimation EULAR response at 3 months of Tofacitinib; Table S19: Haplotype frequency estimation LDA at 3 months Tofacitinib; Table S20: Haplotype frequency estimation remission at 3 months of Tofacitinib; Table S21: Haplotype frequency EULAR response at 6 months of Tofacitinib; Table S22: Haplotype frequency LDA at 6 months of Tofacitinib; Table S23: Haplotype frequency estimation Remission at 6 months of Tofacitinib; Table S24: Haplotype frequency estimation EULAR response at 3 months of Upadacitinib; Table S25: Haplotype frequency estimation LDA at 3 months of Upadacitinib; Table S26: Haplotype frequency Remission at 3 months of Upadacitinib; Table S27: Haplotype frequency estimation EULAR response at 6 months of Upadacitinib; Table S28: Haplotype frequency estimation LDA at 6 months of Upadacitinib; Table S29: Haplotype frequency estimation Remission at 6 months of Upadacitinib; Table S30: Tofacitinib EULAR response bivariate demographic and clinical analyses; Table S31: Tofacitinib EULAR response bivariate genetic analyses; Table S32: Tofacitinib LDA bivariate demographic and clinical analyses; Table S33: Tofacitinib LDA bivariate genetic analysis; Table S34: Tofacitinib remission bivariate demographic and clinical analyses; Table S35: Tofacitinib remission bivariate genetic analysis; Table S36: Baricitinib EULAR response bivariate demographic and clinical analyses; Table S37: Baricitinib EULAR response bivariate genetic analyses; Table S38: Baricitinib LDA bivariate demographic and clinical analyses; Table S39: Baricitinib LDA bivariate genetic analyses; Table S40: Baricitinib remission bivariate demographic and clinical analyses; Table S41: Baricitinib remission bivariate genetic analyses; Table S42: Filgotinib EULAR response bivariate demographic and clinical analyses; Table S43: Filgotinib EULAR response bivariate genetic analyses; Table S44: Filgotinib LDA bivariate demographic and clinical analyses; Table S45: Filgotinib LDA bivariate genetic analyses; Table S46: Filgotinib remission bivariate demographic and clinical analyses; Table S47: Filgotinib remission bivariate genetic analyses; Table S48: Upadacitinib EULAR response bivariate demographic and clinical analyses; Table S49: Upadacitinib EULAR response bivariate genetic analyses; Table S50: Upadacitinib LDA bivariate demographic and clinical analyses; Table S51: Upadacitinib LDA bivariate genetic analyses; Table S52: Upadacitinib remission bivariate demographic and clinical analyses; Table S53: Upadacitinib remission bivariate genetic analyses.

## Abbreviations

The following abbreviations are used in this manuscript:

ACPA	Anti-citrullinated protein antibodies
AE	Adverse event
AS	Ankylosing spondylitis
AUC	Area under the curve
bDMARD	Biological disease-modifying antirheumatic drug
BMI	Body mass index
BT	Biologic therapy
CCP	Cyclic citrullinated peptide
CI	Confidence interval
CRP	C-reactive protein
csDMARD	Conventional synthetic disease-modifying antirheumatic drug
DAS28	Disease Activity Score in 28 joints
DMARD	Disease-modifying antirheumatic drug
EULAR	European Alliance of Associations for Rheumatology
ESR	Erythrocyte sedimentation rate
eQTL	expression Quantitative Trait Loci
FLS	Fibroblast-like synoviocyte
GC	Glucocorticoid
GWAS	Genome-wide association study
HLA	Human leukocyte antigen
IL	Interleukin
IRB	Institutional Review Board
JAK	Janus kinase
JAK inhibitors	Janus kinase inhibitor
LDA	Low disease activity
LD	Linkage Disequilibrium
MAF	Minor allele frequency
MHC	Major histocompatibility complex
MTX	Methotrexate
MVAS	Physician visual analogue scale
NSAID	non-steroidal anti-inflammatory drug
NA	Not available (indicates non-estimable values due to sparse data or quasi-complete separation)
OR	Odds ratio
PCR	Polymerase chain reaction
PRS	Polygenic risk score
PVAS	Patient visual analogue scale
TJC	Tender joint count
RA	Rheumatoid arthritis
RF	Rheumatoid factor
SD	Standard deviation
SNP	Single-nucleotide polymorphism
STAT	Signal transducer and activator of transcription
SJC	Swollen joint count.
TNF	Tumor necrosis factor
TNFi	Tumor necrosis factor inhibitor
tsDMARD	Targeted synthetic disease-modifying antirheumatic drug
Th17	T helper 17 cells
*TYK2*	Tyrosine Kinase 2
