# Supplementary material for: Clinical and Pharmacogenetic Factors Associated with Response to JAK Inhibitors in Patients with Rheumatoid Arthritis: A Real-World Study of JAK1, JAK2, and JAK3 Gene Variants
Source: Pharmaceutics. 2026 Jul 11;18(7):846. doi: 10.3390/pharmaceutics18070846 (PMC13415438; doi:10.3390/pharmaceutics18070846)
Supplement: Supplementary file 1 [file pharmaceutics-18-00846-s001.zip › Table S1. Genomic characteristics, minor allele frequencies and rationale for selection of the analyzed SNPs.pdf]

Table SNP. Genomic characteristics, minor allele frequencies, previous evidence, and rationale for selection of the analyzed SNPs

Gene	SNP	Chromosome	Genomic position (GRCh38)*	Alleles	Minor allele	MAF—Tofacitinib	MAF—Baricitinib	MAF—Filgotinib	MAF—Upadacitinib	MAF (ALFA)	Functional annotation	Previous evidence used for selection	Selection rationale	TaqMan assay ID
JAK1	rs2230587	1	64845579	A/G	A	0.09	0.17	0.12	0.13	A: 0.130	missense variants	Investigated in a JAK–STAT pathway candidate-gene study of ankylosing spondylitis (Deng et al., 2023).	Candidate JAK1 variant with potential protein-level relevance; prior immune-mediated disease evidence; adequate population frequency; validated TaqMan assay available.	C___1766240_20
	rs2230588		64844806	C/T	C	0.22	0.26	0.20	0.20	C: 0.300	synonymous variant	Investigated in a JAK–STAT pathway candidate-gene study of ankylosing spondylitis (Deng et al., 2023).	Candidate JAK1 variant with prior immune-mediated disease evidence; potential regulatory relevance; adequate frequency; validated TaqMan assay available.	C__22273175_10
	rs2780815		64835928	G/T	G	0.46	0.45	0.40	0.40	G: 0.4879	intronic variant	Investigated in a JAK–STAT pathway candidate-gene study of ankylosing spondylitis (Deng et al., 2023).	Candidate intronic JAK1 variant with prior pathway-related evidence, common population frequency, and validated TaqMan assay.	C___1766225_20
	rs10889504		64924820	C/G	C	0.10	0.14	0.07	0.13	C: 0.174	intronic variant	Associated with juvenile idiopathic arthritis susceptibility in a genome-wide association meta-analysis (McIntosh et al., 2017).	Prior association with inflammatory arthritis; candidate regulatory JAK1 locus; adequate frequency; validated TaqMan assay available.	C___1766309_10
	rs310241		64837655	A/G	G	0.22	0.27	0.20	0.25	G: 0.252	intronic variant	Associated with psoriasis susceptibility and investigated in ankylosing spondylitis (Sayed et al., 2020; Deng et al., 2023).	Prior immune-mediated disease association and pathway relevance; common frequency; validated TaqMan assay available.	C___1767439_10
JAK2	rs10119004	9	5071049	A/G	A	0.47	0.46	0.47	0.50	G: 0.426	intronic variant	Evaluated as part of a JAK2 haplotype associated with ankylosing spondylitis susceptibility (Chen et al., 2010; Deng et al., 2023).	Candidate JAK2 haplotype marker with prior immune-mediated disease evidence, common frequency, and validated TaqMan assay.	C__30016880_10
	rs7857730		5084049	A/G	G	0.50	0.38	0.45	0.45	G: 0.358	intronic variant	Evaluated within JAK2 haplotypes associated with ankylosing spondylitis susceptibility (Chen et al., 2010; Deng et al., 2023).	Candidate JAK2 haplotype marker with prior inflammatory disease evidence, common frequency, and validated TaqMan assay.	C__29340600_20
	rs2274472		4985542	C/T	C	0.47	0.34	0.45	0.44	C: 0.302	intronic variant	Investigated in a JAK–STAT pathway candidate-gene study of ankylosing spondylitis (Deng et al., 2023).	Candidate intronic JAK2 variant previously investigated in a JAK–STAT pathway association study; adequate population frequency and validated TaqMan assay available.	C__16181933_10
	rs2230722		5050706	C/T	T	0.21	0.21	0.30	0.25	T: 0.330	synonymous variant	Investigated in a JAK–STAT pathway candidate-gene study of ankylosing spondylitis (Deng et al., 2023).	Candidate JAK2 coding-region marker with prior pathway-related evidence, adequate frequency, and validated TaqMan assay.	C__30502833_10
	rs2230724		5081780	G/A	G	0.45	0.42	0.47	0.48	G: 0.481	synonymous variant	Previously associated with gastric cancer risk and investigated in ankylosing spondylitis (Yang et al., 2013; Deng et al., 2023).	Candidate JAK2 marker with prior disease-association evidence, common frequency, and validated TaqMan assay.	C__22273141_10
JAK3	rs3212780	19	17830033	A/G	A	0.28	0.36	0.32	0.23	A: 0.222	intronic variant	Previously evaluated in 2,136 Spanish patients with rheumatoid arthritis for cardiovascular disease risk (García-Bermúdez et al., 2015).	Direct evidence in a large Spanish RA cohort; candidate JAK3 locus; adequate frequency; validated TaqMan assay available.	C__32396501_10
	rs3212752		17837923	C/T	C	0.06	0.07	0.02	0.05	C: 0.080	intronic variant	Previously evaluated in 2,136 Spanish patients with rheumatoid arthritis for cardiovascular disease risk (García-Bermúdez et al., 2015).	Direct evidence in a large Spanish RA cohort and inclusion in the predefined JAK3 candidate panel; low frequency limits power in some cohorts.	C__32396521_10
	rs3008		17826620	A/G	A	0.46	0.40	0.40	0.45	G: 0.493	3′-UTR variant	Associated with psoriasis susceptibility (Sayed et al., 2020).	Prior immune-mediated disease association and potential regulatory relevance; common frequency; validated TaqMan assay available.	C___2677324_10

SNP: Single Nucleotide Polymorphism; UTR: Untranslated Region; MAF: Minor Allele Frequency; rsID: Reference SNP cluster ID; *JAK1, JAK2, JAK3*: Janus Kinase genes 1, 2, and 3; GRCh38 (Genome Reference Consortium Human Build 38); ALFA: Allele Frequency Aggregator. Genomic positions and reference allele frequencies (European population) were retrieved from the NCBI dbSNP database using the GRCh38 assembly.
