# Supplementary material for: Clinical and Pharmacogenetic Factors Associated with Response to JAK Inhibitors in Patients with Rheumatoid Arthritis: A Real-World Study of JAK1, JAK2, and JAK3 Gene Variants
Source: Pharmaceutics. 2026 Jul 11;18(7):846. doi: 10.3390/pharmaceutics18070846 (PMC13415438; doi:10.3390/pharmaceutics18070846)
Supplement: Supplementary file 1 [file pharmaceutics-18-00846-s001.zip › Table S12-S17.Haplotype frequency estimation EULAR response, LDA, Remission at 3 and 6 months Filgotinib.pdf]

Table S12. Haplotype frequency estimation EULAR response at 3 months filgotinib								
JAK1 SNPs					EULAR Response			
**rs2230587**	**rs310241**	**rs2230588**	**rs10889504**	**rs2780815**	**Total**	**Satisfactory**	**Unsatisfactory**	**Cumulative frequency**
G	A	T	G	T	0.575	0.642	0.538	0.575
G	G	C	G	G	0.175	0.214	0.153	0.750
G	A	T	G	G	0.100	NA	0.115	0.850
A	A	T	C	G	0.075	NA	0.115	0.925
A	A	T	G	G	0.025	0.071	0.038	0.950
G	A	C	G	T	0.025	NA	0.038	0.975
A	G	T	G	G	0.025	NA	NA	1
G	G	T	G	G	0	0.071	NA	1
JAK2 SNPs					EULAR Response			
**rs10119004**	**rs7857730**	**rs2274472**	**rs2230722**	**rs2230724**	**Total**	**Satisfactory**	**Unsatisfactory**	**Cumulative frequency**
G	G	C	C	G	0.231	0.278	0.236	0.231
A	T	T	T	A	0.180	0.207	0.156	0.412
A	T	T	C	A	0.159	0.071	0.218	0.571
G	G	T	C	G	0.101	0.078	0.073	0.672
G	G	C	T	G	0.063	NA	0.106	0.736
A	T	C	C	A	0.053	0.071	0.040	0.789
G	T	T	C	A	0.051	0.071	0.038	0.840
A	G	C	C	G	0.047	0.071	NA	0.888
A	T	C	T	A	0.028	0.078	NA	0.917
G	T	T	T	A	0.026	NA	0.044	0.944
G	T	C	C	A	0.025	NA	0.039	0.969
G	T	T	C	G	0.025	0.071	NA	0.994
A	G	T	C	G	0.005	NA	0.045	1
JAK3 SNPs					EULAR Response			
**rs3212780**	**rs3008**		**rs3212752**		**Total**	**Satisfactory**	**Unsatisfactory**	**Cumulative frequency**
G	G		T		0.351	0.500	0.359	0.351
G	A		T		0.298	0.285	0.217	0.650
A	G		T		0.248	0	0.294	0.898
A	A		T		0.076	0.214	0.089	0.975
G	A		C		0.025	NA	0.038	1
G	G		C		0	NA	0	1

SNP: single nucleotide polymorphism; JAK: Janus kinase; NA: not available (frequency could not be estimated due to low counts); Total: overall haplotype frequency in the study population. EULAR: European Alliance of Associations for Rheumatology; Satisfactory/Unsatisfactory: clinical response categories according to EULAR criteria.

Table S13. Haplotype frequency estimation LDA at 3 months filgotinib								
JAK1 SNPs					LDA			
**rs2230587**	**rs310241**	**rs2230588**	**rs10889504**	**rs2780815**	**Total**	**LDA**	**No LDA**	**Cumulative frequency**
G	A	T	G	T	0.575	0.625	0.562	0.575
G	G	C	G	G	0.175	0.125	0.187	0.750
G	A	T	G	G	0.100	NA	0.093	0.850
A	A	T	C	G	0.075	NA	0.093	0.925
A	A	T	G	G	0.025	0.125	0.031	0.950
G	A	C	G	T	0.025	NA	0.031	0.975
A	G	T	G	G	0.025	NA	NA	1
JAK2 SNPs					LDA			
**rs10119004**	**rs7857730**	**rs2274472**	**rs2230722**	**rs2230724**	**Total**	**LDA**	**No LDA**	**Cumulative frequency**
G	G	C	C	G	0.231	NA	0.230	0.231
A	T	T	T	A	0.180	NA	0.204	0.412
A	T	T	C	A	0.159	0.125	0.164	0.571
G	G	T	C	G	0.101	0.250	0.128	0.672
G	G	C	T	G	0.063	NA	0.075	0.736
A	T	C	C	A	0.053	0.125	0.034	0.789
G	T	T	C	A	0.051	0.125	0.032	0.840
A	G	C	C	G	0.048	NA	0.066	0.888
A	T	C	T	A	0.028	0.250	NA	0.917
G	T	T	T	A	0.026	NA	0.033	0.944
G	T	C	C	A	0.025	NA	0.031	0.969
G	T	T	C	G	0.025	NA	NA	0.994
A	G	T	C	G	0.005	NA	0	1
G	T	C	C	G	0	0.125	NA	1
G	G	T	T	G	0	NA	NA	1
JAK3 SNPs					LDA			

rs3212780	rs3008	rs3212752	Total	LDA	No LDA	Cumulative frequency
G	G	T	0.351	0.500	0.340	0.351
G	A	T	0.298	0.375	0.253	0.650
A	G	T	0.248	0	0.284	0.898
A	A	T	0.076	0.125	0.090	0.975
G	A	C	0.025	NA	0.031	1
G	G	C	0	NA	0	1
SNP: single nucleotide polymorphism; JAK: Janus kinase; NA: not available (frequency could not be estimated due to low counts); Total: overall haplotype frequency in the study population; LDA: low disease activity						

**Table S14. Haplotype frequency estimation remission at 3 months filgotinib**								
JAK1 SNPs					Remission			
**rs2230587**	**rs310241**	**rs2230588**	**rs10889504**	**rs2780815**	**Total**	**Remission**	**No remission**	**Cumulative frequency**
G	A	T	G	T	0.575	0.625	0.562	0.575
G	G	C	G	G	0.175	0.375	0.125	0.750
G	A	T	G	G	0.100	NA	0.125	0.850
A	A	T	C	G	0.075	NA	0.093	0.925
A	A	T	G	G	0.025	NA	0.031	0.950
G	A	C	G	T	0.025	NA	0.031	0.975
A	G	T	G	G	0.025	NA	0.031	1
G	G	T	G	G	0	NA	0	1
JAK2 SNPs					Remission			
**rs10119004**	**rs7857730**	**rs2274472**	**rs2230722**	**rs2230724**	**Total**	**Remission**	**No remission**	**Cumulative frequency**
G	G	C	C	G	0.231	0.250	0.247	0.231
A	T	T	T	A	0.180	0.250	0.153	0.411
A	T	T	C	A	0.159	0.125	0.179	0.570
G	G	T	C	G	0.102	0.125	0.065	0.672
G	G	C	T	G	0.063	NA	0.089	0.736
A	T	C	C	A	0.053	NA	0.065	0.789
G	T	T	C	A	0.051	0.125	0.031	0.840
A	G	C	C	G	0.048	0.125	NA	0.889
A	T	C	T	A	0.028	NA	0.035	0.918
G	T	T	T	A	0.026	NA	0.034	0.944
G	T	C	C	A	0.025	NA	0.032	0.970
G	T	T	C	G	0.025	NA	0.031	0.995
A	G	T	C	G	0.005	NA	0.035	1
G	T	C	C	G	0	NA	NA	1
G	G	T	T	G	0	NA	0	1
JAK3 SNPs					Remission			
**rs3212780**	**rs3008**		**rs3212752**		**Total**	**Remission**	**No remission**	**Cumulative frequency**
G	G		T		0.351	0.250	0.362	0.351
G	A		T		0.298	0.500	0.262	0.650
A	G		T		0.248	0.250	0.262	0.898
A	A		T		0.076	0	0.081	0.975
G	A		C		0.025	NA	0.031	1
G	G		C		0	NA	0	1
SNP: single nucleotide polymorphism; JAK: Janus kinase; NA: not available (frequency could not be estimated due to low counts); Total: overall haplotype frequency in the study population.								

**Table S15. Haplotype frequency estimation EULAR response at 6 months filgotinib**								
JAK1 SNPs					EULAR Response			
**rs2230587**	**rs310241**	**rs2230588**	**rs10889504**	**rs2780815**	**Total**	**Satisfactory**	**Unsatisfactory**	**Cumulative frequency**
G	A	T	G	T	0.529	0.550	0.500	0.529
G	G	C	G	G	0.176	0.200	0.142	0.705
G	A	T	G	G	0.117	0.100	0.142	0.823
A	A	T	C	G	0.088	0.050	0.142	0.911
A	A	T	G	G	0.029	NA	0.071	0.941
G	A	C	G	T	0.029	0.050	NA	0.970
A	G	T	G	G	0.029	0.050	NA	1
G	G	T	G	G	0	NA	NA	1
A	A	T	C	T	0	0	0	1
JAK2 SNPs					EULAR Response			
**rs10119004**	**rs7857730**	**rs2274472**	**rs2230722**	**rs2230724**	**Total**	**Satisfactory**	**Unsatisfactory**	**Cumulative frequency**
G	G	C	C	G	0.246	0.337	0.095	0.246
A	T	T	T	A	0.187	0.187	0.167	0.433
A	T	T	C	A	0.156	0.108	0.214	0.589
G	G	T	C	G	0.123	0.050	0.261	0.713

A	G	C	C	G	0.061	0.054	0.071	0.774
A	T	C	C	A	0.058	0.050	0.071	0.833
G	G	C	T	G	0.038	0.058	NA	0.872
A	T	C	T	A	0.036	NA	0.118	0.908
G	T	T	T	A	0.032	0.054	NA	0.940
G	T	T	C	A	0.029	0.050	NA	0.970
G	T	T	C	G	0.029	0.050	NA	0.999
A	G	T	C	G	6e-04	NA	0	1
SNP: single nucleotide polymorphism; JAK: Janus kinase; NA: not available (frequency could not be estimated due to low counts); Total: overall haplotype frequency in the study population. EULAR: European Alliance of Associations for Rheumatology; Satisfactory/Unsatisfactory: clinical response categories according to EULAR criteria.								

Table S16. Haplotype frequency estimation LDA at 6 months Filgotinib

JAK1 SNPs					LDA			
**rs2230587**	**rs310241**	**rs2230588**	**rs10889504**	**rs2780815**	**Total**	**LDA**	**No LDA**	**Cumulative frequency**
G	A	T	G	T	0.529	0.625	0.500	0.529
G	G	C	G	G	0.176	0.125	0.192	0.705
G	A	T	G	G	0.117	NA	0.153	0.823
A	A	T	C	G	0.088	0.125	0.076	0.911
A	A	T	G	G	0.029	NA	0.038	0.941
G	A	C	G	T	0.029	0.125	NA	0.970
A	G	T	G	G	0.029	NA	0.038	1
G	G	T	G	G	0	0	0	1
***JAK2* SNPs**					**LDA**			
**rs10119004**	**rs7857730**	**rs2274472**	**rs2230722**	**rs2230724**	**Total**	**LDA**	**No LDA**	**Cumulative frequency**
G	G	C	C	G	0.246	0.500	0.162	0.246
A	T	T	T	A	0.187	0.125	0.201	0.433
A	T	T	C	A	0.156	0.125	0.166	0.589
G	G	T	C	G	0.123	NA	0.166	0.713
A	G	C	C	G	0.061	NA	0.082	0.774
A	T	C	C	A	0.058	0.125	0.038	0.833
G	G	C	T	G	0.038	0	0.050	0.872
A	T	C	T	A	0.036	NA	0.050	0.908
G	T	T	T	A	0.032	NA	0.043	0.940
G	T	T	C	A	0.029	NA	0.038	0.970
G	T	T	C	G	0.029	0.125	NA	0.999
A	G	T	C	G	6e-04	NA	0	1
SNP: single nucleotide polymorphism; JAK: Janus kinase; NA: not available (frequency could not be estimated due to low counts); Total: overall haplotype frequency in the study population; LDA: low disease activity								

Table S17. Haplotype frequency estimation remission at 6 months Filgotinib

JAK1 SNPs					Remission			
**rs2230587**	**rs310241**	**rs2230588**	**rs10889504**	**rs2780815**	**Total**	**Remission**	**No remission**	**Cumulative frequency**
G	A	T	G	T	0.529	0.500	0.545	0.529
G	G	C	G	G	0.176	0.250	0.136	0.705
G	A	T	G	G	0.117	0.166	0.090	0.823
A	A	T	C	G	0.088	NA	0.136	0.911
A	A	T	G	G	0.029	NA	0.045	0.941
G	A	C	G	T	0.029	NA	0.045	0.970
A	G	T	G	G	0.029	0.083	NA	1
G	G	T	G	G	0	NA	NA	1
***JAK2* SNPs**					**Remission**			
**rs10119004**	**rs7857730**	**rs2274472**	**rs2230722**	**rs2230724**	**Total**	**Remission**	**No remission**	**Cumulative frequency**
G	G	C	C	G	0.246	0.229	0.310	0.246
A	T	T	T	A	0.187	0.229	0.173	0.433
A	T	T	C	A	0.156	0.093	0.181	0.589
G	G	T	C	G	0.123	0.083	0.099	0.713
A	G	C	C	G	0.061	0.093	NA	0.774
A	T	C	C	A	0.058	NA	0.090	0.833
G	G	C	T	G	0.038	0.093	NA	0.872
A	T	C	T	A	0.036	NA	0.053	0.908
G	T	T	T	A	0.032	0.093	NA	0.940
G	T	T	C	A	0.029	0.083	NA	0.970
G	T	T	C	G	0.029	NA	0.045	0.999
A	G	T	C	G	6e-04	NA	0.045	1
SNP: single nucleotide polymorphism; JAK: Janus kinase; NA: not available (frequency could not be estimated due to low counts); Total: overall haplotype frequency in the study population.								
