# Supplementary material for: Clinical and Pharmacogenetic Factors Associated with Response to JAK Inhibitors in Patients with Rheumatoid Arthritis: A Real-World Study of JAK1, JAK2, and JAK3 Gene Variants
Source: Pharmaceutics. 2026 Jul 11;18(7):846. doi: 10.3390/pharmaceutics18070846 (PMC13415438; doi:10.3390/pharmaceutics18070846)
Supplement: Supplementary file 1 [file pharmaceutics-18-00846-s001.zip › Table S18-S23. Haplotype frequency estimation EULAR response, LDA, Remission at 3 and 6 months Tofacitinib.pdf]

Table S18. Haplotype frequency estimation EULAR response at 3 months of Tofacitinib

JAK1 SNPs					EULAR Response			
**rs2230587**	**rs310241**	**rs2230588**	**rs10889504**	**rs2780815**	**Total**	**Satisfactory**	**Unsatisfactory**	**Cumulative frequency**
G	A	T	G	T	0.515	0.500	0.529	0.515
G	G	C	G	G	0.199	0.144	0.227	0.715
G	A	T	G	G	0.143	0.174	0.121	0.859
A	A	T	C	G	0.069	0.117	0.045	0.929
G	A	C	G	T	0.014	0	0.015	0.943
G	G	T	G	G	0.010	0.031	NA	0.953
A	A	T	G	G	0.010	NA	0.015	0.963
G	A	T	C	T	0.010	NA	0.015	0.973
G	A	T	C	G	0.010	0	0.015	0.983
A	G	T	C	G	0.010	NA	0.015	0.993
G	A	C	G	G	0.006	0.031	NA	1
G	G	T	G	T	0	0	NA	1
A	A	T	C	T	0	0	0	1
A	G	T	C	T	0	NA	0	1
JAK2 SNPs					EULAR Response			
**rs10119004**	**rs7857730**	**rs2274472**	**rs2230722**	**rs2230724**	**Total**	**Satisfactory**	**Unsatisfactory**	**Cumulative frequency**
G	G	C	C	G	0.320	0.181	0.381	0.320
A	T	T	C	A	0.205	0.191	0.239	0.526
A	T	T	T	A	0.135	0.052	0.138	0.661
G	G	T	C	G	0.085	0.044	0.106	0.747
A	G	C	C	G	0.040	0	0.041	0.788
G	G	T	T	G	0.038	0.034	0.042	0.827
A	T	C	T	A	0.035	0.090	0.031	0.863
A	T	C	C	A	0.030	0.073	NA	0.893
G	T	T	C	A	0.030	0.085	NA	0.924
G	T	C	C	G	0.030	0.088	0	0.954
A	G	T	C	G	0.014	0.093	0.003	0.968
G	T	T	C	G	0.012	0.029	NA	0.981
G	T	C	C	A	0.011	0.037	NA	0.992
A	T	T	C	G	0.007	0	NA	1
G	T	C	T	G	0	NA	0.015	1
A	G	C	T	G	0	0	NA	1
A	T	C	C	G	0	NA	NA	1
JAK3 SNPs					EULAR Response			
**rs3212780**	**rs3008**		**rs3212752**		**Total**	**Satisfactory**	**Unsatisfactory**	**Cumulative frequency**
G	G		T		0.394	0.352	0.386	0.394
G	A		T		0.292	0.323	0.310	0.687
A	G		T		0.132	0.235	0.083	0.820
A	A		T		0.120	NA	0.174	0.940
G	A		C		0.032	0	0	0.972
A	A		C		0.014	NA	0.045	0.987
A	G		C		0.012	NA	NA	1
G	G		C		0	0.088	NA	1

SNP: single nucleotide polymorphism; JAK: Janus kinase; NA: not available (frequency could not be estimated due to low counts); Total: overall haplotype frequency in the study population. EULAR: European Alliance of Associations for Rheumatology; Satisfactory/Unsatisfactory: clinical response categories according to EULAR criteria.

Table S19. Haplotype frequency estimation LDA at 3 months Tofacitinib.

JAK1 SNPs					LDA			
**rs2230587**	**rs310241**	**rs2230588**	**rs10889504**	**rs2780815**	**Total**	**LDA**	**No LDA**	**Cumulative frequency**
G	A	T	G	T	0.515	0.250	0.565	0.515
G	G	C	G	G	0.199	0.208	0.197	0.715
G	A	T	G	G	0.143	0.375	0.105	0.859
A	A	T	C	G	0.069	0	0.065	0.929
G	A	C	G	T	0.014	0.083	NA	0.943
G	G	T	G	G	0.010	NA	0.013	0.953
A	A	T	G	G	0.010	NA	0.013	0.963
G	A	T	C	T	0.010	NA	0.013	0.973

G	A	T	C	G	0.010	0	0.013	0.983
A	G	T	C	G	0.010	0	0.013	0.993
G	A	C	G	G	0.006	NA	NA	1
G	G	T	G	T	0	0	0	1
A	A	T	C	T	0	0.083	NA	1
A	G	T	C	T	0	NA	0	1
JAK2 SNPs					LDA			
**rs10119004**	**rs7857730**	**rs2274472**	**rs2230722**	**rs2230724**	**Total**	**LDA**	**No LDA**	**Cumulative frequency**
G	G	C	C	G	0.320	0.277	0.329	0.320
A	T	T	C	A	0.205	0.063	0.261	0.526
A	T	T	T	A	0.135	0.068	0.142	0.661
G	G	T	C	G	0.085	0.117	0.078	0.747
A	G	C	C	G	0.040	0	0.051	0.788
G	G	T	T	G	0.038	0.048	0.038	0.827
A	T	C	T	A	0.035	0.049	0.042	0.863
A	T	C	C	A	0.0308	0.106	NA	0.8938
G	T	T	C	A	0.030	0.073	0.013	0.924
G	T	C	C	G	0.030	0.052	0.024	0.954
A	G	T	C	G	0.014	0.056	0.002	0.968
G	T	T	C	G	0.012	NA	0.015	0.981
G	T	C	C	A	0.011	0.055	NA	0.992
A	T	T	C	G	0.007	0.031	NA	1
G	T	C	T	G	0	NA	0	1
A	G	C	T	G	0	NA	NA	1
A	T	C	C	G	0	0	NA	1
JAK3 SNPs					LDA			
**rs3212780**	**rs3008**		**rs3212752**		**Total**	**LDA**	**No LDA**	**Cumulative frequency**
G	G		T		0.394	0.333	0.379	0.394
G	A		T		0.292	0.250	0.337	0.687
A	G		T		0.132	0.333	0.094	0.820
A	A		T		0.120	0	0.136	0.940
G	A		C		0.032	0	0.020	0.972
A	A		C		0.014	NA	0.032	0.987
A	G		C		0.012	0	NA	1
G	G		C		0	0.083	0	1
SNP: single nucleotide polymorphism; JAK: Janus kinase; NA: not available (frequency could not be estimated due to low counts); Total: overall haplotype frequency in the study population; LDA: low disease activity								

**Table S20. Haplotype frequency estimation remission at 3 months of Tofacitinib**								
JAK1 SNPs					Remission			
**rs2230587**	**rs310241**	**rs2230588**	**rs10889504**	**rs2780815**	**Total**	**Remission**	**No remission**	**Cumulative frequency**
G	A	T	G	T	0.515	0.700	0.494	0.515
G	G	C	G	G	0.199	0.100	0.211	0.715
G	A	T	G	G	0.143	NA	0.160	0.859
A	A	T	C	G	0.069	0.100	0.066	0.929
G	A	C	G	T	0.014	0	0.016	0.943
G	G	T	G	G	0.010	0.100	NA	0.953
A	A	T	G	G	0.010	0	0.011	0.963
G	A	T	C	T	0.010	NA	0.011	0.973
G	A	T	C	G	0.010	NA	0.011	0.983
A	G	T	C	G	0.010	NA	0.011	0.993
G	A	C	G	G	0.006	NA	0.005	1
G	G	T	G	T	0	NA	NA	1
A	A	T	C	T	0	0	NA	1
A	G	T	C	T	0	NA	0	1
JAK2 SNPs					Remission			
**rs10119004**	**rs7857730**	**rs2274472**	**rs2230722**	**rs2230724**	**Total**	**Remission**	**No remission**	**Cumulative frequency**
G	G	C	C	G	0.320	NA	0.357	0.320
A	T	T	C	A	0.205	0.500	0.173	0.526
A	T	T	T	A	0.135	0.100	0.139	0.661
G	G	T	C	G	0.085	NA	0.098	0.747

A	G	C	C	G	0.040	NA	0.043	0.788
G	G	T	T	G	0.038	NA	0.041	0.827
A	T	C	T	A	0.035	0.100	0.030	0.863
A	T	C	C	A	0.030	0	0.032	0.893
G	T	T	C	A	0.030	0.100	0.021	0.924
G	T	C	C	G	0.030	0.100	0.023	0.954
A	G	T	C	G	0.014	NA	0.015	0.968
G	T	T	C	G	0.012	0.100	NA	0.981
G	T	C	C	A	0.011	NA	0.013	0.992
A	T	T	C	G	0.007	NA	0.010	1
G	T	C	T	G	0	0	NA	1
A	G	C	T	G	0	0	NA	1
A	T	C	C	G	0	0	0	1
JAK3 SNPs					Remission			
**rs3212780**	**rs3008**		**rs3212752**		**Total**	**Remission**	**No remission**	**Cumulative frequency**
G	G		T		0.394	0.200	0.420	0.394
G	A		T		0.292	0.600	0.258	0.687
A	G		T		0.132	0.100	0.121	0.820
A	A		T		0.120	NA	0.144	0.940
G	A		C		0.032	0	0.021	0.972
A	A		C		0.014	NA	0.020	0.987
A	G		C		0.012	NA	0.013	1
G	G		C		0	0.100	0	1
SNP: single nucleotide polymorphism; JAK: Janus kinase; NA: not available (frequency could not be estimated due to low counts); Total: overall haplotype frequency in the study population								

**Tabla S21. Haplotype frequency EULAR response at 6 months of Tofacitinib**								
JAK1 SNPs					**EULAR Response**			
**rs2230587**	**rs310241**	**rs2230588**	**rs10889504**	**rs2780815**	**Total**	**Satisfactory**	**Unsatisfactory**	**Cumulative frequency**
G	A	T	G	T	0.507	0.555	0.476	0.507
G	G	C	G	G	0.204	0.250	0.166	0.712
G	A	T	G	G	0.146	0.055	0.214	0.858
A	A	T	C	G	0.076	0.083	0.071	0.935
G	A	C	G	T	0.018	0	0.023	0.953
G	G	T	G	G	0.013	0.027	NA	0.966
A	A	T	G	G	0.012	NA	0.023	0.979
A	G	T	C	G	0.012	NA	0.023	0.992
G	A	C	G	G	0.007	0.027	NA	1
G	G	T	G	T	0	0	NA	1
A	A	T	C	T	0	0	0	1
A	G	T	C	T	0	NA	0	1
JAK2 SNPs					**EULAR Response**			
**rs10119004**	**rs7857730**	**rs2274472**	**rs2230722**	**rs2230724**	**Total**	**Satisfactory**	**Unsatisfactory**	**Cumulative frequency**
G	G	C	C	G	0.314	0.237	0.378	0.314
A	T	T	C	A	0.179	0.239	0.116	0.494
A	T	T	T	A	0.128	0.165	0.084	0.622
G	G	T	C	G	0.093	0.090	0.101	0.716
A	G	C	C	G	0.057	0.086	0	0.774
G	T	T	C	A	0.040	0.031	0.043	0.814
G	T	C	C	G	0.039	0.027	0.056	0.854
A	T	C	C	A	0.038	0.064	0.030	0.893
G	G	T	T	G	0.030	0.029	0.031	0.923
A	T	C	T	A	0.020	NA	0.050	0.944
G	T	T	C	G	0.016	0.027	NA	0.960
A	G	T	C	G	0.01	0	0.060	0.976
G	T	C	C	A	0.015	NA	0.031	0.992
A	T	T	C	G	0.007	NA	0.014	1
G	T	C	T	G	0	NA	NA	1
A	T	C	C	G	0	NA	NA	1
A	G	C	T	G	0	NA	0	1
G	G	C	T	G	0	NA	NA	1

JAK3 SNPs			EULAR Response			
**rs3212780**	**rs3008**	**rs3212752**	**Total**	**Satisfactory**	**Unsatisfactory**	**Cumulative frequency**
G	A	T	0.370	0.397	0.355	0.370
G	G	T	0.309	0.241	0.382	0.679
A	G	T	0.203	0.258	0.141	0.883
A	A	T	0.065	0.047	0.073	0.948
G	G	C	0.0385	0.055	0	0.987
A	A	C	0.012	NA	0.023	1
G	A	C	0	0	NA	1

SNP: single nucleotide polymorphism; JAK: Janus kinase; NA: not available (frequency could not be estimated due to low counts); Total: overall haplotype frequency in the study population. EULAR: European Alliance of Associations for Rheumatology; Satisfactory/Unsatisfactory: clinical response categories according to EULAR criteria.

Tabla S22. Haplotype frequency LDA at 6months of Tofacitinib

JAK1 SNPs					LDA			
**rs2230587**	**rs310241**	**rs2230588**	**rs10889504**	**rs2780815**	**Total**	**LDA**	**No LDA**	**Cumulative frequency**
G	A	T	G	T	0.507	0	0.546	0.507
G	G	C	G	G	0.204	0	0.171	0.712
G	A	T	G	G	0.146	0.500	0.140	0.858
A	A	T	C	G	0.076	NA	0.093	0.935
G	A	C	G	T	0.018	0.071	NA	0.953
G	G	T	G	G	0.013	NA	0.015	0.966
A	A	T	G	G	0.012	NA	0.0156	0.979
A	G	T	C	G	0.012	NA	0.015	0.992
G	A	C	G	G	0.007	0.071	NA	1
G	G	T	G	T	0	0	0	1
A	A	T	C	T	0	NA	0	1
A	G	T	C	T	0	NA	0	1
**JAK2 SNPs**					**LDA**			
**rs10119004**	**rs7857730**	**rs2274472**	**rs2230722**	**rs2230724**	**Total**	**LDA**	**No LDA**	**Cumulative frequency**
G	G	C	C	G	0.314	0.428	0.293	0.314
A	T	T	C	A	0.179	0.214	0.177	0.494
A	T	T	T	A	0.128	0.142	0.123	0.622
G	G	T	C	G	0.093	0	0.113	0.716
A	G	C	C	G	0.057	0	0.071	0.774
G	T	T	C	A	0.040	0.071	0.032	0.814
G	T	C	C	G	0.039	NA	0.045	0.854
A	T	C	C	A	0.038	0.071	0.027	0.893
G	G	T	T	G	0.030	NA	0.036	0.923
A	T	C	T	A	0.020	NA	0.027	0.944
G	T	T	C	G	0.016	NA	0.022	0.960
A	G	T	C	G	0.016	0.071	0	0.976
G	T	C	C	A	0.015	0	0.018	0.992
A	T	T	C	G	0.007	NA	0.01	1
G	T	C	T	G	0	NA	NA	1
A	T	C	C	G	0	NA	NA	1
A	G	C	T	G	0	NA	NA	1
G	G	C	T	G	0	0	NA	1
JAK3 SNPs					**LDA**			
**rs3212780**	**rs3008**	**rs3212752**			**Total**	**LDA**	**No LDA**	**Cumulative frequency**
G	A	T			0.370	0.428	0.369	0.370

G	G	T	0.309	0.142	0.333	0.679
A	G	T	0.203	0.428	0.166	0.883
A	A	T	0.065	0	0.067	0.948
G	G	C	0.038	NA	0.046	0.987
A	A	C	0.012	NA	0.015	1
G	A	C	0	NA	0	1
SNP: single nucleotide polymorphism; JAK: Janus kinase; NA: not available (frequency could not be estimated due to low counts); Total: overall haplotype frequency in the study population; LDA: low disease activity						

Table S23. Haplotype frequency estimation Remission at 6 months of Tofacitinib

JAK1 SNPs					Remission			
**rs2230587**	**rs310241**	**rs2230588**	**rs10889504**	**rs2780815**	**Total**	**Remission**	**No remission**	**Cumulative frequency**
G	A	T	G	T	0.507	0.590	0.467	0.507
G	G	C	G	G	0.204	0.227	0.196	0.712
G	A	T	G	G	0.146	NA	0.211	0.858
A	A	T	C	G	0.076	0.136	0.053	0.935
G	A	C	G	T	0.018	NA	0.032	0.953
G	G	T	G	G	0.013	0.045	NA	0.966
A	A	T	G	G	0.012	NA	0.017	0.979
A	G	T	C	G	0.012	NA	0.017	0.992
G	A	C	G	G	0.007	NA	0.003	1
G	G	T	G	T	0	NA	NA	1
A	A	T	C	T	0	NA	0	1
JAK2 SNPs					Remission			
**rs10119004**	**rs7857730**	**rs2274472**	**rs2230722**	**rs2230724**	**Total**	**Remission**	**No remission**	**Cumulative frequency**
G	G	C	C	G	0.314	0.084	0.421	0.314
A	T	T	C	A	0.179	0.357	0.118	0.494
A	T	T	T	A	0.128	0.181	0.080	0.622
G	G	T	C	G	0.093	0.045	0.099	0.716
A	G	C	C	G	0.057	0.097	0	0.774
G	T	T	C	A	0.040	0.052	0.0332	0.814
G	T	C	C	G	0.039	0.045	0.041	0.854
A	T	C	C	A	0.038	0	0.045	0.893
G	G	T	T	G	0.030	0.045	0.023	0.923
A	T	C	T	A	0.020	0.045	0.039	0.944
G	T	T	C	G	0.016	0.045	NA	0.960
A	G	T	C	G	0.016	NA	0.062	0.976
G	T	C	C	A	0.015	NA	0.023	0.992
A	T	T	C	G	0.007	NA	0.011	1
G	T	C	T	G	0	NA	NA	1
A	T	C	C	G	0	NA	NA	1
A	G	C	T	G	0	NA	0	1
G	G	C	T	G	0	NA	NA	1
JAK3 SNPs					Remission			
**rs3212780**	**rs3008**		**rs3212752**		**Total**	**Remission**	**No remission**	**Cumulative frequency**
G	A		T		0.370	0.454	0.330	0.370
G	G		T		0.309	0.272	0.347	0.679
A	G		T		0.203	0.181	0.187	0.883
A	A		T		0.065	NA	0.097	0.948
G	G		C		0.038	0.090	0	0.987
A	A		C		0.012	NA	0.017	1
G	A		C		0	0	0	1
SNP: single nucleotide polymorphism; JAK: Janus kinase; NA: not available (frequency could not be estimated due to low counts); Total: overall haplotype frequency in the study population								
