# Supplementary material for: Clinical and Pharmacogenetic Factors Associated with Response to JAK Inhibitors in Patients with Rheumatoid Arthritis: A Real-World Study of JAK1, JAK2, and JAK3 Gene Variants
Source: Pharmaceutics. 2026 Jul 11;18(7):846. doi: 10.3390/pharmaceutics18070846 (PMC13415438; doi:10.3390/pharmaceutics18070846)
Supplement: Supplementary file 1 [file pharmaceutics-18-00846-s001.zip › Table S2. Clinical effectiveness of JAK inhibitors for RA bio-naive patients.pdf]

Table S2. Clinical effectiveness of JAK inhibitors for RA bio-naïve patients at 3 and 6 months.

	Tofacitinib				Baricitinib				Filgotinib				Upadacitinib			
	Bio naïve patients															
**Response variable**	**3 months**		**6 months**		**3 months**		**6 months**		**3 months**		**6 months**		**3 months**		**6 months**	
	**N**	**%**	**N**	**%**	**N**	**%**	**N**	**%**	**N**	**%**	**N**	**%**	**N**	**%**	**N**	**%**
**EULAR response**	**4**		**4**		**4**		**4**		**-**				**3**		**6**	
Satisfactory	3	75	1	25	4	100	3	75	-				1	33.3	2	66.7
Unsatisfactory	1	25	3	75	0	0	1	25	-				2	66.7	1	33.3
**Remission (DAS28<2.6)**	1	25	1	25	4	100	3	75	-				0	0	1	33.3
**LDA (2.6≤ DAS28≤ 3.2)**	2	50	0	0	0	0	2	50	-				1	33.3	1	33.3

DAS28: disease activity score in 28 joints; EULAR: European League Against Rheumatism criteria; LDA: low-activity disease
There were no Bio naïve patients for filgotinib.
