# Supplementary material for: Clinical and Pharmacogenetic Factors Associated with Response to JAK Inhibitors in Patients with Rheumatoid Arthritis: A Real-World Study of JAK1, JAK2, and JAK3 Gene Variants
Source: Pharmaceutics. 2026 Jul 11;18(7):846. doi: 10.3390/pharmaceutics18070846 (PMC13415438; doi:10.3390/pharmaceutics18070846)
Supplement: Supplementary file 1 [file pharmaceutics-18-00846-s001.zip › Table S24-S29. Haplotype frequency estimation EULAR response, LDA, Remission at 3 and 6 months Upadacitinib.pdf]

| Table S24. Haplotype frequency estimation EULAR response at 3 months of Upadacitinib | | | | | | | | |
|---|---|---|---|---|---|---|---|---|
| *JAK1* SNPs | | | | | EULAR response | | | |
| **rs2230587** | **rs310241** | **rs2230588** | **rs10889504** | **rs2780815** | **Total** | **Satisfactory** | **Unsatisfactory** | **Cumulative frequency** |
| G | A | T | G | T | 0.582 | 0.550 | 0.596 | 0.582 |
| G | G | C | G | G | 0.194 | 0.100 | 0.230 | 0.776 |
| A | A | T | C | G | 0.096 | 0.150 | 0.076 | 0.873 |
| G | A | T | G | G | 0.028 | NA | 0.038 | 0.902 |
| A | G | T | G | G | 0.027 | 0.050 | 0.019 | 0.930 |
| G | A | T | C | T | 0.014 | NA | 0.019 | 0.944 |
| A | A | T | G | G | 0.014 | 0.050 | NA | 0.958 |
| G | G | C | C | G | 0.014 | NA | 0.019 | 0.972 |
| G | G | T | G | G | 0.013 | 0.050 | NA | 0.986 |
| G | A | T | C | G | 0.013 | 0.050 | NA | 1 |
| A | G | C | C | G | 0 | NA | NA | 1 |
| A | G | C | G | G | 0 | NA | NA | 1 |
| G | A | C | G | G | 0 | 0 | NA | 1 |
| *JAK2* SNPs | | | | | EULAR response | | | |
| **rs10119004** | **rs7857730** | **rs2274472** | **rs2230722** | **rs2230724** | **Total** | **Satisfactory** | **Unsatisfactory** | **Cumulative frequency** |
| G | G | C | C | G | 0.285 | 0 | 0.338 | 0.285 |
| A | T | T | C | A | 0.277 | 0.100 | 0.326 | 0.562 |
| G | G | T | C | G | 0.116 | 0.138 | 0.103 | 0.678 |
| A | T | C | T | A | 0.107 | NA | 0.142 | 0.786 |
| A | T | T | T | A | 0.085 | 0.150 | 0.050 | 0.871 |
| G | T | C | C | G | 0.036 | 0.100 | 0.019 | 0.907 |
| G | G | T | T | G | 0.028 | 0.250 | NA | 0.935 |
| G | T | T | C | G | 0.019 | 0.050 | NA | 0.955 |
| A | G | C | C | G | 0.015 | 0.062 | 0.019 | 0.971 |
| G | T | T | T | A | 0.015 | 0 | NA | 0.986 |
| A | G | T | T | G | 0.013 | NA | NA | 1 |
| T | T | C | T | G | 0 | 0 | NA | 1 |
| *JAK3* SNPs | | | | | EULAR response | | | |
| **Rs3212780** | **rs3008** | | **rs3212752** | | **Total** | **Satisfactory** | **Unsatisfactory** | **Cumulative frequency** |
| G | A | | T | | 0.415 | 0.371 | 0.432 | 0.417 |
| G | G | | T | | 0.304 | 0.279 | 0.317 | 0.722 |
| A | G | | T | | 0.112 | 0.121 | 0.105 | 0.834 |
| A | A | | T | | 0.109 | 0.179 | 0.086 | 0.944 |
| G | G | | C | | 0.041 | 0.050 | 0.038 | 0.986 |
| A | A | | C | | 0.013 | NA | 0.019 | 1 |
| G | A | | C | | 0 | 0 | 0 | 1 |

SNP: single nucleotide polymorphism; JAK: Janus kinase; NA: not available (frequency could not be estimated due to low counts); Total: overall haplotype frequency in the study population. EULAR: European Alliance of Associations for Rheumatology; Satisfactory/Unsatisfactory: clinical response categories according to EULAR criteria

| Table S25. Haplotype frequency estimation LDA at 3 months of Upadacitinib | | | | | | | | |
|---|---|---|---|---|---|---|---|---|
| *JAK1* SNPs | | | | | LDA | | | |
| **rs2230587** | **rs310241** | **rs2230588** | **rs10889504** | **rs2780815** | **Total** | **LDA** | **No LDA** | **Cumulative frequency** |
| G | A | T | G | T | 0.582 | 0.625 | 0.577 | 0.582 |
| G | G | C | G | G | 0.194 | NA | 0.218 | 0.776 |
| A | A | T | C | G | 0.096 | 0.125 | 0.093 | 0.873 |
| G | A | T | G | G | 0.025 | NA | 0.032 | 0.902 |
| A | G | T | G | G | 0.027 | 0.125 | 0.015 | 0.944 |
| G | A | T | C | T | 0.014 | NA | 0.016 | 0.944 |
| A | A | T | G | G | 0.014 | NA | 0.016 | 0.958 |
| G | G | C | C | G | 0.014 | NA | 0.015 | 0.972 |
| G | G | T | G | G | 0.013 | 0.125 | NA | 0.986 |
| G | A | T | C | G | 0.013 | NA | 0.014 | 1 |
| A | G | C | C | G | 0 | NA | 0 | 1 |
| A | G | C | G | G | 0 | NA | NA | 1 |
| G | A | C | G | G | 0 | NA | NA | 1 |
| *JAK2* SNPs | | | | | LDA | | | |
| **rs10119004** | **rs7857730** | **rs2274472** | **rs2230722** | **rs2230724** | **Total** | **LDA** | **No LDA** | **Cumulative frequency** |
| G | G | C | C | G | 0.285 | NA | 0.292 | 0.285 |
| A | T | T | C | A | 0.277 | NA | 0.305 | 0.562 |
| G | G | T | C | G | 0.116 | 0.375 | 0.114 | 0.678 |
| A | T | C | T | A | 0.107 | NA | 0.113 | 0.786 |
| A | T | T | T | A | 0.085 | NA | 0.097 | 0.871 |
| G | T | C | C | G | 0.036 | 0.125 | 0.022 | 0.907 |
| G | G | T | T | G | 0.028 | 0.125 | 0.015 | 0.935 |
| G | T | T | C | G | 0.019 | 0.125 | 0 | 0.955 |
| A | G | C | C | G | 0.015 | 0 | 0.016 | 0.971 |
| G | T | T | T | A | 0.015 | NA | 0.016 | 0.986 |
| A | G | T | T | G | 0.013 | NA | NA | 1 |
| T | T | C | T | G | 0 | NA | 0.008 | 1 |
| *JAK3* SNPs | | | | | LDA | | | |
| **rs3212780** | **rs3008** | | **rs3212752** | | **Total** | **LDA** | **No LDA** | **Cumulative frequency** |
| G | A | | T | | 0.417 | 0.250 | 0.422 | 0.417 |
| G | G | | T | | 0.304 | 0.375 | 0.311 | 0.722 |
| A | G | | T | | 0.112 | 0.25 | 0.094 | 0.834 |
| A | A | | T | | 0.109 | 0 | 0.124 | 0.944 |
| G | G | | C | | 0.041 | 0 | 0.031 | 0.986 |
| A | A | | C | | 0.013 | NA | 0.015 | 1 |
| G | A | | C | | 0 | 0.125 | 0 | 1 |

SNP: single nucleotide polymorphism; JAK: Janus kinase; NA: not available (frequency could not be estimated due to low counts); Total: overall haplotype frequency in the study population; LDA: low disease activity

| Table S26. Haplotype frequency Remission at 3 months of Upadacitinib | | | | | | | | |
|---|---|---|---|---|---|---|---|---|
| *JAK1* SNPs | | | | | Remission | | | |
| **rs2230587** | **rs310241** | **rs2230588** | **rs10889504** | **rs2780815** | **Total** | **Remission** | **No remission** | **Cumulative frequency** |
| G | A | T | G | T | 0.582 | 0.400 | 0.612 | 0.582 |
| G | G | C | G | G | 0.194 | 0.200 | 0.193 | 0.776 |
| A | A | T | C | G | 0.096 | 0.200 | 0.080 | 0.873 |
| G | A | T | G | G | 0.028 | NA | 0.032 | 0.902 |
| A | G | T | G | G | 0.027 | NA | 0.032 | 0.930 |
| G | A | T | C | T | 0.014 | NA | 0.016 | 0.944 |
| A | A | T | G | G | 0.014 | 0.100 | NA | 0.958 |
| G | G | C | C | G | 0.014 | NA | 0.016 | 0.972 |
| G | G | T | G | G | 0.013 | NA | 0.016 | 0.986 |

| | | | | | | | | |
|---|---|---|---|---|---|---|---|---|
| G | A | T | C | G | 0.013 | 0.100 | NA | 1 |
| A | G | C | C | G | 0 | NA | NA | 1 |
| A | G | C | G | G | 0 | NA | NA | 1 |
| G | A | C | G | G | 0 | NA | NA | 1 |
| *JAK2* SNPs | | | | | Remission | | | |
| **rs10119004** | **rs7857730** | **rs2274472** | **rs2230722** | **rs2230724** | **Total** | **Remission** | **No remission** | **Cumulative frequency** |
| G | G | C | C | G | 0.285 | 0 | 0.314 | 0.280 |
| A | T | T | C | A | 0.277 | 0.300 | 0.290 | 0.562 |
| G | G | T | C | G | 0.116 | 0.200 | 0.103 | 0.678 |
| A | T | C | T | A | 0.107 | 0 | 0.130 | 0.786 |
| A | T | T | T | A | 0.085 | 0.200 | 0.046 | 0.871 |
| G | T | C | C | G | 0.036 | NA | 0.037 | 0.907 |
| G | G | T | T | G | 0.028 | 0.100 | 0.017 | 0.935 |
| G | T | T | C | G | 0.019 | NA | 0.026 | 0.955 |
| A | G | C | C | G | 0.015 | NA | 0.017 | 0.971 |
| G | T | T | T | A | 0.015 | 0.100 | NA | 0.986 |
| A | G | T | T | G | 0.013 | NA | 0.015 | 1 |
| T | T | C | T | G | 0 | 0 | 0 | 1 |
| *JAK3* SNPs | | | | | Remission | | | |
| **rs3212780** | **rs3008** | | **rs3212752** | | **Total** | **Remission** | **No remission** | **Cumulative frequency** |
| G | A | | T | | 0.417 | 0.2 | 0.445 | 0.417 |
| G | G | | T | | 0.304 | 0.4 | 0.296 | 0.722 |
| A | G | | T | | 0.112 | 0 | 0.122 | 0.834 |
| A | A | | T | | 0.109 | 0.4 | 0.070 | 0.944 |
| G | G | | C | | 0.041 | NA | 0.048 | 0.986 |
| A | A | | C | | 0.013 | NA | 0.016 | 1 |
| G | A | | C | | 0 | NA | 0 | 1 |
| SNP: single nucleotide polymorphism; JAK: Janus kinase; NA: not available (frequency could not be estimated due to low counts); Total: overall haplotype frequency in the study population. | | | | | | | | |

**Table S27. Haplotype frequency estimation EULAR response at 6 months of Upadacitinib**

| *JAK1* SNPs | | | | | EULAR | | | |
|---|---|---|---|---|---|---|---|---|
| **rs2230587** | **rs310241** | **rs2230588** | **rs10889504** | **rs2780815** | **Total** | **Satisfactory** | **Unsatisfactory** | **Cumulative frequency** |
| G | A | T | G | T | 0.676 | 0.666 | 0.684 | 0.676 |
| G | G | C | G | G | 0.112 | 0.041 | 0.131 | 0.789 |
| A | A | T | C | G | 0.080 | 0.125 | 0.052 | 0.869 |
| G | A | T | G | G | 0.033 | NA | 0.052 | 0.902 |
| A | G | T | G | G | 0.032 | 0.083 | 0.026 | 0.935 |
| G | A | T | C | T | 0.017 | NA | 0.026 | 0.952 |
| A | A | T | G | G | 0.016 | 0 | NA | 0.968 |
| G | G | T | G | G | 0.016 | NA | 0.026 | 0.984 |
| G | A | T | C | G | 0.015 | 0.041 | NA | 1 |
| A | G | C | G | G | 0 | NA | NA | 1 |
| G | A | C | G | G | 0 | 0.041 | NA | 1 |
| *JAK2* SNPs | | | | | EULAR response | | | |
| **rs10119004** | **rs7857730** | **rs2274472** | **rs2230722** | **rs2230724** | **Total** | **Satisfactory** | **Unsatisfactory** | **Cumulative frequency** |
| G | G | C | C | G | 0.309 | NA | 0.362 | 0.309 |
| A | T | T | C | A | 0.274 | 0.083 | 0.330 | 0.584 |
| A | T | T | T | A | 0.111 | 0.125 | 0.051 | 0.695 |
| G | G | T | C | G | 0.107 | 0.162 | 0.085 | 0.803 |
| A | T | C | T | A | 0.063 | NA | 0.065 | 0.867 |
| G | G | T | T | G | 0.033 | 0.291 | NA | 0.900 |
| G | T | C | C | G | 0.026 | NA | 0.031 | 0.926 |
| G | T | T | C | G | 0.020 | NA | 0.032 | 0.947 |
| A | G | C | C | G | 0.018 | 0.045 | 0.026 | 0.966 |
| G | T | T | T | A | 0.017 | 0 | NA | 0.983 |
| A | G | T | T | G | 0.015 | NA | NA | 0.999 |
| G | T | C | T | G | 6e-04 | NA | 0.015 | 1 |
| *JAK3* SNPs | | | | | EULAR response | | | |
| **rs3212780** | **rs3008** | | **rs3212752** | | **Total** | **Satisfactory** | **Unsatisfactory** | **Cumulative frequency** |
| G | A | | T | | 0.378 | 0.291 | 0.344 | 0.378 |
| G | G | | T | | 0.330 | 0.37 | 0.381 | 0.709 |
| A | A | | T | | 0.121 | 0.250 | 0.103 | 0.830 |
| A | G | | T | | 0.104 | 0 | 0.118 | 0.935 |
| G | G | | C | | 0.048 | 0.083 | 0 | 0.983 |
| A | A | | C | | 0.016 | NA | 0.015 | 1 |
| G | A | | C | | 0 | 0 | 0.037 | 1 |
| SNP: single nucleotide polymorphism; JAK: Janus kinase; NA: not available (frequency could not be estimated due to low counts); Total: overall haplotype frequency in the study population. EULAR: European Alliance of Associations for Rheumatology; Satisfactory/Unsatisfactory: clinical response categories according to EULAR criteria | | | | | | | | |

**Table S28. Haplotype frequency estimation LDA at 6 months of Upadacitinib**

| *JAK1* SNPs | | | | | LDA | | | |
|---|---|---|---|---|---|---|---|---|
| **rs2230587** | **rs310241** | **rs2230588** | **rs10889504** | **rs2780815** | **Total** | **LDA** | **No LDA** | **Cumulative frequency** |
| G | A | T | G | T | 0.676 | 0.688 | 0.500 | 0.676 |
| G | G | C | G | G | 0.112 | 0.120 | NA | 0.789 |
| A | A | T | C | G | 0.080 | 0.085 | NA | 0.869 |
| G | A | T | G | G | 0.033 | 0.035 | NA | 0.902 |
| A | G | T | G | G | 0.032 | 0.017 | 0.250 | 0.935 |
| G | A | T | C | T | 0.017 | 0.018 | NA | 0.952 |
| A | A | T | G | G | 0.016 | 0.017 | NA | 0.968 |
| G | G | T | G | G | 0.016 | NA | 0.250 | 0.984 |
| G | A | T | C | G | 0.015 | 0.016 | NA | 1 |
| A | G | C | G | G | 0 | 0 | NA | 1 |
| G | A | C | G | G | 0 | NA | NA | 1 |
| *JAK2* SNPs | | | | | LDA | | | |
| **rs10119004** | **rs7857730** | **rs2274472** | **Rs2230722** | **rs2230724** | **Total** | **LDA** | **No LDA** | **Cumulative frequency** |
| G | G | C | C | G | 0.309 | 0.314 | 0.250 | 0.309 |
| A | T | T | C | A | 0.274 | 0.310 | NA | 0.584 |
| A | T | T | T | A | 0.111 | 0.107 | NA | 0.695 |
| G | G | T | C | G | 0.107 | 0.115 | NA | 0.803 |
| A | T | C | T | A | 0.063 | 0.064 | NA | 0.867 |
| G | G | T | T | G | 0.033 | 0.034 | NA | 0.900 |
| G | T | C | C | G | 0.026 | 0 | 0.250 | 0.926 |
| G | T | T | C | G | 0.020 | NA | 0.250 | 0.947 |
| A | G | C | C | G | 0.018 | 0.017 | NA | 0.966 |
| G | T | T | T | A | 0.017 | 0.017 | NA | 0.983 |

| | | | | | | | | |
|---|---|---|---|---|---|---|---|---|
| A | G | T | T | G | 0.015 | NA | 0.250 | 0.999 |
| G | T | C | T | G | 6e-04 | 0.017 | NA | 1 |
| *JAK3* SNPs | | | | | LDA | | | |
| **rs3212780** | **rs3008** | | **rs3212752** | | **Total** | **LDA** | **No LDA** | **Cumulative frequency** |
| G | A | | T | | 0.378 | 0.392 | 0.250 | 0.378 |
| G | G | | T | | 0.330 | 0.332 | 0.250 | 0.709 |
| A | A | | T | | 0.121 | 0.125 | 0 | 0.830 |
| A | G | | T | | 0.104 | 0.081 | 0.500 | 0.935 |
| G | G | | C | | 0.048 | 0.051 | NA | 0.983 |
| A | A | | C | | 0.016 | 0.017 | NA | 1 |
| G | A | | C | | 0 | 0 | NA | 1 |
| SNP: single nucleotide polymorphism; JAK: Janus kinase; NA: not available (frequency could not be estimated due to low counts); Total: overall haplotype frequency in the study population; LDA: low disease activity; No LDA: patients not achieving low disease activity | | | | | | | | |

| **Table S29. Haplotype frequency estimation Remission at 6 months of Upadacitinib** | | | | | | | | |
|---|---|---|---|---|---|---|---|---|
| *JAK1* SNPs | | | | | **Remission** | | | |
| **rs2230587** | **rs310241** | **rs2230588** | **rs10889504** | **rs2780815** | **Total** | **Remission** | **No remission** | **Cumulative frequency** |
| G | A | T | G | T | 0.676 | 0.690 | 0.65 | 0.676 |
| G | G | C | G | G | 0.112 | 0.119 | 0.1 | 0.789 |
| A | A | T | C | G | 0.080 | 0.047 | 0.15 | 0.869 |
| G | A | T | G | G | 0.033 | 0.047 | NA | 0.902 |
| A | G | T | G | G | 0.032 | 0.047 | NA | 0.935 |
| G | A | T | C | T | 0.017 | 0.023 | NA | 0.952 |
| A | A | T | G | G | 0.016 | NA | 0.05 | 0.968 |
| G | G | T | G | G | 0.016 | 0.023 | NA | 0.984 |
| G | A | T | C | G | 0.015 | NA | 0.05 | 1 |
| A | G | C | G | G | 0 | NA | NA | 1 |
| G | A | C | G | G | 0 | NA | NA | 1 |
| *JAK2* SNPs | | | | | **Remission** | | | |
| **rs10119004** | **rs7857730** | **rs2274472** | **rs2230722** | **rs2230724** | **Total** | **Remission** | **No remission** | **Cumulative frequency** |
| G | G | C | C | G | 0.309 | 0.314 | 0.250 | 0.309 |
| A | T | T | C | A | 0.274 | 0.310 | NA | 0.584 |
| A | T | T | T | A | 0.111 | 0.107 | NA | 0.695 |
| G | G | T | C | G | 0.107 | 0.115 | NA | 0.803 |
| A | T | C | T | A | 0.063 | 0.064 | NA | 0.867 |
| G | G | T | T | G | 0.033 | 0.034 | NA | 0.900 |
| G | T | C | C | G | 0.026 | 0 | 0.250 | 0.926 |
| G | T | T | C | G | 0.020 | NA | 0.250 | 0.947 |
| A | G | C | C | G | 0.018 | 0.017 | NA | 0.966 |
| G | T | T | T | A | 0.017 | 0.017 | NA | 0.983 |
| A | G | T | T | G | 0.015 | NA | 0.250 | 0.999 |
| G | T | C | T | G | 6e-04 | 0.017 | NA | 1 |
| *JAK3* SNPs | | | | | **Remission** | | | |
| **rs3212780** | **rs3008** | | **rs3212752** | | **Total** | **Remission** | **No remission** | **Cumulative frequency** |
| G | A | | T | | 0.378 | 0.330 | 0.350 | 0.378 |
| G | G | | T | | 0.330 | 0.369 | 0.350 | 0.709 |
| A | A | | T | | 0.121 | 0.122 | 0.200 | 0.830 |
| A | G | | T | | 0.104 | 0.130 | 0 | 0.935 |
| G | G | | C | | 0.048 | 0 | 0.100 | 0.983 |
| A | A | | C | | 0.016 | 0.009 | NA | 1 |
| G | A | | C | | 0 | 0.037 | 0 | 1 |
| SNP: single nucleotide polymorphism; JAK: Janus kinase; NA: not available (frequency could not be estimated due to low counts); Total: overall haplotype frequency in the study population. | | | | | | | | |
