# Supplementary material for: Clinical and Pharmacogenetic Factors Associated with Response to JAK Inhibitors in Patients with Rheumatoid Arthritis: A Real-World Study of JAK1, JAK2, and JAK3 Gene Variants
Source: Pharmaceutics. 2026 Jul 11;18(7):846. doi: 10.3390/pharmaceutics18070846 (PMC13415438; doi:10.3390/pharmaceutics18070846)
Supplement: Supplementary file 1 [file pharmaceutics-18-00846-s001.zip › Table S3. Hardy Weinberg equilibrium.pdf]

Table S3. Hardy–Weinberg Equilibrium Results.

Drug	Chr	SNP	Minor allele	Major allele	Genotype counts	Observed HET	Expected HET	p-value
**Tofacitinib**	1	rs2230587	A	G	0/9/41	0.180	0.163	1
		rs310241	G	A	2/18/30	0.360	0.343	1
		rs2230588	C	T	3/16/31	0.320	0.343	0.679
		rs10889504	C	G	0/10/40	0.200	0.180	1
		rs2780815	G	T	8/30/12	0.600	0.496	0.252
	9	rs10119004	A	G	13/21/16	0.420	0.498	0.267
		rs7857730	G	T	15/20/15	0.400	0.500	0.163
		rs2274472	C	T	10/27/13	0.540	0.498	0.776
		rs2230722	T	C	3/15/32	0.300	0.331	0.424
		rs2230724	G	A	13/19/18	0.380	0.495	0.096
	19	rs3212780	A	G	1/26/23	0.520	0.403	0.075
		rs3008	A	G	10/26/14	0.520	0.496	1
		rs3212752	C	T	0/6/44	0.120	0.112	1
**Baricitinib**	1	rs2230587	A	G	1/13/30	0.295	0.282	1
		rs310241	G	A	5/14/25	0.318	0.396	0.248
		rs2230588	C	T	4/15/25	0.340	0.386	0.442
		rs10889504	C	G	1/11/32	0.250	0.251	1
		rs2780815	G	T	11/18/15	0.409	0.495	0.239
	9	rs10119004	A	G	12/17/15	0.386	0.497	0.139
		rs7857730	G	T	8/18/18	0.409	0.474	0.354
		rs2274472	C	T	3/24/17	0.545	0.449	0.310
		rs2230722	T	C	2/15/27	0.340	0.338	1
		rs2230724	G	A	10/17/17	0.386	0.487	0.214
	19	rs3212780	A	G	5/22/17	0.500	0.462	0.749
		rs3008	A	G	8/20/16	0.454	0.483	0.756
		rs3212752	C	T	0/7/37	0.159	0.146	1
**Filgotinib**	1	rs2230587	A	G	0/5/15	0.250	0.218	1
		rs310241	G	A	1/6/13	0.300	0.320	1
		rs2230588	C	T	1/6/13	0.300	0.320	1
		rs10889504	C	G	0/3/17	0.150	0.138	1
		rs2780815	G	T	4/8/8	0.400	0.480	0.638
	9	rs10119004	A	G	5/9/6	0.450	0.498	0.669
		rs7857730	G	T	5/8/7	0.400	0.495	0.394
		rs2274472	C	T	3/12/5	0.600	0.495	0.649
		rs2230722	T	C	3/6/11	0.300	0.420	0.280
		rs2230724	G	A	6/7/7	0.350	0.498	0.197
	19	rs3212780	A	G	2/9/9	0.450	0.438	1
		rs3008	A	G	2/12/6	0.600	0.480	0.384
		rs3212752	C	T	0/1/19	0.050	0.048	1
**Upadacitinib**	1	rs2230587	A	G	1/8/27	0.222	0.239	0.514
		rs310241	G	A	3/12/21	0.333	0.375	0.653
		rs2230588	C	T	3/9/24	0.250	0.329	0.145
		rs10889504	C	G	2/6/28	0.166	0.239	0.110
		rs2780815	G	T	7/15/14	0.416	0.481	0.490
	9	rs10119004	A	G	10/16/10	0.444	0.500	0.515
		rs7857730	G	T	9/15/12	0.416	0.496	0.331
		rs2274472	C	T	5/22/9	0.611	0.493	0.307
		rs2230722	T	C	2/14/20	0.388	0.375	1
		rs2230724	G	A	10/15/11	0.416	0.499	0.329
	19	rs3212780	A	G	0/17/19	0.472	0.360	0.155
		rs3008	A	G	8/17/11	0.472	0.496	0.746
		rs3212752	C	T	0/4/32	0.111	0.104	1

Chr: chromosome; SNP: single-nucleotide polymorphism; HET: heterozygosity
