# Supplementary material for: Clinical and Pharmacogenetic Factors Associated with Response to JAK Inhibitors in Patients with Rheumatoid Arthritis: A Real-World Study of JAK1, JAK2, and JAK3 Gene Variants
Source: Pharmaceutics. 2026 Jul 11;18(7):846. doi: 10.3390/pharmaceutics18070846 (PMC13415438; doi:10.3390/pharmaceutics18070846)
Supplement: Supplementary file 1 [file pharmaceutics-18-00846-s001.zip › Table S30-S35. Predictors of EULAR response, LDA and remission at 3 and 6 months in RA patients treated with tofacitinib (Bivariate analyisis).pdf]

Table S30. Tofacitinib EULAR response bivariate demographic and clinical analyses

Clinical variables	3 months						6 months					
	N	EULAR response		p-value	OR	CI95%	N	EULAR response		p-value	OR	CI95%
		Satisfactory	Unsatisfactory					Satisfactory	Unsatisfactory			
**Sex**												
Woman	39	15 (38.5)	24 (61.5)	0.291*	-	-	34	15 (44.1)	19 (55.9)	0.640*	-	-
Man	11	2 (18.2)	9 (81.8)				5	3 (60)	2 (40)			
**Smoking**												
Smoker	6	2 (33.3)	4 (66.7)	0.460*	-	-	5	1 (20)	4 (80)	0.320*	-	-
Exsmoker	14	3 (21.4)	11 (78.6)				13	5 (38.5)	8 (61.5)			
No smoker	30	12 (40)	18 (60)				21	12 (57.1)	9 (42.9)			
Age at Dx	50	39.9±14.1	40.2±12.4	0.937	-	-	39	44.5 [32.2-48.7]	44 [32-49]	0.696	-	-
Years with RA	50	11[7-19]	15 [10-20]	0.352	-	-	39	10.5 [8-20]	15 [9-19]	0.934	-	-
Years from Dx till JAK inhibitor treatment	50	6 [3-12]	13 [6-17]	0.145	-	-	39	10.5 [8-20]	15 [9-19]	0.934	-	-
JAK inhibitor start age	50	5 [2-6]	5 [4-7]	0.304	-	-	39	51.5 [43.7-62.5]	56 [49- 61]	0.691	-	-
Treatment duration with JAK inhibitor (Months)	50	13.3 [12.1-29.3]	18.6 [5.7-41.1]	0.482	-	-	39	25.6 [12.3-50.6]	17.5 [12.5 42.5]	0.844	-	-
**Biologic-naïve**												
Yes	4	3 (75)	1 (25)	0.100*	-	-	4	1 (25)	3 (75)	0.600*	-	-
No	46	14 (30.4)	32 (69.6)				35	17 (48.6)	18 (51.4)			
Number of previous BTs	50	2 [1-2]	2 [2-3]	0.338	-	-	39	2 [1.25-2]	2 [1-4]	0.867	-	-
Previous BTs duration (months)	50	69.9 [36.4 -140.1]	69.9 [31.37- 135.1]	0.862	-	-		56.2 [41.3-136]	76.9 [40.2-133.6]	0.538	-	-
**BTs cause of suspensión**												
Primary failure	12	2 (16.7)	10 (83.3)	0.100	-	-	8	5 (62.5)	3 (37.5)	0.369	-	-
Secondary failure	27	7 (25.9)	20 (74.1)	0.140	-	-	20	8 (40)	12 (60)	0.314	-	-
Adverse events	7	5 (71.4)	2 (28.6)	0.020	8.33	[1.52-65.60]	7	4 (57.1)	3 (42.9)	0.690	-	-
Toxicity	-	-	-	-	-	-	-	-	-	-	-	-
Others	-	-	-	-	-	-	-	-	-	-	-	-
**Baseline RF (Cualitative)**												
Pos	37	10 (27)	27 (73)	0.098*	3.15	[0.85-12.15]	31	12 (38.7)	19 (61.3)	0.110*	-	-
Neg	13	7 (53.8)	6 (46.2)		1		8	6 (75)	2 (25)			
**Baseline ACPA**												
Pos	40	14 (35)	26 (65)	1*	-	-	31	15 (48.4)	16 (51.6)	0.700*	-	-
Neg	10	3 (30)	7 (70)				8	3 (37.5)	5 (62.5)			
**Baseline CCI**												
Absence	21	9(42.9)	12 (57.1)	0.550*	-	-	19	10 (52.6)	9 (47.4)	0.410*	-	-
Low	14	3 (21.4)	11 (78.6)				8	2 (25)	6 (75)			
High	14	5 (35.7)	9 (64.3)				12	6 (50)	6 (50)			
BMI	50	30.1 ± 6.1	28.6 ± 5.8	0.379	-	-	39	28.1 [24.9-31.2]	29.94 [26.1-34.5]	0.263	-	-
**JAK inhibitor dose change**												
no	44	14 (31.8)	30 (68.2)	0.390*	-	-	33	12 (36.4)	21 (63.6)	0.005*	4×10^{-9}	[NA - 1.38×10^{102}]
yes	6	3 (50)	3 (50)				6	6 (100)	0 (0)			
**JAK inhibitor suspensión**												
no	15	6 (40)	9 (60)	0.740*	-	-	14	10 (71.4)	4 (28.6)	0.023*	5.31	[1.34 -24.64]
yes	35	11 (31.4)	24 (68.6)				25	8 (32)	17 (68)			
**JAK inhibitor cause of suspension**												
Primary failure	8	0 (0)	8 (100)	0.023*	1.27×10^{-8}	[NA- NA]	4	0 (0)	4 (100)	0.062*	-	-
Secondary failure	10	6 (60)	4 (40)		6	[0.6 -143.4]	9	2 (22.2)	7 (77.8)			
Adverse events	5	1 (20)	4 (80)		1	1	9	6 (66.7)	3 (33.3)			
Others	2	0 (0)	2 (100)		1.27×10^{-8}	[NA - NA]	1	0 (0)	1 (100)			
**BT after JAK inhibitor treatment**												
no	22	11 (50)	11 (50)	0.040*	0.272	[0.07-0.90]	20	7 (35)	13 (65)	0.024*	-	-
Yes	28	22 (78.6)	6 (21.4)				19	14 (73.7)	5 (26.3)			
**Adverse events to JAK inhibitor**												
Yes	30	10 (33.3)	20 (66.7)	1*	-	-	23	11 (47.8)	12 (52.2)	1*	-	-
no	20	7 (35)	13 (65)				16	7 (43.8)	9 (56.2)			
**Concomitant DMARDs**												
MTX	11	5 (45.5)	6 (54.5)	0.620*	-	-	8	3 (37.5)	5 (62.5)	0.528*	-	-
HXQ	2	0	2 (100)				2	0 (0)	2 (100)			
SSZ	0	0	0				-	-	-			

**LFN**	2	0	2 (100)				1	1 (100)	-			
**None**	35	12 (34.3)	23 (65.7)				28	14 (50)	14 (50)			
**Concomitant statins**												
**no**	34	12 (35.3)	22 (64.7)	1*	-	-	27	12 (44.4)	15 (55.6)	0.740*	-	-
**yes**	16	5 (31.2)	11 (68.8)				12	6 (50)	6 (50)			
**Concomitant GC**												
**no**	15	8 (53.3)	7 (46.7)	0.100*	-	-	14	8 (57.1)	6 (42.9)	0.330*	-	-
**yes**	35	9 (25.7)	26 (74.3)				25	10 (40)	15 (60)			
**Concomitant vitamin D**												
**No**	23	9 (39.1)	14 (60.9)	0.550*	-	-	18	9 (50)	9 (50)	0.750*	-	-
**Yes**	27	8 (29.6)	19 (70.4)				21	9 (42.9)	12 (57.1)			
**Baseline DAS28**	50	4.4 ± 1.5	4.6 ± 0.8	0.997	-	-	39	2.5 [2.12-2.67]	3.9 [3.7-4.6]	0.997	-	-
**Baseline TJC**		1 [0-2]	4 [2-8]	<0.001	0.59	[0.38-0.80]	39	0.5 [0-1]	4 [3-6]	0.003	0.40	[0.20- 0.68]
**Baseline SJC**		0 [0-1]	1 [0-3]	0.201	-	-	39	0 [0-0]	1 [1-5]	0.009	0.20	[0.05-0.55]
**Baseline PVAS**		3.70 ± 2.22	5.36 ± 2.34	0.026	0.72	[0.53-0.94]	39	2 [2-4.5]	5 [4-7]	0.014	0.64	[0.43 -0.88]
**Baseline MVAS**		2 [1-3]	5 [2-6]	0.002*	0.57	[0.38- 0.79]	39	1.5 [1-2.7]	4 [3-6]	0.001	0.46	[0.26 -0.70]
**Baseline CRP**		0.9 [0.3-3]	4.3 [1.05-8.1]	0.131	-	-	39	1 [0.5-4.6]	1.3 [0.4-3]	0.363	-	-
**Baseline ESR**		18 [10-34]	23 [13-44]	0.373	-	-	39	14.5 [25.75-9]	31 [22-37]	0.325	-	-
**Baseline RF (Quantitative)**		22 [7.3-90]	62 [18-160]	0.812	-	-	39	49 [9.75-69.5]	65 [23-114]	0.391	-	-
**Baseline TC**		190 [171-213]	215 [191-242]	0.165	-	-	39	191.5 [178-215.25]	210 [183-222]	0.145	-	-
**Baseline LDL**		102 [90-124]	139 [111-153]	0.156	-	-	39	99.5 [86.7-119.5]	120 [100-135]	0.030*	0.973	[0.94-.99]
**Baseline TG**		105 [58- 129]	188 [127- 299]	0.023*	0.98	[0.97- 0.99]	39	113.5 [64.2 -152.7]	103.0 [80-147]	0.773	-	-

EULAR: European League Against Rheumatism; TJC: tender joints count; SJC: swallowed joint count; PVAS: patient visual analogue scale; MVAS: physician visual analogue scale; RF: rheumatoid factor; ACPA: anti-citrullinated protein antibodies; ESR: erythrocyte sedimentation rate; CRP: C-reactive protein; TC: total cholesterol; LDL: low-density lipoprotein; TG: triglycerides; BMI: body mass index; CCI: Charlson Comorbidity Index; JAK inhibitor: Janus kinase inhibitor; BTs: biologic therapies; GC: glucocorticoids; DMARDs: disease-modifying antirheumatic drugs; MTX: methotrexate; HXQ:, hydroxychloroquine; LFN, leflunomide; SSZ: sulfasalazine; OR, odds ratio; CI, confidence interval; NA: not available (indicates non-estimable values due to sparse data or quasi-complete separation); *: p value for Fisher's Exact Test

Table S31. Tofacitinib EULAR response bivariate genetic analyses

		3 months						6 meses				
**SNPs**	**Genotipe**	**N**	**EULAR response**		**p-value**	**OR**	**CI95%**	**N**	**EULAR response**		**p-value**	**OR**	**CI95%**
			**Satisfactory**	**Unsatisfactory**					**Satisfactory**	**Unsatisfactory**			
JAK1													
**rs2230587**	GG	41	13 (31.7)	28 (68.3)	0.467*	-	-	31	15 (48.4)	16 (51.6)	0.702*	-	-
	AA	0	0	0				0	0	0			
	AG	9	4 (44.4)	5 (55.6)				8	3 (37.5)	5 (62.5)			
	A	9	4(44.4)	5 (55.6)	0.467*	-	-	8	3 (37.5)	5 (62.5)	0.702*	-	-
	G	-	-	-	-	-	-	-	-	-	-	-	-
**rs310241**	AA	30	11 (36.7)	19 (63.3)	0.886*	-	-	23	9 (39.1)	14 (60.9)	0.657*	-	-
	AG	18	6 (33.3)	12 (66.7)				14	8 (57.1)	6 (42.9)			
	GG	2	0 (0)	2 (100)				2	1 (50)	1 (50)			
	A	48	17 (35.4)	31 (64.6)	0.542*	-	-	37	17 (45.9)	20 (54.1)	1*	-	-
	G	20	6 (30)	14 (70)	0.763*	-	-	7	9 (56.2)	7 (43.8)	0.342*	-	-
**rs2230588**	CC	3	0 (0)	3 (100)	0.630*	-	-	3	1 (33.3)	2 (66.7)	0.251*	-	-
	CT	16	6 (37.5)	10 (62.5)				12	8 (66.7)	4 (33.3)			
	TT	31	11 (35.5)	20 (64.5)				9	9 (37.5)	15 (62.5)			
	T	47	17 (36.2)	30 (63.8)	0.542*	-	-	36	17 (47.2)	19 (52.8)	1	-	-
	C	19	6 (41.6)	13 (68.4)	1*			15	9 (60)	6 (40)	0.202*	-	-
**rs10889504**	GG	40	13 (32.5)	27 (67.5)	0.717*	-	-	32	15 (46.9)	17 (53.1)	1*	-	-
	CC	0	0	0				0	0	0			
	CG	10	4 (40)	6 (60)				7	3 (42.9)	4 (57.1)			
	C	10	4 (40)	6 (60)	0.717*	-	-	7	3 (46.9)	4 (57.1)	1*	-	-
	G	-	-	-	-	-	-	-	-	-	-	-	-
**rs2780815**	GG	8	3 (37.5)	5 (62.5)	0.838*	-	-	7	2 (28.6)	5 (71.4)	0.557*	-	-
	GT	30	11 (36.7)	19 (63.3)				23	12 (52.2)	11 (47.8)			
	TT	12	3 (25)	9 (75)				9	4 (44.4)	5 (55.6)			
	T	42	14 (33.3)	28 (66.7)	1*	-	-	32	16 (50)	16 (50)	0.417*	-	-
	G	38	14 (36.8)	24 (63.2)	0.510*	-	-	30	1 (46.7)	16 (53.3)	1*	-	-
JAK2													
**rs10119004**	AA	13	5 (38.5)	8 (61.5)	0.932*	-	-	9	6 (66.7)	3 (33.3)	0.249*	-	-
	AG	21	7 (33.3)	14 (66.7)				17	8 (47.1)	9 (52.9)			
	GG	16	5 (31.2)	11 (68.8)				13	4 (30.8)	9 (69.2)			
	A	34	12 (35.3)	22 (64.7)	1*	-	-	26	12 (53.8)	14 (46.2)	0.307*	-	-
	G	37	12 (32.4)	25 (67.6)	0.741*	-	-	30	40 (13.8)	60 (16.1)	0.255*	-	-

**rs7857730**	GG	15	2 (13.3)	13 (86.7)				12	5 (41.7)	7 (58.3)			
	TT	15	7 (46.7)	8 (53.3)	0.103*	-	-	11	7 (63.3)	4 (36.3)	0.429*	-	-
	GT	20	8 (40)	12 (60)				16	6 (37.5)	10 (62.5)			
	G	35	10 (28.6)	25 (71.4)	0.328*	-	-	28	11 (39.3)	17 (60.7)	0.2849*	-	-
	T	35	15 (42.9)	20 (57.1)	0.045*	0.20	[0.02-0.89]	27	13 (48.1)	14 (51.9)	0.742*	-	-
**rs2274472**	CC	10	3 (30)	7 (70)				7	3 (42.9)	4 (57.1)			
	TT	13	4 (30.8)	9 (69.2)	1*	-	-	24	9 (37.5)	15 (62.5)	0.227*	-	-
	CT	27	10 (37)	17 (63)				8	6 (75)	2 (25)			
	C	37	13 (35.1)	24 (64.9)	1*	-	-	31	12 (38.7)	19 (61.3)	0.112*	-	-
	T	40	14 (35)	26 (65)	1*	-	-	32	15 (46.9)	17 (53.1)	1*	-	-
**rs2230722**	CC	32	11 (34.4)	21 (65.6)				26	12 (46.2)	14 (53.8)			
	TT	3	0 (0)	3 (100)	0.620*	-	-	1	1 (100)	0 (0)	0.724*	-	-
	CT	9	6 (40)	9 (60)				12	5 (41.7)	7 (58.3)			
	C	47	17 (36.2)	30 (63.8)	0.542*	-	-	38	17 (44.7)	21 (55.3)	0.461*	-	-
	T	18	6 (33.3)	12 (66.7)	1*	-	-	13	6 (46.2)	7 (53.8)	1*	-	-
**rs2230724**	AA	13	6 (46.2)	7 (53.8)				9	6 (66.7)	3 (33.3)			
	AG	19	6 (31.6)	13 (68.4)	0.564*	-	-	15	6 (40)	9 (60)	0.466*	-	
	GG	18	5 (27.8)	13 (72.2)				15	6 (40)	9 (60)			
	A	32	12 (37.5)	20 (62.5)	0.548*	-	-	24	12 (50)	12 (50)	0.742*	-	
	G	37	11 (29.7)	26 (70.3)	0.321*	-	-	30	12 (40)	18 (60)	0.255*	-	
JAK3													
**rs3212780**	AA	1	0 (0)	1 (100)				0	0	0			
	AG	26	8 (30.8)	18 (69.2)	0.712*	-	-	22	11 (50)	11 (50)	0.747*	-	-
	GG	23	9 (39.1)	14 (60.9)				17	7 (41.2)	10 (58.8)			
	A	27	8 (29.6)	19 (70.4)	0.556*	-	-	22	11 (50)	11 (50)	0.747*	-	-
	G	49	17 (34.7)	32 (65.3)	1*	-	-	-	-	-	-	-	-
**rs3008**	AA	10	2 (20)	8 (80)				8	3 (37.5)	5 (62.5)			
	AG	26	7 (26.9)	19 (73.1)	0.090*	-	-	19	10 (52.6)	9 (47.4)	0.762*	-	-
	GG	14	8 (57.1)	6 (42.9)				12	5 (41.7)	7 (58.3)			
	A	36	9 (25)	27 (75)	0.047*	0.25	[0.06- 0.90]	27	13 (48.1)	14 (51.9)	0.742*	-	-
	G	40	15 (37.5)	25 (62.5)	0.460*	-	-	31	15 (48.4)	16 (51.6)	0.702	-	-
**rs3212752**	TT	44	14 (31.8)	30 (68.2)				35	16 (45.7)	19 (54.3)			
	CC	0	0	0	0.395*	-	-	0	0	0	1*	-	-
	CT	6	3 (50)	3 (50)				4	2 (50)	2 (50)			
	C	6	3 (50)	3 (50)	0.395*	-	-	4	2 (50)	2 (50)	1*	-	-
	T	-	-	-	-	-	-	-	–	-	-	-	-

EULAR: European League Against Rheumatism; OR: odds ratio; CI: confidence interval; *: p value for Fisher's Exact Test

Table S32. Tofacitinib LDA bivariate demographic and clinical analyses

Clinical variable	3 months						6 months					
	N	LDA		OR	CI95%	p-value	N	LDA		OR	CI95%	p-value
		LDA	No LDA					LDA	No LDA			
**Sex**												
**Woman**	39	10 (25.6)	29 (74.4)	-	-	1*	34	5 (14.7)	29 (85.3)	-	-	0.213*
**Man**	11	2 (18.2)	9 (81.8)				**5**	2 (40)	3 (60)			
**Smoking**												
**Smoker**	6	1 (16.7)	5 (83.3)				5	1 (20)	4 (80)			
**Exsmoker**	14	3 (21.4)	11 (78.6)	-	-	1*	13	3 (23.1)	10 (76.9)	-	-	0.848*
**No smoker**	30	8 (26.7)	22 (78.6)				21	3 (14.3)	18 (85.7)			
**Age at Dx**	50	40.6 ±11.8	39.9 ±13.3	-	-	0.865	39	41.7 ±12.7	41.1 ±12.7	-	-	0.914
**Years with RA**	50	13 [8.7-16]	15[8.2-21.5]	-	-	0.833	39	10 [9.5-15.5]	13.5 [8-20]	-	-	0.766
**Years from Dx till JAK inhibitor treatment**	50	8 [2.7-12.2]	11.5 [5-16.7]	-	-	0.535	39	4 [3-9]	9.5 [4-15]	-	-	0.947
**JAK inhibitors start age**	50	53 [42.5-61]	56 [49-59]	-	-	0.708	39	50 [40.5-63]	55.5 [48-61]	-	-	0.951
**Treatment duration with JAK inhibitor (months)**	50	14.1 [11.7-28.7]	16.2 [5.7-42.1]	-	-	0.184	39	40.1 [17.1-59.2]	19.7 [12.4-43.1]	-	-	0.222
**Biologic-naïve**												
**Yes**	4	2 (50)	2 (50)	-	-	0.239*	4	0 (0)	4 (100)	-	-	1*
**No**	46	10 (21.7)	36 (78.3)				35	7 (20)	28 (80)			
**Number of previous BTs**	50	2 [1-2]	2 [1-3]	-	-	0.21	39	2 [1.5-2]	2 [1-3]	-	-	0.870
**Previous BTs duration (months)**	50	83.3 [34-137.4]	69.8 [25.3-131.5]	-	-	0.539	39	33.3 [25.34-99.6]	65.4 [45.7-132.9]	-	-	0.387
**BTs cause of suspension**												
**Primary failure**	12	1 (8.3)	11 (91.7)	-	-	0.252	8	2 (25)	6 (75)	-	-	0.647
**Secondary failure**	27	5 (18.5)	22 (81.5)	-	-	0.718	20	2 (10)	18 (90)	-	-	0.112
**Adverse events**	7	4 (57.1)	3 (42.9)	0.13	[0.02- 0.76]	0.031	7	3 (42.9)	4 (57.1)	-	-	0.124
**Toxicity**	-	-	-	-	-	-	-	-	-	-	-	-
**Others**	-	-	-	-	-	-	-	-	-	-	-	-
**Baseline RF (Qualitative)**												
**Pos**	37	8 (21.6)	29 (78.4)	-	-	0.706*	31	4 (50)	4 (50)	9.3	[1.5-665.8]	0.022*
**Neg**	13	4 (30.8)	9 (69.2)				8	3 (9.7)	28 (90.3)			
**Baseline ACPA**												
**Pos**	40	9 (30)	31 (70)	-	-	0.685*	31	5 (16.1)	26 (83.9)	-	-	0.617*
**Neg**	10	3 (30)	7 (70)				8	2 (25)	6 (75)			
**Baseline CCI**												
**Absence**	22	6 (27.3)	16 (72.7)				19	4 (21.1)	15 (78.9)			
**Low**	14	3 (21.4)	11 (78.6)	-	-	1*	12	1 (12.5)	7 (87.5)	-	-	1*
**High**	14	3 (21.4)	11 (78.6)				8	1 (12.5)	7 (87.5)			
**BMI**	50	32.1 ± 6.4	28.2 ±5.5	-	-	0.075	39	29.5 ± 5.8	29.1 ± 5.9	-	-	0.842
**JAK inhibitor dose change**												
**yes**	6	2 (22.7)	4 (77.3)	-	-	0.621*	6	2 (33.3)	4 (66.7)	-	-	0.290*

No	44	10 (22.7)	34 (77.3)				33	5 (15.2)	28 (84.8)			
**JAK inhibitor suspension**												
yes	35	9 (25.7)	26 (74.3)	-	-	1*	25	4 (16)	21 (78.6)	-	-	0.685*
No	15	3 (20)	12 (80)				14	3 (21.4)	11 (84)			
**JAK inhibitor cause of suspension**												
Primary failure	12	0	12 (100)	1.17×10^{8}	[1.52×10^{-173}- NA]	0.019*	4	0 (0)	4 (100)	4.27×10^{8}	[NA -NA]	0.020*
Secondary failure	12	5 (41.7)	7 (58.3)	0.52	[0.08– 2.96]		12	0 (0)	12 (100)	4.27×10^{8}	[2.13×10^{-289}- NA]	
Adverse events	11	3 (27.3)	8 (72.7)	1	1		9	3 (33.3)	6 (66.7)	1	1	
Others	1	1 (100)	0 (0)	1.19×10^{-9}	[NA - NA]		1	1 (100)	0 (0)	5.85×10^{-10}	[NA - NA]	
**BT after JAK inhibitor treatment**												
Yes	28	5 (17.9)	23 (82.1)	-	-	0.324*	19	1 (5.3)	18 (94.7)	-	-	0.091*
No	22	7 (31.8)	15 (68.2)				20	6 (30)	14 (70)			
**Adverse events to JAK inhibitor**												
Yes	20	5 (25)	15 (75)	-	-	1*	16	2 (12.5)	14 (87.5)	-	-	0.677*
No	30	7 (23.3)	23 (76.7)				23	5 (21.7)	18 (78.3)			
**Concomitant DMARDs**												
MTX	11	4 (36.4)	7 (63.6)	-	-	0.420*	8	2 (25)	6 (75)	-	-	0.804*
HXQ	2	1 (50)	1 (50)				2	0 (0)	2 (100)			
SSZ	0	0	0				0	0	0			
LFN	2	0	2 (100)				1	0 (0)	1 (100)			
None	35	7 (20)	28 (80)				28	5 (17.9)	23 (82.1)			
**Concomitant statins**												
Yes	16	3 (18.8)	13 (81.2)	-	-	0.727*	12	2 (16.7)	10 (83.3)	-	-	1*
No	34	9 (26.5)	25 (73.5)				27	5 (18.5)	22 (81.5)			
**Concomitant GC**												
Yes	35	4 (26.7)	11 (73.3)	-	-	1*	25	4 (16)	21 (84)	-	-	0.685*
No	15	8 (22.9)	27 (77.1)				14	3 (21.4)	11 (78.6)			
**Concomitant vitamin D**												
Yes	27	7 (25.9)	20 (74.1)	-	-	1*	21	5 (23.8)	16 (76.2)	-	-	0.417*
No	23	5 (21.7)	18 (78.3)				18	2 (11.1)	16 (88.9)			
**Baseline DAS28**	50	2.8 ± 0.4	4.1 ± 1.2	4.34	[1.75-16.05]	<0.001	39	2.7 [2.6-2.9]	3.65 [2.5-3.6]	2.82	[1.08-10.78]	0.002
**Baseline TJC**	50	1 [0-2.2]	4 [2-7.7]	1.42	[1.09-2.10]	<0.001	39	1 [1-1.5]	2.5 [0-4]	-	-	0.099
**Baseline SJC**	50	0 [0-0.2]	1 [0-3]	-	-	0.524	39	0 [0-0.5]	1 [0-2]	2.66	[1.05- 16.70]	0.007
**Baseline PVAS**	50	3.58 ± 1.6	5.1 ± 2.5	1.36	[1.01-1.92	0.010	39	5 [2- 5.5]	4.5 [2-5.25]	-	-	0.803
**Baseline MVAS**	50	2 [1-3.2]	4 [2-6]	1.47	[1.06- 2.18]	0.006	39	4 [1-5]	3 [2-5]	-	-	0.769
**Baseline CRP**	50	0.6 [0.1-2.4]	2.8 [0.9-7.8]	1.10	[0.99-1.38]	0.055	39	0.5 [0.4-2.9]	1.2 [0.5-5.4]	-	-	0.086
**Baseline ESR**	50	13 [9.75-31.5]	24 [15-44]	-	-	0.203	39	10 [7.5-12]	28 [14.5-37]	1.16	[1.04-1.39]	<0.001
**Baseline RF (Quantitative)**	50	55.5 [15.5-135.5]	42.5 [10.3-167.2]	-	-	0.665	39	50.5 [2-92]	63.5 [22.2-109.5]	1.01	[1.00-1.05]	0.011
**Baseline TC**	50	207.2± 25.3	210.3 ± 47.6	-	-	0.771	39	201 [168-213.5]	196 [183.7-222.7]	-	-	0.249
**Baseline LDL**	50	113 [93.7-137.5]	132 [100.2-150.7]	-	-	0.080	39	100 [97-147.5]	111[96- 131.2]	-	-	0.978
**Baseline TG**	50	109.5 [75.5- 133.5]	120.5 [84.2-179.7]	-	-	0.210	39	115 [101-140.5]	100 [64.75- 149.2]	-	-	0.639

LDA: low disease activity; TJC: tender joints count; SJC: swallowed joint count; PVAS: patient visual analogue scale; MVAS: physician visual analogue scale; RF: rheumatoid factor; ACPA: anti-citrullinated protein antibodies; ESR: erythrocyte sedimentation rate; CRP: C-reactive protein; TC: total cholesterol; LDL: low-density lipoprotein; TG: triglycerides; BMI: body mass index; CCI: Charlson Comorbidity Index; JAK inhibitor: Janus kinase inhibitor; BTs: biologic therapies; GC: glucocorticoids; DMARDs: disease-modifying antirheumatic drugs; MTX: methotrexate; HXQ:, hydroxychloroquine; LFN, leflunomide; SSZ: sulfasalazine; OR, odds ratio; CI, confidence interval; NA: not available (indicates non-estimable values due to sparse data or quasi-complete separation); *: p value for Fisher's Exact Test

Table S33. Tofacitinib LDA bivariate genetic analysis

SNPs	Genotipe	3 months					6 months				
		N	LDA		OR CI95%	p-valor	N	LDA		OR CI95%	p-valor
			LDA	No LDA				LDA	No LDA		
JAK1											
**rs2230587**	**GG**	41	10 (24.4)	31 (75.6)	-	1*	31	7 (22.6)	24 (77.4)	-	0.307*
	**AA**	0	0	0			0	0	0		
	**AG**	9	2 (22.2)	7 (77.8)			8	0 (0)	8 (100)		
	**A**	9	2 (22.2)	7 (77.8)	-	1*	8	0 (0)	8 (100)	-	0.307*
	**G**	-	-	-	-	-	-	-	-	-	-
**rs310241**	**GG**	2	0	2 (100)	-	0.852*	2	0 (0)	2 (100)		0.134*
	**AA**	30	7 (23.3)	23 (76.7)			23	2 (8.7)	21 (91.3)	-	
	**AG**	18	5 (27.8)	13 (72.2)			14	5 (35.7)	9 (64.3)	-	
	**A**	48	25 (11.5)	75 (36.4)	-	1*	37	7 (18.9)	30 (81.1)	-	1*
	**G**	20	5 (25)	15 (75)		1*	16	5 (31.2)	11 (68.8)	-	0.100*
**rs2230588**	**CC**	3	1 (33.3)	2 (66.7)	-	0.466*	3	1 (33.3)	2 (66.7)	1	0.010*
	**TT**	31	6 (19)	25 (80.6)			24	1 (4.2)	23 (95.8)	11.5 [0.36-389.40]	
	**CT**	16	5 (31.2)	11 (68.8)			12	5 (41.7)	7 (58.3)	0.7 [0.02-9.48]	
	**T**	47	11 (23.4)	36 (76.6)	-	1*	36	6 (16.7)	30 (83.3)	-	0.457*
	**C**	19	6 (31.6)	13 (68.4)	-	0.496*	15	6 (40)	9 (60)	15.33 [2.20- 311.97]	0.008*
**rs10889504**	**GG**	40	10 (25)	30 (75)	-	1*	32	7 (21.9)	25 (78.1)	-	0.312*
	**CC**	0	0	0			0	0	0		
	**CG**	10	2 (20)	8 (80)			7	0 (0)	7 (100)		
	**C**	10	2 (20)	8 (80)	-	1*	7	0 (0)	7 (100)	-	0.312*
	**G**	-	-	-	-	-	-	-	-	-	-
**rs2780815**	**GG**	8	3 (37.5)	5 (62.5)	-	0.332*	7	1 (14.3)	6 (85.7)	-	0.267*
	**TT**	12	1 (8.3)	11 (91.7)			9	0 (0)	9 (100)		
	**GT**	30	8 (26.7)	22 (73.3)			23	6 (26.1)	17 (73.9)		
	**T**	42	9 (21.4)	33 (78.6)	-	0.378*	32	6 (18.8)	26 (81.2)	-	1*
	**G**	38	11 (28.9)	27 (71.1)	-	0.248*	30	7 (23.3)	23 (76.7)		0.169*
JAK2											
**rs10119004**	**GG**	16	5 (31.2)	11 (68.8)	-	0.584*	13	1 (7.7)	12 (92.3)	-	0.326*
	**AA**	13	2 (15.4)	11 (84.6)			9	1 (11.1)	8 (88.9)		
	**AG**	21	5 (23.8)	16 (76.2)			17	5 (29.4)	12 (70.6)		
	**A**	34	7 (20.6)	27 (79.4)	-	0.485*	26	6 (23.1)	20 (76.9)	-	0.388*
	**G**	37	10 (27)	27 (73)		0.479*	30	6 (20)	24 (80)	-	1*
**rs7857730**	**GG**	15	3 (20)	12 (80)	-	0.767*	12	2 (16.7)	10 (83.3)	-	1*
	**TT**	15	3 (20)	12 (80)			11	2 (18.2)	9 (81.2)		
	**GT**	20	6 (30)	14 (70)			16	3 (18.8)	13 (81.2)		
	**G**	35	9 (25.7)	26 (74.3)		1*	28	5 (17.9)	23 (82.1)	-	1*
	**T**	35	9 (25.7)	26 (74.3)	-	1*	27	5 (18.5)	22 (81.5)	-	1*
**rs2274472**	**CC**	10	3 (30)	7 (70)	-	0.743*	7	2 (28.6)	5 (71.4)	-	0.5938*
	**TT**	13	2 (15.4)	11 (84.6)			8	2 (25)	6 (75)		
	**CT**	27	7 (25.9)	20 (74.1)			24	3 (12.5)	21 (87.5)		
	**C**	37	10 (27)	27 (73)	-	0.479*	31	5 (16.1)	26 (83.9)	-	0.617*
	**T**	40	9 (22.5)	31 (77.5)	-	0.685*	32	5 (15.6)	27 (84.4)	-	0.587*
**rs2230722**	**CC**	32	8 (25)	24 (75)	-	1*	26	5 (19.2)	21 (80.8)	-	1*
	**TT**	3	0 (0)	3 (100)			1	0 (0)	1 (100)		
	**CT**	15	4 (26.7)	11 (73.3)			12	2 (16.7)	10 (83.3)		
	**C**	47	12 (25.5)	35 (74.5)	-	1*	38	7 (18.4)	31 (81.6)	-	1*
	**T**	18	4 (22.2)	14 (77.8)	-	1*	13	2 (15.4)	11 (84.6)	-	1*
**rs2230724**	**GG**	18	5 (27.8)	13 (72.2)	-	0.916*	15	2 (13.3)	13 (86.7)	-	0.878*
	**AA**	13	3 (23.1)	10 (76.9)			9	2 (22.2)	7 (77.8)		
	**AG**	19	4 (21.1)	15 (78.9)			15	3 (20)	12 (80)		
	**A**	32	7 (21.9)	25 (78.1)	-	0.734*	24	5 (20.8)	19 (79.2)	-	0.685*
	**G**	37	9 (24.3)	28 (75.7)	-	1*	30	5 (16.7)	25 (83.3)	-	0.652*
JAK3											
**rs3212780**	**GG**	23	4 (17.4)	19 (82.6)	-	0.493*	17	1 (5.9)	16 (94.1)	-	0.112*
	**AA**	1	0 (0)	1 (100)			0	0	0		

	**AG**	26	8 (30.8)	18 (69.2)			22	6 (27.3)	16 (72.7)		
	**A**	27	8 (29.6)	19 (70.4)	-	0.344*	22	6 (27.3)	16 (72.7)		0.112*
	**G**	49	12 (24.5)	37 (75.5)	-	1*	-	-	-	-	-
**rs3008**	**GG**	14	6 (42.9)	8 (57.1)	1.15×10^{-8} (NA–NA)	0.048*	12	1 (8.3)	11 (91.7)	-	0.149*
	**AA**	10	0 (0)	10 (100)	1		8	0 (0)	8 (100)		
	**AG**	26	6 (23.1)	20 (76.9)	2.88×10^{-8} (NA)		19	6 (31.6)	13 (68.4)		
	**A**	36	6 (16.7)	30 (83.3)	-	0.070*	27	6 (22.2)	21 (77.8)	-	0.402*
	**G**	40	12 (30)	28 (70)	-	0.092*	31	7 (22.6)	24 (77.4)	-	0.307*
**rs3212752**	**TT**	44	10 (22.7)	34 (77.3)	-	0.621*	35	7 (20)	28 (80)	-	1*
	**CC**	0	0	0			0	0	0		
	**CT**	6	2 (33.3)	4 (66.7)			4	0 (0)	4 (100)		
	**C**	6	2 (33.3)	4 (66.7)	-	0.621*	4	0 (0)	4 (100)		1*
	**T**	-	-	-	-	-	-	-	-	-	-

LDA: low disease activity; OR: odds ratio; CI: confidence interval; *: NA: not available (indicates non-estimable values due to sparse data or quasi-complete separation); *: p value for Fisher's Exact Test.

Table S34. Tofacitinib remission bivariate demographic and clinical analyses

Clinical variable	3 months						6 months					
	N	Remisson		OR	$CI_{95\%}$	p-value	N	Remission		OR	$CI_{95\%}$	p-value
		Remission	No remission					Remission	No Remission			
**Sex**												
**Woman**	39	5 (12.8)	34 (8.2)	-	-	0.573*	33	10 (30.3)	23 (69.7)	-	-	1*
**Man**	11	0 (0)	11 (100)				5	1 (20)	4 (80)			
**Smoking**												
**Smoker**	6	0 (0)	6 (100)	-	-	1*	5	0 (0)	5 (100)	-	-	0.325*
**Exsmoker**	14	1 (7.1)	13 (92.9)				12	3 (25)	9 (75)			
**No smoker**	30	4 (13.3)	26 (86.7)				21	8 (38.1)	13 (61.9)			
**Age at Dx**	50	36.4 ±17.9	40.5 ± 12.4	-	-	0.638	38	37.4 ± 14.4	43.1 ± 11.7	-	-	0.267
**Years with RA**	50	11 [8-22]	15 [9-20]	-	-	0.864	38	10 [7.5-12.5]	15 [9.5-20]	-	-	0.105
**Years from Dx till JAK inhibitor treatment**	50	6 [5-15]	10 [4-16]	-	-	0.571	38	7 [4.5-9.5]	12 [4-15.5]	-	-	0.155
**JAK inhibitors start age**	50	46 [43-56]	56 [49-61]	-	-	0.418	38	49 [40.5-54]	57 [50-63]	1.06	[1-1.13]	0.064
**Treatment duration with JAK inhibitor (Months)**	50	29.3 [12.1-50]	13.7 [8.1-40.1]	-	-	0.298	38	20.8 [12-39.6]	20.8 [12.5-45.9]	-	-	0.596
**Bionaive**												
**Yes**	4	1 (25)	3 (75)	-	-	0.353*	4	1 (25)	3 (75)	-	-	1*
**No**	46	4 (8.7)	42 (91.3)				35	11 (31.4)	24 (68.6)			
**Number of previous BTs**	46	2 [1-3]	2 [1-3]	-	-	0.790	38	2 [1-2]	2 [1-3.5]	-	-	0.679
**Previous BTs duration (months)**	46	101.5 [48.1-157.1]	62.9 [30.1-131.2]	-	-	0.487	38	56.4 [47.9-109.7]	69.5 [32.5-137.7]	-	-	0.783
**BTs cause of suspension**												
**Primary failure**	12	1 (8.3)	11 (91.7)	-	-	1*	8	3 (37.5)	5 (62.5)	-	-	0.666*
**Secondary failure**	27	2 (7.4)	25 (92.6)	-	-	1*	19	6 (31.6)	13 (68.4)	-	-	1*
**Adverse events**	7	1 (14.3)	6 (85.7)	-	-	0.496*	7	1 (14.3)	6 (85.7)	-	-	0.644*
**Toxicity**	-	-	-	-	-	-	-	-	-	-	-	-
**Others**	-	-	-	-	-	-	-	-	-	-	-	-
**Baseline RF (Cualitative)**												
**Pos**	37	1 (2.7)	36 (97.3)	16	[2.06-334.40]	0.013*	31	7 (22.6)	24 (77.4)	-	-	0.160*
**Neg**	13	4 (30.8)	9 (69.2)				7	4 (57.1)	3 (42.9)			
**Baseline ACPA**												
**Pos**	40	4 (10)	36 (90)	-	-	1*	31	9 (29)	22 (71)	-	-	1*
**Neg**	10	1 (10)	9 (90)				7	2 (28.6)	5 (71.4)			
**Baseline CCI**												
**Absence**	22	4 (18.2)	18 (81.8)	-	-	0.255*	18	8 (44.4)	10 (55.6)	-	-	0.207*
**Low**	14	0 (0)	14 (100)				8	1 (12.5)	7 (87.5)			
**High**	14	1 (7.1)	13 (92.9)				12	2 (16.7)	10 (83.3)			
**BMI**	50	28.6 ± 3.8	29.2 ± 6.2	-	-	0.786*	38	26.5 ± 4.6	29.9 ± 5.9	1.12	[0.98-1.31]	0.070
**JAK inhibitor dose change**												
**yes**	6	1 (16.7)	5 (83.3)	-	-	0.487*	6	4 (66.7)	2 (33.3)	0.14	[0.01-0.8]	0.046*
**No**	44	4 (9.1)	49 (90.9)				32	7 (21.9)	25 (78.1)			
**JAK inhibitor suspension**												
**yes**	35	2 (5.7)	33 (94.3)	-	-	0.151*	24	4 (16.7)	20 (83.3)	5	[1.16-24.57]	0.060*
**No**	15	3 (20)	12 (80)				14	7 (50)	7 (50)			
**JAK inhibitor cause of suspension**												
**Primary failure**	12	0 (0)	12 (100)	-	-	0.771*	4	0 (0)	4 (100)	-	-	1*
**Secondary failure**	12	1 (8.3)	11 (91.7)				12	2 (16.7)	10 (83.3)			
**Adverse events**	11	1 (9.1)	10 (90.9)				9	2 (22.2)	7 (77.8)			
**Others**	1	0 (0)	1 (100)				0	0	0			
**BT after JAK inhibitor treatment**												
**Yes**	28	1 (3.6)	27 (96.4)	-	-	0.155*	18	4 (22.2)	14 (77.8)	-	-	0.484*
**No**	22	4 (18.2)	18 (81.8)				20	7 (35)	13 (65)			
**Adverse events to JAK inhibitor**												
**Yes**	20	2 (10)	18 (90)	-	-	1*	16	3 (18.8)	13 (81.2)	-	-	0.296*
**No**	30	3 (10)	27 (90)				22	8 (36.4)	14 (63.6)			
**Concomitant DMARDs**												
**MTX**	11	1 (9.1)	10 (90.9)	-	-	1*	8	1 (12.5)	7 (87.5)	-	-	0.262
**HXQ**	2	0 (0)	2 (100)				2	0 (0)	2 (100)			
**SSZ**	0	0	0				0	0	0			
**LFN**	2	0 (0)	2 (100)				1	1 (100)	0 (0)			
**None**	35	4 (11.4)	31 (88.6)				27	9 (33.3)	18 (88.7)			
**Concomitant statins**												
**Yes**	16	1 (6.2)	15 (93.8)	-	-	1*	12	2 (16.7)	10 (83.3)	-	-	0.443*
**No**	34	4 (11.8)	30 (88.2)				26	9 (34.6)	17 (65.4)			
**Concomitant GC**												
**Yes**	35	2 (5.7)	33 (94.3)	-	-	0.151*	24	7 (29.2)	17 (70.8)	-	-	1*
**No**	15	3 (20)	12 (80)				14	4 (28.6)	10 (71.4)			
**Concomitant vitamin D**												
**Yes**	27	2 (7.4)	25 (92.6)	-	-	0.650*	20	4 (20)	16 (80)	-	-	0.287*
**No**	23	3 (13)	20 (87)				18	7 (38.9)	11 (61.1)			
**Baseline DAS28**	50	2.2 ± 0.3	3.9 ± 1.1	41.27	[4.36-1788.46]	<0.001	38	2.2 [2.1-2.3]	3.8 [3.2-4.5]	8.03×10^{81}	[0-NA]	<0.001
**Baseline TJC**	50	2 [0-2]	3 [1-7]	1.42	[0.99-2.76]	0.002	38	0 [0-0.5]	3 [2-5.5]	5.35	[2.05-24.86]	<0.001
**Baseline SJC**	50	0 [0-0]	1 [0-3]	1.82	[0.94-9.34]	0.009	38	0 [0-0]	1 [0-2]	3.46	[1.31-16.12]	0.005
**Baseline PVAS**	50	4.2 ± 3.4	4.8 ± 2.3	-	-	0.690	38	2 [2-3]	5 [3-6]	1.42	[1-2.22]	0.090
**Baseline MVAS**	50	2 [0-2]	4 [2-6]	1.95	[1.11-4.83]	0.027	38	1.45 ± 1.29	4.07 ± 2.2	2.39	[1.41-5.14]	<0.001
**Baseline CRP**	50	1.2 [0.3-3]	2.5 [0.5-5.7]	1.11	[0.96-1.68]	0.055	38	1.4 [1.1-2.8]	0.7 [0.4-4.25]	-	-	0.270
**Baseline ESR**	50	18 [17-32]	23 [11-43]	-	-	0.906	38	15 [9-30]	27 [12-35.5]	-	-	0.716
**Baseline RF (Quantitative)**	50	5 [5-10]	55 [16.8-159]	1	[0.99-1.02]	0.080	38	61 [8-67.5]	65 [23-111]	-	-	0.304
**Baseline TC**	50	165 ± 22.5	214.5 ± 42.1	1.05	[1.01-1.10]	0. 003	38	186 [180-209.5]	201 [182.5-224]	-	-	0.168
**Baseline LDL**	50	98 [86-112]	129 [99-145]	1.03	[0.99-1.08]	0. 053	38	96 [75-105]	120 [99.5-137.5]	1.05	[1.01-1.10]	0.007
**Baseline TG**	50	85 [58-166]	113 [84-166]	-	-	0.767	38	65 [48.5-163.5]	112 [83-133]	-	-	0.775

TJC: tender joints count; SJC: swallowed joint count; PVAS: patient visual analogue scale; MVAS: physician visual analogue scale; RF: rheumatoid factor; ACPA: anti-citrullinated protein antibodies; ESR: erythrocyte sedimentation rate; CRP: C-reactive protein; TC: total cholesterol; LDL: low-density lipoprotein; TG: triglycerides; BMI: body mass index; CCI: Charlson Comorbidity Index; JAK inhibitor: Janus kinase inhibitor; BTs: biologic therapies; GC: glucocorticoids; DMARDs: disease-modifying antirheumatic drugs; MTX: methotrexate; HXQ:, hydroxychloroquine; LFN, leflunomide; SSZ: sulfasalazine; OR, odds ratio; CI, confidence interval; NA: not available (indicates non-estimable values due to sparse data or quasi-complete separation); *: p value for Fisher's Exact Test

Table S35. Tofacitinib remission bivariate genetic analysis

SNPs	Genotipe	3 meses					6 meses				
		N	Remission		OR CI$_{95\%}$	p-value	N	Remission		OR CI$_{95\%}$	p-value
			Remission	No Remission				Remission	No Remission		
JAK1											
**rs2230587**	GG	41	4 (9.8)	37 (90.2)			30	8 (26.7)	22 (73.3)		
	AA	0	0	0	-	1*	0	0	0	-	0.667*
	AG	9	1 (11.1)	8 (88.9)			8	3 (37.5)	5 (62.5)		
	A	9	1 (11.1)	8 (88.9)	-	1*	8	3 (37.5)	5 (62.5)	-	0.667*
	G	-	-	-	-	-	-	-	-	-	-
**rs310241**	GG	2	0 (0)	2 (100)			2	1 (50)	1 (50)		
	AA	30	3 (10)	27 (90)	-	1*	23	6 (26.1)	17 (73.9)	-	0.854*
	AG	18	2 (11.1)	16 (88.9)			13	4 (30.8)	9 (69.2)		
	A	48	5 (10.4)	43 (89.6)	-	1*	36	10 (27.8)	26 (72.2)	-	0.500*
	G	20	2 (10)	18 (90)	-		15	5 (33.3)	10 (66.7)	-	0.721*
**rs2230588**	CC	3	0 (0)	3 (100)			2	1 (50)	1 (50)		
	TT	31	4 (12.9)	27 (87.1)	-	0.745*	24	7 (29.2)	17 (70.8)	-	0.723*
	CT	16	1 (6.2)	15 (93.8)			12	3 (25)	9 (75)		
	T	47	5 (10.6)	42 (89.4)	-	1*	36	10 (27.8)	26 (72.2)	-	0.500*
	C	19	1 (5.3)	18 (94.7)	-	0.637	14	4 (28.6)	10 (71.4)	-	1*
**rs10889504**	GG	40	4 (10)	36 (90)			31	8 (25.8)	23 (74.2)		
	CC	0	0	0	-	1*	0	0	0	-	0.390*
	CG	10	1 (10)	9 (90)			7	3 (42.9)	4 (57.1)		
	C	10	1 (10)	9 (90)	-	1*	7	3 (42.9)	4 (57.1)		0.390*
	G	-	-	-	-	-	-	-	-	-	-
**rs2780815**	GG	8	0 (0)	8 (100)			7	1 (14.3)	6 (85.7)		
	TT	12	2 (16.7)	10 (83.3)	-	0.660*	9	3 (33.3)	6 (66.7)	-	0.783*
	GT	30	3 (10)	27 (90)			22	7 (31.8)	15 (68.2)	-	
	T	42	5 (11.9)	37 (88.1)	-	0.577*	31	10 (32.2)	21 (67.7)	-	0.648*
	G	38	3 (7.9)	35 (92.1)		0.581*	29	8 (27.6)	21 (72.4)	-	1*
JAK2											
rs10119004	GG	16	1 (6.2)	15 (93.8)			13	2 (15.4)	11 (84.6)	16.50 [2.19-201.83]	
	AA	13	3 (23.1)	10 (76.9)	-	0.246*	8	6 (75)	2 (25)	1	0.011*
	AG	21	1 (4.8)	20 (95.2)			17	3 (17.6)	14 (82.4)	14 [2.12-138.41]	
	A	34	4 (11.8)	30 (88.2)	-	1*	25	9 (36)	16 (64)	-	0.267*
	G	37	2 (5.4)	35 (94.6)	-	0.103*	30	5 (16.7)	25 (83.3)	15 [2.64-126.22]	0.003*
**rs7857730**	GG	15	0 (0)	15 (100)	1 [$4.16 \times 10^{-140} - 2.39 \times 10^{139}$]		12	2 (16.7)	10 (83.3)	1	
	TT	20	5 (33.3)	10 (66.7)	2.34×10^{-9} [NA-467.28]	0.002*	10	7 (70)	3 (30)	0.08 [0.01-0.56]	0.005*
	GT	20	0 (0)	20 (100)	1		16	2 (12.5)	14 (87.5)	1.40 [0.14-13.35]	
	G	35	0 (0)	35 (100)	2.34×10^{-9} [NA-7.46×10^{129}]	0.001*	28	4 (14.3)	24 (85.7)	0.07 [0.01-0.36]	0.002*
	T	35	5 (14.3)	30 (85.7)	-	0.305*	26	9 (34.6)	17 (65.4)	-	0.443*
**rs2274472**	CC	10	0 (0)	10 (100)			7	1 (14.3)	6 (85.7)	1	
	TT	13	3 (23.1)	10 (76.9)	-	0.234*	7	6 (85.7)	1 (14.3)	0.02 [0.0006-0.37]	0.002*
	CT	27	2 (7.4)	25 (92.6)			24	4 (16.7)	20 (83.3)	0.83 [0.03-7.139]	
	C	37	2 (5.4)	35 (94.6)	-	0.103*	31	5 (16.1)	26 (83.9)	0.03 [0.0015-0.23]	0.001*
	T	40	5 (12.5)	35 (87.5)	-	0.568*	31	10 (32.3)	21 (67.7)	-	0.648*
**rs2230722**	CC	32	3 (9.4)	29 (90.6)			26	6 (23.1)	20 (76.9)		
	TT	3	0 (0)	3 (100)	-	0.745*	1	1 (100)	0 (0)	-	0.212*
	CT	15	2 (13.3)	13 (86.7)			11	4 (36.4)	7 (68.6)		
	C	47	5 (10.6)	42 (89.4)	-	1*	37	10 (27)	27 (73)	-	0.289*
	T	18	2 (11.1)	16 (88.9)	-	1*	12	5 (41.7)	7 (58.3)	-	0.272*
**rs2230724**	GG	18	1 (5.6)	17 (94.4)	7.6 (0.94–160.2)		15	3 (20)	12 (80)	12 [1.79-119.60]	
	AA	13	4 (30.8)	9 (69.2)	-	0.016*	8	6 (75)	2 (25)	1	0.007*
	AG	19	0 (0)	19 (100)	1.40×10^{8} [2.69×10^{-107}-NA]		15	2 (13.3)	13 (86.7)	19.5 [2.63-236.48]	
	A	32	4 (12.5)	28 (87.5)	-	0.641*	23	8 (34.8)	15 (65.2)	-	0.47*
	G	37	1 (2.7)	36 (97.3)	16 [2.06-334.40]	0.013*	30	5 (16.7)	25 (83.3)	15 [2.64-126.22]	0.003*
JAK3											
**rs3212780**	GG	23	4 (17.4)	19 (82.6)			17	7 (41.2)	10 (58.8)		
	AA	1	0 (0)	1 (100)	-	0.255*	0	0	0	-	0.166*
	AG	26	1 (3.8)	25 (96.2)			21	4 (19)	17 (81)		
	A	27	1 (3.7)	26 (96.3)	-	0.166*	21	4 (19)	17 (81)	-	0.166*
	G	49	5 (10.2))	44 (89.8)	-	1*	-	-	-	-	-
**rs3008**	GG	14	1 (7.1)	13 (92.9)			12	3 (25)	9 (75)		
	AA	10	2 (20)	8 (80)	-	0.478*	8	2 (25)	6 (75)	-	0.901*
	AG	26	2 (7.7)	24 (92.3)			18	6 (33.3)	12 (66.7)		
	A	36	4 (11.1)	32 (88.9)	-	1*	26	8 (30.8)	18 (69.2)	-	1*
	G	40	3 (7.5)	37 (92.5)	-	0.258*	30	9 (30)	21 (70)	-	1*
**rs3212752**	TT	44	4 (9.1)	40 (90.9)			34	9 (26.5)	25 (73.5)		
	CC	0	0	0	-	0.487*	0	0	0	-	0.564*
	CT	6	1 (16.7)	5 (83.3)			4	2 (50)	2 (50)		
	C	6	1 (16.7)	5 (83.3)	-	0.487*	4	2 (50)	2 (50)		0.564*
	T	-	-	-	-	-	-	-	-	-	-

OR: odds ratio; CI: confidence interval; NA: not available (indicates non-estimable values due to sparse data or quasi-complete separation); *: p value for Fisher's Exact Test
