# Supplementary material for: Clinical and Pharmacogenetic Factors Associated with Response to JAK Inhibitors in Patients with Rheumatoid Arthritis: A Real-World Study of JAK1, JAK2, and JAK3 Gene Variants
Source: Pharmaceutics. 2026 Jul 11;18(7):846. doi: 10.3390/pharmaceutics18070846 (PMC13415438; doi:10.3390/pharmaceutics18070846)
Supplement: Supplementary file 1 [file pharmaceutics-18-00846-s001.zip › Table S36-S41. Predictors of EULAR response LDA and remission at 3 and 6 months in RA patients treated with baricitinib (Bivariate analyisis).pdf]

Table S36. Baricitinib EULAR response bivariate demographic and clinical analyses

Clinical variables	3 months						6 months					
	N	EULAR response		OR	CI95%	p-value	N	EULAR response		OR	CI95%	p-value
		Satisfactory	Unsatisfactory					Satisfactory	Unsatisfactoria			
**Sex**												
Woman	39	17 (43.6)	22 (56.4)	-	-	0.633*	32	15 (46.9)	17 (53.1)	-	-	0.124*
Man	5	1 (20)	4 (80)				4	0 (0)	4 (100)			
**Smoking**												
Smoker	10	4 (40)	6 (60)	-	-	1*	8	3 (37.5)	5 (62.5)	-	-	0.266*
Exsmoker	7	3 (42.9)	4 (57.1)				7	1 (14.3)	6 (85.7)			
No smoker	27	11 (40.7)	16 (59.3)				21	11 (52.4)	10 (47.6)			
**Age at Dx**	44	41.7±13.2	43.2±9.7	-	-	0.686	36	39.4±12.2	46.6±10.1	0.93	0.86-1	0.072
**Years with RA**	44	16.0 (11.2-20)	11.5 (8.2-21.5)	-	-	0.422	36	16.1 ± 9.4	13.4±6.3	-	-	0.358
**Years from Dx till JAK inhibitor treatment**	44	10.1 (7.4-15)	7.9 (5.2-14.8)	-	-	0.440	36	9.7 (7.4-15)	7.2 (5.1-12.5)	-	-	0.195
**JAKi start age**	44	55 (50.7-60)	55 (48-60.7)	-	-	0.922	36	51.8± 10.2	55.3± 9.4	-	-	0.293
**Treatment duration with JAK inhibitor (months)**	44	51.5±29.9	27.3±25.1	1.03	1.01-1.06	0.008	36	51 (23.6-71.4)	41.7 (12.3-55.6)	-	-	0.313
**Biologic-naïve**												
Yes	4	4 (100)	0 (0)	NA	NA-NA	0.022*	4	3 (75)	1 (25)	-	-	0.287*
No	40	14 (35)	26 (65)	1	-		32	12 (37.5)	20 (62.5)			
**Number of previous BTs**	44	2 (1-2.8)	2.5 (1.3-4)	-	-	0.202	36	2 (1-3.5)	2 (1-4)	-	-	0.775
**Previous BTs duration (months)**	44	65.8 (13.6-153.7)	56.8 (20.7-123.0)	-	-	0.425	36	85.3 (16.6-191)	38.2 (14.5-101.26)	-	-	0.157
**BTs cause of suspension**												
Primary failure	10	3 (30)	7 (70)	-	-	0.799*	8	3 (37.5)	5 (62.5)	-	-	0.460*
Secondary failure	22	8 (36.4)	14 (63.6)				17	7 (41.2)	10 (58.8)			
Adverse events	6	2 (33.3)	4 (66.7)				6	1 (16.7)	5 (83.3)			
Toxicity	1	0 (0)	1 (100)				0	-	-			
Others	1	1 (100)	0 (0)				1	1 (100)	0 (0)			
**Baseline RF (Cualitative)**												
Pos	38	16 (42.1)	22 (57.9)	-	-	1*	30	13 (43.3)	17 (56.7)	-	-	1*
Neg	6	2 (33.3)	4 (66.7)				6	2 (33.3)	4 (66.7)			
**Baseline ACPA**												
Pos	37	15 (40.5)	22 (59.5)	-	-	1*	30	12 (40.0)	18 (60.0)	-	-	0.677*
Neg	7	3 (42.9)	4 (57.1)				6	3 (50.0)	3 (50.0)			
**Baseline CCI**												
Absence	18	8 (44.4)	10 (55.6)	-	-	0.669*	15	8 (53.3)	7 (46.7)	-	-	0.463
Low	18	8 (44.4)	10 (55.6)				14	5 (35.7)	9 (64.3)			
High	8	2 (25)	6 (75)				7	2 (28.6)	5 (71.4)			
**BMI**	44	25.6 (24.3-28)	26.8 (24.9-29.9)	-	-	0.685	36	26.1 (24.1-26.5)	27.3 (24.9-30)	0.79	0.60-0.98	0.038
**JAK inhibitor dose change**												
Yes	9	7 (77.8)	2 (22.2)	7.63	1.55-57.22	0.011	9	5 (55.6)	4 (44.4)	-	-	0.442*
No	35	11 (31.4)	24 (68.6)	1	-		27	10 (37)	17 (63)			
**JAK inhibitor suspensión**												
yes	21	5 (23.8)	16 (76.2)	1	-	0.027	14	2 (14.3)	12 (85.7)	1	-	0.007
No	23	13 (56.5)	10 (43.5)	4.16	1.19-16.46		22	13 (59.1)	9 (40.9)	2.12	0.45-10.46	
**JAK inhibitor cause of suspension**												
Primary failure	5	1 (20.0)	4 (80)	-	-	1*	2	0 (0)	2 (100)	-	-	1*
Secondary failure	10	3 (30)	7 (70)				8	1 (12.5)	7 (87.5)			
Adverse events	6	1 (16.7)	5 (83.3)				4	1 (25)	3 (75)			
Others	0	-	-				0	-	-			
**BT after JAK inhibitor treatment**												
yes	17	3 (17.6)	14 (82.4)	1	-	0.012	10	1 (10)	9 (90)	1	-	0.016
No	27	15 (55.6)	12 (44.4)	5.83	1.49-29.78		26	14 (53.8)	12 (46.2)	10.49	1.63-208.35	
**Adverse events to JAK inhibitor**												
yes	16	7 (43.8)	9 (56.2)	-	-	0.772	13	7 (53.8)	6 (46.2)	-	-	0.265*
No	28	11 (39.3)	17 (60.7)				23	8 (34.8)	15 (65.2)			
**Concomitant DMARDs**												
MTX	10	5 (50)	5 (50)	-	-	1*	7	5 (71.4)	2 (28.6)	-	-	0.330*
HXQ	1	1 (100)	0 (0)				1	0 (0)	1 (100)			
SSZ	0	-	-				0	-	-			
LFN	3	1 (33.3)	2 (66.7)				3	1 (33.3)	2 (66.7)			
None	30	11 (36.7)	19 (63.3)				25	9 (36)	16 (64)			
**Concomitant statins**												
yes	19	9 (47.4)	10 (52.6)	-	-	0.447	19	7 (36.8)	12 (63.2)	-	-	0.534
No	25	9 (36)	16 (64)				17	8 (47.1)	9 (52.9)			
**Concomitant GC**												
yes	31	8 (25.8)	23 (74.2)	1	-	0.001	23	6 (26.1)	17 (73.9)	1	-	0.011
No	13	10 (76.9)	3 (23.1)	9.58	2.29-51.64		13	9 (69.2)	4 (30.8)	6.38	1.51-31.77	
**Concomitant vitamin D**												
yes	21	8 (38.1)	13 (61.9)	-	-	0.716	18	6 (33.3)	12 (66.7)	-	-	0.310
No	23	10 (43.5)	13 (56.5)				18	9 (50)	9 (50)			
**Baseline DAS28**	44	3.96±1.58	4.61±1.09	-	-	0.139	36	4.37±1.51	4.39±1.22	-	-	0.954
**Baseline TJC**	44	4.0 (1.3-7.8)	6.0 (4.0-8.7)	-	-	0.353	36	5 (3.5-6.5)	7 (3-9)	-	-	0.756
**Baseline SJC**	44	1.5 (0.3-3.8)	2.5 (0-4)	-	-	0.761	36	3 (0.5-4.5)	2 (0-4)	-	-	0.418
**Baseline PVAS**	44	5 (2.3-7.8)	7 (5-8)	-	-	0.111	36	7 (3-8)	7 (5-8)	-	-	0.441
**Baseline MVAS**	44	5 (2.3-6.8)	6 (4-7)	-	-	0.259	36	5 (3-7)	6 (5-7)	-	-	0.620
**Baseline CRP**	44	4.4 (1.7-8.5)	4.4 (1.5-8.9)	-	-	0.376	36	4.2 (2.1-8.2)	4.6 (1.3-8.7)	-	-	0.548
**Baseline ESR**	44	11 (9-40.5)	30 (10.3-46)	-	-	0.547	36	10 (8.5-48.5)	30 (13-40)	-	-	0.659
**Baseline RF (Quantitative)**	44	80.5 (34.7-108.7)	60.5 (23.2-197.2)	-	-	0.158	36	44 (26-107.5)	61 (18-160)	-	-	0.213
**Baseline TC**	44	204.7±41.6	190.6±53.4	-	-	0.332	36	189 (170.5-220.5)	206 (182-240)	-	-	0.297
**Baseline LDL**	44	127.3±36.5	115.5±32.6	-	-	0.275	36	127.8±41.6	117.5±28.2	-	-	0.417
**Baseline TG**	44	89 (69.5-117.5)	90.5 (75.7-119.2)	-	-	0.519	36	87 (62-118.5)	106 (75-141)	-	-	0.786

EULAR: European League Against Rheumatism; TJC: tender joints count; SJC: swallowed joint count; PVAS: patient visual analogue scale; MVAS: physician visual analogue scale; RF: rheumatoid factor; ACPA: anti-citrullinated protein antibodies; ESR: erythrocyte sedimentation rate; CRP: C-reactive protein; TC: total cholesterol; LDL: low-density lipoprotein; TG: triglycerides; BMI: body mass index; CCI: Charlson Comorbidity Index; JAK inhibitor: Janus kinase inhibitor; BTs: biologic therapies; GC: glucocorticoids; DMARDs: disease-modifying antirheumatic drugs; MTX: methotrexate; HXQ:, hydroxychloroquine; LFN, leflunomide; SSZ: sulfasalazine; OR, odds ratio; CI, confidence interval; NA: not available (indicates non-estimable values due to sparse data or quasi-complete separation); *: p value for Fisher's Exact Test

Table S37. Baricitinib EULAR response bivariate genetic analyses

SNPs	Genotipe	3 months					6 months				
		N	EULAR response		OR CI95%	p-value	N	EULAR response		OR CI95%	p-value
			Satisfactory	Unsatisfactory				Satisfactory	Unsatisfactory		
JAK1											
**rs2230587**	**GG**	30	10 (33.3)	20 (66.7)	-	0.231*	25	11 (44)	14 (56)	-	1*
	**AA**	1	1 (100)	0 (0)			1	0 (0)	1 (100)		
	**AG**	13	7 (53.8)	6 (46.2)			10	4 (40)	6 (60)		
	**A**	14	8 (57.1)	6 (42.9)	-	0.134	11	4 (36.4)	7 (63.6)	-	0.668
	**G**	43	17 (39.5)	26 (60.5)	-	0.409*	35	15 (42.9)	20 (57.1)	-	1*
**rs310241**	**GG**	5	1 (20)	4 (80)	-	0.265*	5	0 (0)	5 (100)	1	0.079
	**AA**	25	13 (52)	12 (48)			18	10 (55.6)	8 (44.4)	2.65×10^{7} (NA-NA)	
	**AG**	14	4 (28.6)	10 (71.4)			13	5 (38.5)	8 (61.5)	5.31×10^{7} (NA-NA)	
	**A**	39	17 (43.6)	22 (56.4)	-	0.633*	31	15 (48.4)	16 (51.6)	3.99×10^{7} (NA-NA)	0.041
	**G**	19	5 (26.3)	14 (73.7)	0.33 (0.08-1.15)	0.086	18	5 (27.8)	13 (72.2)	0.31 (0.07-1.19)	0.090
**rs2230588**	**CC**	4	1 (25)	3 (75)	-	0.282*	4	0 (0)	4 (100)	-	0.113*

	**TT**	25	13 (52)	12 (48)			18	10 (55.6)	8 (44.4)		
	**CT**	15	4 (26.7)	11 (73.3)			14	5 (35.7)	9 (64.3)		
	**T**	40	17 (42.5)	23 (57.5)	-	0.633*	32	15 (46.9)	17 (53.1)	3.75 × 10^7 (NA-NA)	0.073
	**C**	19	5 (26.3)	14 (73.7)	0.33 (0.08-1.15)	0.086	18	5 (27.8)	13 (72.2)	0.30 (0.07-1.19)	0.090
**rs10889504**	**GG**	32	9 (28.1)	23 (71.9)	1	0.007*	27	10 (37)	17 (63)	-	0.235*
	**CC**	1	1 (100)	0 (0)	3.99×10^7 (NA-NA)		1	0 (0)	1 (100)		
	**GC**	11	8 (72.7)	3 (27.3)	5.86×10^6 (NA-NA)		8	5 (62.5)	3 (37.5)		
	**C**	11	8 (72.7)	3 (27.3)	6.13 (1.45-32.79)	0.013	8	5 (62.5)	3 (37.5)	-	0.235*
	**G**	43	17 (39.5)	26 (60.5)	-	0.409*	20	15 (42.9)	20 (57.1)	-	1*
**rs2780815**	**GG**	15	6 (40.0)	9 (60.0)	-	0.527*	15	6 (40.0)	9 (60.0)	-	0.169
	**TT**	11	6 (54.5)	5 (45.5)			7	5 (71.4)	2 (28.6)		
	**GT**	18	6 (33.3)	12 (66.7)			14	4 (28.6)	10 (71.4)		
	**T**	29	12 (41.4)	17 (58.6)	-	0.929	21	9 (42.9)	12 (57.1)	-	1*
	**G**	33	12 (36.4)	21 (63.6)	-	0.288	29	10 (34.5)	19 (65.5)	0.21 (0.03-1.17)	0.075
JAK2											
**rs10119004**	**GG**	12	6 (50.0)	6 (50.0)	-	0.380	11	7 (63.6)	4 (36.4)	-	0.270
	**AA**	15	4 (26.7)	11 (73.3)			11	3 (27.3)	8 (72.7)		
	**AG**	17	8 (47.1)	9 (52.9)			14	5 (35.7)	9 (64.3)		
	**A**	32	12 (37.5)	20 (62.5)	-	0.452	25	8 (32.0)	17 (68.0)	-	0.076
	**G**	29	14 (48.3)	15 (51.7)	-	0.167	25	12 (48.0)	13 (52.0)	-	0.245
**rs7857730**	**GG**	8	2 (25.0)	6 (75.0)	-	0.313*	7	4 (57.1)	3 (42.9)	-	0.569
	**TT**	18	6 (33.3)	12 (66.7)			14	6 (42.9)	8 (57.1)		
	**GT**	18	10 (55.6)	8 (44.4)			15	5 (33.3)	10 (66.7)		
	**G**	26	12 (46.2)	14 (53.8)	-	0.395	22	9 (40.9)	13 (59.1)	-	0.908
	**T**	36	16 (44.4)	20 (55.6)	-	0.439*	29	11 (37.9)	18 (62.1)	-	0.417*
**rs2274472**	**CC**	3	0 (0)	3 (100)	-	0.237*	2	2 (100)	0 (0)	-	0.115*
	**TT**	17	9 (52.9)	8 (47.1)			14	7 (50)	7 (50)		
	**CT**	24	9 (37.5)	15 (62.5)			20	6 (30.0)	14 (70.0)		
	**C**	27	9 (33.3)	18 (66.7)	-	0.197	22	8 (36.4)	14 (63.6)	-	0.418
	**T**	41	18 (43.9)	23 (56.1)	-	0.257*	34	13 (38.2)	21 (61.8)	-	0.166*
**rs2230722**	**CC**	27	11 (40.7)	16 (59.3)	-	0.581*	24	11 (45.8)	13 (54.2)	-	0.837*
	**TT**	2	0 (0)	2 (100)			1	0 (0)	1 (100)		
	**CT**	15	7 (46.7)	8 (53.3)			11	4 (36.4)	7 (63.6)		
	**C**	42	18 (42.9)	24 (57.1)	-	0.505*	35	15 (42.9)	20 (57.1)	-	1*
	**T**	17	7 (41.2)	10 (58.8)	-	0.977*	12	4 (33.3)	8 (66.7)	-	0.473
**rs2230724**	**GG**	10	3 (30.0)	7 (70.0)	-	0.420	9	5 (55.6)	4 (44.4)	-	0.404
	**AA**	17	6 (35.3)	11 (64.7)			13	6 (46.2)	7 (53.8)		
	**AG**	17	9 (52.9)	8 (47.1)			14	4 (28.6)	10 (71.4)		
	**A**	34	15 (44.1)	19 (55.9)	-	0.424	27	10 (37)	17 (63)	-	0.442*
	**G**	27	12 (44.4)	15 (55.6)	-	0.547	23	9 (39.1)	14 (60.9)	-	0.681
JAK3											
**rs3212780**	**GG**	17	8 (47.1)	9 (52.9)	-	0.828*	12	7 (58.3)	5 (41.7)	-	0.436*
	**AA**	5	2 (40)	3 (60)			4	1 (25)	3 (75)		
	**AG**	22	8 (36.4)	14 (63.6)			20	7 (35)	13 (65)		
	**A**	27	10 (37)	17 (63)	-	0.510	24	8 (33.3)	16 (66.7)	-	0.151
	**G**	39	16 (41)	23 (59)	-	1*	32	14 (43.8)	18 (56.2)	-	0.625*
**rs3008**	**GG**	8	3 (37.5)	5 (62.5)	-	0.921*	6	1 (16.7)	5 (83.3)	-	0.452*
	**AA**	16	6 (37.5)	10 (62.5)			14	7 (50)	7 (50)		
	**AG**	20	9 (45)	11 (55)			16	7 (43.8)	9 (56.2)		
	**A**	36	15 (41.7)	21 (58.3)	-	1*	30	14 (46.7)	16 (53.3)	-	0.366*
	**G**	28	12 (42.9)	16 (57.1)	-	0.728	22	8 (36.4)	14 (63.6)	-	0.418
**rs3212752**	**TT**	37	15 (40.5)	22 (59.5)	-	1*	31	11 (35.5)	20 (64.5)	-	0.138*
	**CC**	0	-	-			0	-	-		
	**CT**	7	3 (42.9)	4 (57.1)			5	4 (80)	1 (20)		
	**C**	7	3 (42.9)	4 (57.1)	-	1*	5	4 (80)	1 (20)	-	0.138*
	**T**	44	-	-	-	-	36	-	-	-	-

EULAR: European League Against Rheumatism; OR: odds ratio; CI: confidence interval; NA: not available (indicates non-estimable values due to sparse data or quasi-complete separation); *: p value for Fisher's Exact Test

Table S38. Baricitinib LDA bivariate demographic and clinical analyses

Clinical variables	3 months						6 months					
	N	LDA		OR	$CI_{95\%}$	p-value	N	LDA		OR	$CI_{95\%}$	p-value
		LDA	No LDA					LDA	No LDA			
**Sex**												
**Woman**	39	4 (10.3)	35 (89.7)	-	-	0.469*	32	7 (21.9)	25 (78.1)	-	-	0.565*
**Man**	5	1 (20)	4 (80)				4	0 (0)	4 (100)			
**Smoking**												
**Smoker**	10	0 (0)	10 (100)	-	-	0.171*	8	1 (12.5)	7 (87.5)	-	-	0.378*
**Exsmoker**	7	2 (28.6)	5 (71.4)				7	0 (0)	7 (100)			
**No smoker**	27	3 (11.1)	24 (88.9)				21	6 (28.6)	15 (71.4)			
**Age at Dx**	44	43.6±11.4	42.5±11.2	-	-	0.852	36	43.7±7.1	43.5±12.3	-	-	0.971
**Years with RA**	44	11 (11-16)	13 (9.5-20)	-	-	0.931	36	14.8±9.1	14.4±7.6	-	-	0.915
**Years from Dx till JAK inhibitor treatment**	44	7.8 (5.4-11.1)	9.1 (5.8-15.1)	-	-	0.817	36	9.6 (7.8-10.4)	7.8 (5.1-14.6)	-	-	0.762
**JAK inhibitors start age**	44	57 (48-60)	55 (50.5-60.5)	-	-	0.947	36	54.7±8.9	53.6±10.1	-	-	0.795
**Treatment duration with JAK inhibitor (months)**	44	53.9±29.2	35.1±29.1	-	-	0.230	36	67.8 (34-77.7)	41.7 (13.9-58.5)	-	-	0.236
**Biologic-naïve**												
**Yes**	4	0 (0)	4 (100)	-	-	1*	4	2 (50)	2 (50)	-	-	0.162*
**No**	40	5 (12.5)	35 (87.5)				32	5 (15.6)	27 (84.4)			
**Number of previous BTs**	44	2 (1-2)	2 (1-4)	-	-	0.131	36	2 (1-2.5)	2 (1-4)	-	-	0.358
**Previous BTs duration (months)**	44	15.7 (13.4-152.3)	56.9 (16.3-127.9)	-	-	0.662	36	56.6 (14.1-217.9)	39.2 (15.3-126.3)	-	-	0.450
**BTs cause of suspensión**												
**Fallo primario**	10	1 (10)	9 (90)	-	-	0.218*	8	2 (25)	6 (75)	-	-	0.120*
**Fallo secundario**	22	2 (9.1)	20 (90.9)				17	2 (11.8)	15 (88.2)			
**Efecto adverso**	6	1 (16.7)	5 (83.3)				6	0 (0)	6 (100)			
**Toxicidad**	1	0 (0)	1 (100)				0	-	-			
**Otros**	1	1 (100)	0 (0)				1	1 (100)	0 (0)			
**Baseline RF (Cualitative)**												
**Pos**	38	5 (13.2)	33 (86.8)	-	-	0.345	30	7 (23.3)	23 (76.7)	-	-	0.317*
**Neg**	6	0 (0)	6 (100)				6	0 (0)	6 (100)			
**Baseline ACPA**												
**Pos**	37	4 (10.8)	33 (89.2)	-	-	0.790	30	6 (20)	24 (80)	-	-	1*
**Neg**	7	1 (14.3)	6 (85.7)				6	1 (16.7)	5 (83.3)			
**Baseline CCI**												
**Absence**	18	2 (11.1)	16 (88.9)	-	-	0.827*	15	3 (20)	12 (80)	-	-	1*
**Low**	18	3 (16.7)	15 (83.3)				14	3 (21.4)	11 (78.6)			
**High**	8	0 (0)	8 (100)				7	1 (14.3)	6 (85.7)			
**BMI**	44	24.6 (24.4-27.6)	26.4 (24.6-29.8)	-	-	0.713	36	24.6 (22.9-26.3)	26.5 (24.9-29.7)	0.76	0.52-1	0.062
**JAK inhibitor dose change**												
**Yes**	9	2 (22.2)	7 (77.8)	-	-	0.267*	9	3 (33.3)	6 (66.7)	-	-	0.332*
**No**	35	3 (8.6)	32 (91.4)				27	4 (14.8)	23 (85.2)			
**JAK inhibitor suspensión**												
**Yes**	21	2 (9.5)	19 (90.5)	-	-	1*	14	0 (0)	14 (100)	1	-	0.028*
**No**	23	3 (13)	20 (87)				22	7 (31.8)	15 (68.2)	1.47 × 10^8	NA-NA	

**JAK inhibitor cause of suspension**												
**Primary failure**	5	1 (20)	4 (80)	-	-	0.714*	2	0 (0)	2 (100)	-	-	0.199*
**Secondary failure**	10	1 (10)	9 (90)				8	0 (0)	8 (100)			
**Adverse events**	6	0 (0)	6 (100)				4	0 (0)	4 (100)			
**Others**	0	-	-				0	-	-			
**BT after JAK inhibitor treatment**												
**Yes**	17	2 (11.8)	15 (88.2)	-	-	1*	10	0 (0)	10 (100)	1	-	0.067
**No**	27	3 (11.1)	24 (88.9)				26	7 (26.9)	19 (73.1)	4.26×10^7	(NA-NA)	
**Adverse events to JAK inhibitor**												
**Yes**	16	1 (6.2)	15 (93.8)	-	-	0.638*	13	2 (15.4)	11 (84.6)	-	-	1*
**No**	28	4 (14.3)	24 (85.7)				23	5 (21.7)	18 (78.3)			
**Concomitant DMARDs**												
**MTX**	10	0 (0)	10 (100)	-	-	0.120*	7	1 (14.3)	6 (85.7)	-	-	0.851*
**HXQ**	1	1 (100)	0 (0)				1	0 (0)	1 (100)			
**SSZ**	0	-	-				0	-	-			
**LFN**	3	0 (0)	3 (100)				3	1 (33.3)	2 (66.7)			
**None**	30	4 (13.3)	26 (86.7)				25	5 (20)	20 (80)			
**Concomitant statins**												
**yes**	19	2 (10.5)	17 (89.5)	-	-	1*	19	4 (21.1)	15 (78.9)	-	-	1*
**No**	25	3 (12)	22 (88)				17	3 (17.6)	14 (82.4)			
**Concomitant GC**												
**yes**	31	4 (12.9)	27 (87.1)	-	-	1*	23	5 (21.7)	18 (78.3)	-	-	1*
**No**	13	1 (7.7)	12 (92.3)				13	2 (15.4)	11 (84.6)			
**Concomitant vitamin D**												
**Yes**	21	4 (19)	17 (81)	-	-	0.176*	18	4 (22.2)	14 (77.8)	-	-	1*
**No**	23	1 (4.3)	22 (95.7)				18	3 (16.7)	15 (83.3)			
**Baseline DAS28**	44	3.34±1.35	4.47±1.29	-	-	0.137	36	4.75±1.88	4.29±1.19	-	-	0.557
**Baseline TJC**	44	1 (0-2)	6 (3.5-9.5)	0.55	0.28-0.85	0.001	36	5 (3-8)	5 (3-8)	-	-	0.834
**Baseline SJC**	44	1 (0-2)	3 (2-4)	-	-	0.276	36	3 (0.5-6)	2 (0-4)	-	-	0.359
**Baseline PVAS**	44	2 (1-3)	7 (5-8)	0.64	0.40-0.93	0.077	36	4 (2.5-7.5)	7 (5-8)	-	-	0.281
**Baseline MVAS**	44	1 (1-5)	6 (4-7)	0.58	0.33-0.89	0.051	36	3 (2-6)	6 (5-7)	-	-	0.279
**Baseline CRP**	44	5.6 (1.3-7.6)	4.4 (1.8-8.9)	-	-	0.157	36	4.2 (1.4-4.9)	4.6 (1.5-8.7)	-	-	0.703
**Baseline ESR**	44	32 (9-43)	23 (8.5-45)	-	-	0.691	36	43 (9.5-59)	23 (9-36)	-	-	0.436
**Baseline RF (Quantitative)**	44	98 (33-110)	61 (24-162)	-	-	0.791	36	44 (30-107.5)	61 (18-160)	-	-	0.381
**Baseline TC**	44	207.4±61.2	195 ±47.9	-	-	0.682	36	206 (160-222)	202 (182-235)	-	-	0.576
**Baseline LDL**	44	134.2±39.8	118.5±33.8	-	-	0.442	36	139.5±45.6	117.5±30.4	-	-	0.262
**Baseline TG**	44	116 (92-118)	89 (72.5-118.5)	-	-	0.390	36	91 (70-133)	87 (71-132)	-	-	0.545

LDA: low disease activity; TJC: tender joints count; SJC: swallowed joint count; PVAS: patient visual analogue scale; MVAS: physician visual analogue scale; RF: rheumatoid factor; ACPA: anti-citrullinated protein antibodies; ESR: erythrocyte sedimentation rate; CRP: C-reactive protein; TC: total cholesterol; LDL: low-density lipoprotein; TG: triglycerides; BMI: body mass index; CCI: Charlson Comorbidity Index; JAK inhibitor: Janus kinase inhibitor; BTs: biologic therapies; GC: glucocorticoids; DMARDs: disease-modifying antirheumatic drugs; MTX: methotrexate; HXQ:, hydroxychloroquine; LFN, leflunomide; SSZ: sulfasalazine; OR, odds ratio; CI, confidence interval; NA: not available (indicates non-estimable values due to sparse data or quasi-complete separation); *: p value for Fisher's Exact Test

Table S39. Baricitinib LDA bivariate genetic analyses

SNPs	Genotipe	3 months					6 months				
		N	LDA		OR $CI_{95\%}$	p-value	N	LDA		OR $CI_{95\%}$	p-value
			LDA	No LDA				LDA	No LDA		
JAK1											
**rs2230587**	**GG**	30	2 (6.7)	28 (93.3)	-	0.249*	25	5 (20)	20 (80)	-	1*
	**AA**	1	0 (0)	1 (100)			1	0 (0)	1 (100)		
	**AG**	13	3 (23.1)	10 (76.9)			10	2 (20)	8 (80)		
	**A**	14	3 (21.4)	11 (78.6)	-	0.306*	11	2 (18.2)	9 (81.8)	-	1*
	**G**	43	5 (11.6)	38 (88.4)	-	1*	35	7 (20)	28 (80)	-	1*
**rs310241**	**GG**	5	0 (0)	5 (100)	-	0.807*	5	0 (0)	5 (100)	-	0.456*
	**AA**	25	4 (16)	21 (84)			18	3 (16.7)	15 (83.3)		
	**AG**	14	1 (7.1)	13 (92.9)			13	4 (30.8)	9 (69.2)		
	**A**	39	5 (12.8)	34 (87.2)	-	1*	31	7 (22.6)	24 (77.4)	-	0.559*
	**G**	19	1 (5.3)	18 (94.7)	-	0.370*	18	4 (22.2)	14 (77.8)	-	1*
**rs2230588**	**CC**	4	0 (0)	4 (100)	-	0.777*	4	0 (0)	4 (100)	-	0.590*
	**TT**	25	4 (16)	21 (84)			18	3 (16.7)	15 (83.3)		
	**CT**	15	1 (6.7)	14 (93.3)			14	4 (28.6)	10 (71.4)		
	**T**	40	5 (12.5)	35 (87.5)	-	1*	32	7 (21.9)	25 (78.1)	-	0.565*
	**C**	19	1 (5.3)	18 (94.7)	-	0.370*	18	4 (22.2)	14 (77.8)	-	1*
**rs10889504**	**GG**	32	2 (6.2)	30 (93.8)	-	0.199*	27	4 (14.8)	23 (85.2)	-	0.445*
	**CC**	1	0 (0)	1 (100)			1	0 (0)	1 (100)		
	**GC**	11	3 (27.3)	8 (72.7)			8	3 (37.5)	5 (62.5)		
	**C**	11	3 (27.3)	8 (72.7)	5.81 (0.83-50.28)	0.054	8	3 (37.5)	5 (62.5)	-	0.143
	**G**	43	5 (11.6)	38 (88.4)	-	1*	35	7 (20)	28 (80)	-	1*
**rs2780815**	**GG**	15	1 (6.7)	14 (93.3)	-	0.713*	15	4 (26.7)	11 (73.3)	-	0.468*
	**TT**	11	2 (18.2)	9 (81.8)			7	0 (0)	7 (100)		
	**GT**	18	2 (11.1)	16 (88.9)			14	3 (21.4)	11 (78.6)		
	**T**	29	4 (13.8)	25 (86.2)	-	0.646*	21	3 (14.3)	18 (85.7)	-	0.417*
	**G**	33	3 (9.1)	30 (90.9)	-	0.585*	29	7 (24.1)	22 (75.9)	-	0.302
JAK2											
**rs10119004**	**GG**	12	1 (8.3)	11 (91.7)	-	0.718*	11	3 (27.3)	8 (72.7)	-	0.670*
	**AA**	15	1 (6.7)	14 (93.3)			11	1 (9.1)	10 (90.9)		
	**AG**	17	3 (17.6)	14 (82.4)			14	3 (21.4)	11 (78.6)		
	**A**	32	4 (12.5)	28 (87.5)	-	1*	25	4 (16)	21 (84)	-	0.649*
	**G**	29	4 (13.8)	25 (86.2)	-	0.646*	25	6 (24)	19 (76)	-	0.399*
**rs7857730**	**GG**	8	0 (0)	8 (100)	-	0.827*	7	1 (14.3)	6 (85.7)	-	1*
	**TT**	18	2 (11.1)	16 (88.9)			14	3 (21.4)	11 (78.6)		
	**GT**	18	3 (16.7)	15 (83.3)			15	3 (20)	12 (80)		
	**G**	26	3 (11.5)	23 (88.5)	-	1*	22	4 (18.2)	18 (81.8)	-	1*
	**T**	36	5 (13.9)	31 (86.1)	-	0.566*	29	6 (20.7)	23 (79.3)	-	0.700
**rs2274472**	**CC**	3	0 (0)	3 (100)	-	1*	2	2 (100)	0 (0)	1	0.029
	**TT**	17	2 (11.8)	15 (88.2)			14	3 (21.4)	11 (78.6)	2×10^{-9} (NA-NA)	
	**CT**	24	3 (12.5)	21 (87.5)			20	2 (10)	18 (90)	6×10^{-9} (NA-NA)	
	**C**	27	3 (11.1)	24 (88.9)	-	1*	22	4 (18.2)	18 (81.8)	-	1*
	**T**	41	5 (12.2)	36 (87.8)	-	1*	34	5 (14.7)	29 (85.3)	4×10^{-9} (NA-NA)	0.033*
**rs2230722**	**CC**	27	2 (7.4)	25 (92.6)	-	0.474*	24	4 (16.7)	20 (83.3)	-	0.720*
	**TT**	2	0 (0)	2 (100)			1	0 (0)	1 (100)		
	**CT**	15	3 (20)	12 (80)			11	3 (27.3)	8 (72.7)		
	**C**	42	5 (11.9)	37 (88.1)	-	1*	35	7 (20)	28 (80)	-	1*
	**T**	17	3 (17.6)	14 (82.4)	-	0.359*	12	3 (25)	9 (75)	-	0.663*
**rs2230724**	**GG**	10	0 (0)	10 (100)	-	0.425*	9	2 (22.2)	7 (77.8)	-	0.877*
	**AA**	17	2 (11.8)	15 (88.2)			13	3 (23.1)	10 (76.9)		
	**AG**	17	3 (17.6)	14 (82.4)			14	2 (14.3)	12 (85.7)		
	**A**	34	5 (14.7)	29 (85.3)	-	0.573*	27	5 (18.5)	22 (81.5)	-	0.807
	**G**	27	3 (11.1)	24 (88.9)	-	1*	23	4 (17.4)	19 (82.6)	-	0.685*
JAK3											
**rs3212780**	**GG**	17	3 (17.6)	14 (82.4)	-	0.807*	12	3 (25)	9 (75)	-	0.846*
	**AA**	5	0 (0)	5 (100)			4	0 (0)	4 (100)		
	**AG**	22	2 (9.1)	20 (90.9)			20	4 (20)	16 (80)		
	**A**	27	2 (7.4)	25 (92.6)	-	0.359*	24	4 (16.7)	20 (83.3)	-	0.663*
	**G**	39	5 (12.8)	34 (87.2)	-	1*	32	7 (21.9)	25 (78.1)	-	0.565*
**rs3008**	**GG**	8	3 (37.5)	5 (62.5)	1.89×10^8 (NA-NA)	0.022*	6	1 (16.7)	5 (83.3)	-	1*
	**AA**	16	0 (0)	16 (100)	1		14	3 (21.4)	11 (78.6)		
	**AG**	20	2 (10)	18 (90)	3.49×10^7 (NA-NA)		16	3 (18.8)	13 (81.2)		

	A	36	2 (5.6)	34 (94.4)	0.10 (0.01-0.72)	0.034	30	6 (20)	24 (80)	-	1*
	G	28	5 (17.9)	23 (82.1)	6.83×10^7 (NA-NA)	0.072	22	4 (18.2)	18 (81.8)	-	1*
rs3212752	TT	37	4 (10.8)	33 (89.2)	-	1*	31	5 (16.1)	26 (83.9)	-	0.244*
	CC	0	-	-			0	-	-		
	CT	7	1 (14.3)	6 (85.7)			5	2 (40)	3 (60)		
	C	7	1 (14.3)	6 (85.7)	-	1*	5	2 (40)	3 (60)	-	0.244*
	T	44	-	-	-	-	36	-	-	-	-

LDA: Low disease activity; OR: odds ratio; CI: confidence interval; NA: not available (indicates non-estimable values due to sparse data or quasi-complete separation); *: p value for Fisher's Exact Test

Table S40. Baricitinib remission bivariate demographic and clinical analyses

Clinical variables	3 months						6 months					
	N	Remission		OR	$CI_{95\%}$	p-value	N	Remission		OR	$CI_{95\%}$	p-value
		Remission	No Remission					Remission	No Remission			
**Sex**												
**Woman**	39	13 (33.3)	26 (66.7)	-	-	0.300	32	12 (37.5)	20 (62.5)	-	-	0.278*
**Man**	5	0 (0)	5 (100)				4	0 (0)	4 (100)			
**Smoking**												
**Smoker**	10	4 (40)	6 (60)	-	-	0.516	8	3 (37.5)	5 (62.5)	-	-	0.619*
**Exsmoker**	7	1 (14.3)	6 (85.7)				7	1 (14.3)	6 (85.7)			
**No smoker**	27	8 (29.6)	19 (70.4)				21	8 (38.1)	13 (61.9)			
**Age at Dx**	44	41.1±14.2	43.3±9.8	-	-	0.610	36	39.3±13.4	45.7±9.9	-	-	0.161
**Years with RA**	44	17±7.6	14.5±8	-	-	0.357	36	14.8±9.4	14.3±7	-	-	0.883
**Years from Dx till JAK inhibitor treatment**	44	10.4 (8.7-15.4)	7.8 (5.2-14.7)	-	-	0.321	36	9.7 (4.9-14.4)	7.7 (5.3-13.0)	-	-	0.614
**JAKi start age**	44	54 (53-60)	55 (47.5-60.5)	-	-	0.949	36	50.5 ± 9.6	55.5±9.6	-	-	0.149
**Treatment duration with JAK inhibitor (months)**	44	50.5±31.2	31.6±27.1	1.02	1.00-1.05	0.071	36	44.6 (17.7-75.1)	44.9 (12.3-59.5)	-	-	0.555
**Biologic-naïve**												
**Yes**	4	4 (100)	0 (0)	1.46×10^8	NA-NA	0.005	4	3 (75)	1 (25)	7.66	0.86-166.87	0.060
**No**	40	9 (22.5)	31 (77.5)	1	-		32	9 (28.1)	23 (71.9)	1	-	
**Number of previous BTs**	44	2 (0-3)	2 (1-4)	-	-	0.502	36	2(0.7-2.5)	2.5 (1-4)	-	-	0.525
**Previous BTs duration (months)**	44	113.9 (14.1-154.2)	56.6 (15.3-127.9)	-	-	0.691	36	17.8 (14.1-154.2)	56.6 (15.3-126.3)	-	-	0.958
**BTs cause of suspension**												
**Primary failure**	10	2 (20)	8 (80)	-	-	1*	8	3 (37.5)	5 (62.5)	-	-	0.907*
**Secondary failure**	22	6 (27.3)	16 (72.7)				17	5 (29.4)	12 (70.6)			
**Adverse events**	6	1 (16.7)	5 (83.3)				6	1 (16.7)	5 (83.3)			
**Toxicity**	1	0 (0)	1 (100)				0	-	-			
**Others**	1	0 (0)	1 (100)				1	0 (0)	1 (100)			
**Baseline RF (Cualitative)**												
**Pos**	38	11 (28.9)	27 (71.1)	-	-	1*	30	10 (33.3)	20 (66.7)	-	-	1*
**Neg**	6	2 (33.3)	4 (66.7)				6	2 (33.3)	4 (66.7)			
**Baseline ACPA**												
**Pos**	37	11 (29.7)	26 (70.3)	-	-	1*	30	10 (33.3)	20 (66.7)	-	-	1*
**Neg**	7	2 (28.6)	5 (71.4)				6	2 (33.3)	4 (66.7)			
**Baseline CCI**												
**Absence**	18	6 (33.3)	12 (66.7)	-	-	0.891	15	7 (46.7)	8 (53.3)	-	-	0.312*
**Low**	18	5 (27.8)	13 (72.2)				14	4 (28.6)	10 (71.4)			
**High**	8	2 (25)	6 (75)				7	1 (14.3)	6 (85.7)			
**BMI**	44	26.1 (23.8-28.1)	26.5 (24.7-29.8)	-	-	0.358	36	25.6 (23.2-26.4)	27.2 (24.9-29.9)	0.76	0.56-0.96	0.034
**JAK inhibitor dose change**												
**Yes**	9	5 (55.6)	4 (44.4)	4.21	0.91-20.99	0.055	9	4 (44.4)	5 (55.6)	-	-	0.414
**No**	35	8 (22.9)	27 (77.1)	1	-		27	8 (29.6)	19 (70.4)			
**JAK inhibitor suspensión**												
**Yes**	21	3 (14.3)	18 (85.7)	1	-	0.034	14	2 (14.3)	12 (85.7)	1	-	0.053
**No**	23	10 (43.5)	13 (56.5)	4.61	1.15-23.75		22	10 (45.5)	12 (54.5)	5	1.04-37.25	
**JAK inhibitor cause of suspension**												
**Primary failure**	5	0 (0)	5 (100)	-	-	0.774	2	0 (0)	2 (100)	-	-	1*
**Secondary failure**	10	2 (20)	8 (80)				8	1 (12.5)	7 (87.5)			
**Adverse events**	6	1 (16.7)	5 (83.3)				4	1 (25)	3 (75)			
**Others**	0	-	-				0	-	-			
**BTs after JAK inhibitor treatment**												
**Yes**	17	1 (5.9)	16 (94.1)	1	-	0.006	10	1 (10)	9 (90)	1	-	0.065
**No**	27	12 (44.4)	15 (55.6)	12.80	2.12-247.80		26	11 (42.3)	15 (57.7)	6.60	1.01-131.0	
**Adverse events to JAK inhibitors**												
**Yes**	16	6 (37.5)	10 (62.5)	-	-	0.382	13	6 (46.2)	7 (53.8)	-	-	0.219
**No**	28	7 (25)	21 (75)				23	6 (26.1)	17 (73.9)			
**Concomitant DMARDs**												
**MTX**	10	5 (50)	5 (50)	-	-	0.356	7	5 (71.4)	2 (28.6)	3.91×10^7	NA-NA	0.092
**HXQ**	1	0 (0)	1 (100)				1	0 (0)	1 (100)	1	-	
**SSZ**	0	-	-				0	-	-	-	-	
**LFN**	3	1 (33.3)	2 (66.7)				3	1 (33.3)	2 (66.7)	7.83×10^6	NA-NA	
**None**	30	7 (23.3)	23 (76.7)				25	6 (24)	19(76)	4.92×10^6	NA-NA	
**Concomitant statins**												
**Yes**	19	7 (36.8)	12 (63.2)	-	-	0.355	19	5 (26.3)	14 (73.7)	-	-	0.345
**No**	25	6 (24)	19 (76)				17	7 (41.2)	10 (58.8)			
**Concomitant GC**												
**Yes**	31	4 (12.9)	27 (87.1)	1	-	<0.001	23	3 (13)	20 (87)	1	-	<0.001
**No**	13	9 (69.2)	4 (30.8)	15.18	3.42-84.26		13	9 (69.2)	4 (30.8)	15.00	3.08-96.56	
**Concomitant vitamin D**												
**Yes**	21	4 (19)	17 (81)	-	-	0.144	18	4 (22.2)	14 (77.8)	-	-	0.157
**No**	23	9 (39.1)	14 (60.9)				18	8 (44.4)	10 (55.6)			
**Baseline DAS28**	44	4.19±1.65	4.40±1.21	-	-	0.682	36	4.39±1.69	4.38±1.14	-	-	0.977
**Baseline TJC**	44	5 (3-10)	5 (3-8)	-	-	0.663	36	5 (3.8-7.8)	5 (2.8-8.3)	-	-	0.847
**Baseline SJC**	44	3 (1-4)	2 (0-4)	-	-	0.459	36	3 (0.7-4.3)	2 (0-4)	-	-	0.328
**Baseline PVAS**	44	7 (3-8)	7 (4-8)	-	-	0.893	36	7 (4.5-8)	6.5 (3.8-8)	-	-	0.618
**Baseline MVAS**	44	5 (3-7)	5 (3.5-7)	-	-	0.679	36	6 (4.5-7)	5 (3.8-7)	-	-	0.388
**Baseline CRP**	44	4.3 (2.2-8.8)	4.6 (1.3-8.8)	-	-	0.587	36	4.3 (2.7-9.8)	3.8 (1.3-7.9)	-	-	0.304
**Baseline ESR**	44	10 (8-33)	30 (9.5-45)	-	-	0.343	36	10 (8.8-38.3)	30 (12.8-44)	-	-	0.414
**Baseline RF (Quantitative)**	44	69 (40-105)	61 (23.5-186.5)	1	0.99-1.01	0.085	36	42 (23-95.2)	61 (21.8-161.2)	-	-	0.106
**Baseline TC**	44	203.6±34.5	193.3±54	-	-	0.453	36	188 (177.2-211.7)	208.5 (181.7-240.7)	-	-	0.350
**Baseline LDL**	44	124.7±36.5	118.5±33.9	-	-	0.602	36	119±37	123.2±33.5	-	-	0.741
**Baseline TG**	44	85 (68-106)	92 (76.5-119)	-	-	0.525	36	79.5 (54.5-101.2)	105.5 (77.2-141.2)	-	-	0.264

TJC: tender joints count; SJC: swallowed joint count; PVAS: patient visual analogue scale; MVAS: physician visual analogue scale; RF: rheumatoid factor; ACPA: anti-citrullinated protein antibodies; ESR: erythrocyte sedimentation rate; CRP: C-reactive protein; TC: total cholesterol; LDL: low-density lipoprotein; TG: triglycerides; BMI: body mass index; CCI: Charlson Comorbidity Index; JAK inhibitor: Janus kinase inhibitor; BTs: biologic therapies; GC: glucocorticoids; DMARDs: disease-modifying antirheumatic drugs; MTX: methotrexate; HXQ:, hydroxychloroquine; LFN, leflunomide; SSZ: sulfasalazine; OR, odds ratio; CI, confidence interval; NA: not available (indicates non-estimable values due to sparse data or quasi-complete separation); *: p value for Fisher's Exact Test

Table S41. Baricitinib remission bivariate genetic analyses

SNPs	Genotipe	3 months					6 months				
		N	Remission		OR $CI_{95\%}$	p-value	N	Remission		OR $CI_{95\%}$	p-value
			Remission	No Remission				Remission	No Remission		
JAK1											
rs2230587	GG	30	8 (26.7)	22 (73.3)	-	0.410*	25	9 (36)	16 (64)	-	1*

	**AA**	1	1 (100)	0 (0)			1	0 (0)	1 (100)		
	**AG**	13	4 (30.8)	9 (69.2)			10	3 (30)	7 (70)		
	**A**	14	5 (35.7)	9 (64.3)	-	0.540	11	3 (27.3)	8 (72.7)	-	0.608
	**G**	43	12 (27.9)	31 (72.1)	-	0.295*	35	12 (34.3)	23 (65.7)	-	1*
**rs310241**	**GG**	5	1 (20)	4 (80)			5	0 (0)	5 (100)	1	
	**AA**	25	9 (36)	16 (64)	-	0.634*	18	9 (50)	9 (50)	1.16×10^8 (NA-NA)	0.068
	**AG**	14	3 (21.4)	11 (78.6)			13	3 (23.1)	10 (76.9)	3.47×10^7 (NA-NA)	
	**A**	39	12 (30.8)	27 (69.2)	-	1*	31	12 (38.7)	19 (61.3)	2.69×10^7 (NA-NA)	0.088
	**G**	19	4 (21.1)	15 (78.9)	-	0.281	18	3 (16.7)	15 (83.3)	0.20 (0.04-0.87)	0.033
**rs2230588**	**CC**	4	1 (25)	3 (75)			4	0 (0)	4 (100)		
	**TT**	25	9 (36)	16 (64)	-	0.699*	18	9 (50)	9 (50)	-	0.100*
	**CT**	15	3 (20)	12 (80)			14	3 (21.4)	11 (78.6)		
	**T**	40	12 (30)	28 (70)	-	1*	32	12 (37.5)	20 (62.5)	-	0.278*
	**C**	19	4 (21.1)	15 (78.9)	-	0.281	18	3 (16.7)	15 (83.3)	0.20 (0.04-0.87)	0.033
**rs10889504**	**GG**	32	7 (21.9)	25 (78.1)	1		27	8 (29.6)	19 (70.4)		
	**CC**	1	1 (100)	0 (0)	NA	0.098	1	0 (0)	1 (100)	-	0.601*
	**GC**	11	5 (45.5)	6 (54.5)	2.98 (0.68-13.11)		8	4 (50)	4 (50)		
	**C**	11	5 (45.5)	6 (54.5)	-	0.181	8	4 (50)	4 (50)	-	0.256
	**G**	43	12 (27.9)	31 (72.1)	-	0.295*	35	12 (34.3)	23 (65.7)	-	1*
**rs2780815**	**GG**	15	5 (33.3)	10 (66.7)			15	3 (20)	12 (80)	1	
	**TT**	11	4 (36.4)	7 (63.6)	-	0.704*	7	5 (71.4)	2 (28.6)	10 (1.43-101.76)	0.051
	**GT**	18	4 (22.2)	14 (77.8)			14	4 (28.6)	10 (71.4)	1.60 (0.28-9.81)	
	**T**	29	8 (27.6)	21 (72.4)	-	0.692	21	9 (42.9)	12 (57.1)	-	0.151
	**G**	33	9 (27.3)	24 (72.7)	-	0.567	29	7 (24.1)	22 (75.9)	0.13 (0.02-0.73)	0.017
JAK2											
**rs10119004**	**GG**	12	5 (41.7)	7 (58.3)			11	4 (36.4)	7 (63.6)		
	**AA**	15	3 (20)	12 (80)	-	0.500*	11	3 (27.3)	8 (72.7)	-	1*
	**AG**	17	5 (29.4)	12 (70.6)			14	5 (35.7)	9 (64.3)		
	**A**	32	8 (25)	24 (75)	-	0.280	25	8 (32)	17 (68)	-	0.798
	**G**	29	10 (34.5)	19 (65.5)	-	0.318	25	9 (36.0)	16 (64)	-	0.608
**rs7857730**	**GG**	8	2 (25)	6 (75)			7	3 (42.9)	4 (57.1)		
	**TT**	18	4 (22.2)	14 (77.8)	-	0.522	14	5 (35.7)	9 (64.3)	-	0.731*
	**GT**	18	7 (38.9)	11 (61.1)			15	4 (26.7)	11 (73.3)		
	**G**	26	9 (34.6)	17 (65.4)	-	0.375	22	7 (31.8)	15 (68.2)	-	0.809
	**T**	36	11 (30.6)	25 (69.4)	-	0.755	29	9 (31)	20 (69)	-	0.663*
**rs2274472**	**CC**	3	0 (0)	3 (100)			2	0 (0)	2 (100)		
	**TT**	17	7 (41.2)	10 (58.8)	-	0.397*	14	6 (42.9)	8 (57.1)	-	0.527*
	**CT**	24	6 (25)	18 (75)			20	6 (30)	14 (70)		
	**C**	27	6 (22.2)	21 (77.8)	-	0.179	22	6 (27.3)	16 (72.7)	-	0.333
	**T**	41	13 (31.7)	28 (68.3)	-	0.543*	34	12 (35.3)	22 (64.7)	-	0.542
**rs2230722**	**CC**	27	9 (33.3)	18 (66.7)			24	9 (37.5)	15 (62.5)		
	**TT**	2	0 (0)	2 (100)	-	0.871*	1	0 (0)	1 (100)	-	0.806*
	**CT**	15	4 (26.7)	11 (73.3)			11	3 (27.3)	8 (72.7)		
	**C**	42	13 (31)	29 (69)	-	1*	35	12 (34.3)	23 (65.7)	-	1*
	**T**	17	4 (23.5)	13 (76.5)	-	0.487	12	3 (25)	9 (75)	-	0.453
**rs2230724**	**GG**	10	3 (30)	7 (70)			9	3 (33.3)	6 (66.7)		
	**AA**	17	4 (23.5)	13 (76.5)	-	0.753	13	5 (38.5)	8 (61.5)	-	0.903*
	**AG**	17	6 (35.3)	11 (64.7)			14	4 (28.6)	10 (71.4)		
	**A**	34	10 (29.4)	24 (70.6)	-	1*	27	9 (33.3)	18 (66.7)	-	1*
	**G**	27	9 (33.3)	18 (66.7)	-	0.487	23	7 (30.4)	16 (69.6)	-	0.623
JAK3											
**rs3212780**	**GG**	17	5 (29.4)	12 (70.6)			12	6 (50)	6 (50)		
	**AA**	5	2 (40)	3 (60)	-	0.898*	4	1 (25)	3 (75)	-	0.359*
	**AG**	22	6 (27.3)	16 (72.7)			20	5 (25)	15 (75)		
	**A**	27	8 (29.6)	19 (70.4)	-	0.987	24	6 (25)	18 (75)	-	0.133
	**G**	39	11 (28.2)	28 (71.8)	-	0.623*	32	11 (34.4)	21 (65.6)	-	1*
**rs3008**	**GG**	8	0 (0)	8 (100)			6	0 (0)	6 (100)		
	**AA**	16	6 (37.5)	10 (62.5)	-	0.131*	14	6 (42.9)	8 (57.1)	-	0.190*
	**AG**	20	7 (35)	13 (65)			16	6 (37.5)	10 (62.5)		
	**A**	36	13 (36.1)	23 (63.9)	2×10^{-8} (NA-NA)	0.042	30	12 (40)	18 (60)	7.71×10^7 (NA-NA)	0.057
	**G**	28	7 (25)	21 (75)	-	0.382	22	6 (27.3)	16 (72.7)	-	0.333
**rs3212752**	**TT**	37	11 (29.7)	26 (70.3)			31	10 (32.3)	21 (67.7)		
	**CC**	0	-	-	-	1*	0	-	-	-	1*
	**CT**	7	2 (28.6)	5 (71.4)			5	2 (40)	3 (60)		
	**C**	7	2 (28.6)	5 (71.4)	-	1*	5	2 (40)	3 (60)	-	1*
	**T**	44	-	-	-	-	36	-	-	-	-

CI: confidence interval; NA: not available (indicates non-estimable values due to sparse data or quasi-complete separation); *: p value for Fisher's Exact Test
