# Supplementary material for: Clinical and Pharmacogenetic Factors Associated with Response to JAK Inhibitors in Patients with Rheumatoid Arthritis: A Real-World Study of JAK1, JAK2, and JAK3 Gene Variants
Source: Pharmaceutics. 2026 Jul 11;18(7):846. doi: 10.3390/pharmaceutics18070846 (PMC13415438; doi:10.3390/pharmaceutics18070846)
Supplement: Supplementary file 1 [file pharmaceutics-18-00846-s001.zip › Table S4. Minor allele frequency of SNPs.pdf]

Table S2. Minor allele frequencies of SNPs.						
**Drug**	**Chr**	**SNP**	**Minor allele**	**Major allele**	**MAF**	**NCHROBS**
**Tofacitinib**	**1**	rs2230587	A	G	0.09	100
		rs310241	G	A	0.22	100
		rs2230588	C	T	0.22	100
		rs10889504	C	G	0.1	100
		rs2780815	G	T	0.46	100
	**9**	rs10119004	A	G	0.47	100
		rs7857730	G	T	0.50	100
		rs2274472	C	T	0.47	100
		rs2230722	T	C	0.21	100
		rs2230724	G	A	0.45	100
	**19**	rs3212780	A	G	0.28	100
		rs3008	A	G	0.46	100
		rs3212752	C	T	0.06	100
**Baricitinib**	**1**	rs2230587	A	G	0.17	88
		rs310241	G	A	0.27	88
		rs2230588	C	T	0.26	88
		rs10889504	C	G	0.14	88
		rs2780815	G	T	0.45	88
	**9**	rs10119004	A	G	0.46	88
		rs7857730	G	T	0.38	88
		rs2274472	C	T	0.34	88
		rs2230722	T	C	0.21	88
		rs2230724	G	A	0.42	88
	**19**	rs3212780	A	G	0.36	88
		rs3008	A	G	0.40	88
		rs3212752	C	T	0.07	88
**Filgotinib**	**1**	rs2230587	A	G	0.12	40
		rs310241	G	A	0.20	40
		rs2230588	C	T	0.20	40
		rs10889504	C	G	0.07	40
		rs2780815	G	T	0.40	40

	9	rs10119004	A	G	0.47	40
		rs7857730	G	T	0.45	40
		rs2274472	C	T	0.45	40
		rs2230722	T	C	0.30	40
		rs2230724	G	A	0.47	40
	19	rs3212780	A	G	0.32	40
		rs3008	A	G	0.40	40
		rs3212752	C	T	0.02	40
**Upadacitinib**	1	rs2230587	A	G	0.13	72
		rs310241	G	A	0.25	72
		rs2230588	C	T	0.20	72
		rs10889504	C	G	0.13	72
		rs2780815	G	T	0.40	72
	9	rs10119004	A	G	0.50	72
		rs7857730	G	T	0.45	72
		rs2274472	C	T	0.44	72
		rs2230722	T	C	0.25	72
		rs2230724	G	A	0.48	72
	19	rs3212780	A	G	0.23	72
		rs3008	A	G	0.45	72
		rs3212752	C	T	0.05	72

Chr: Chromosome MAF: Minor allele frequency; NCHROBS: Number of Chromosome Observations; SNPs: single nucleotide polymorphisms
