# Supplementary material for: Clinical and Pharmacogenetic Factors Associated with Response to JAK Inhibitors in Patients with Rheumatoid Arthritis: A Real-World Study of JAK1, JAK2, and JAK3 Gene Variants
Source: Pharmaceutics. 2026 Jul 11;18(7):846. doi: 10.3390/pharmaceutics18070846 (PMC13415438; doi:10.3390/pharmaceutics18070846)
Supplement: Supplementary file 1 [file pharmaceutics-18-00846-s001.zip › Table S42-S47. Predictors of EULAR response, LDA and remission at 3 and 6 months in RA patients filgotinib (Bivariate analyisis).pdf]

Table S42. Filgotinib EULAR response bivariate demographic and clinical analyses

Clinical variables	3 months						6 months					
	N	Respuesta EULAR		p-value	OR	CI$_{95\%}$	N	Respuesta EULAR		p-value	OR	CI$_{95\%}$
		Satisfactory	Unsatisfactory					Satisfactory	Unsatisfactory			
**Sex**												
Woman	18	6 (33.3)	12 (66.7)	1*	-	-	15	6 (40)	9 (60)	**1**	**-**	**-**
Man	2	1 (50)	1 (50)				2	1 (50)	1 (50)			
**Smoking**												
Smoker	2	0 (0)	2 (100)	0.774	-	-	1	0 (0)	1 (100)	1	-	-
Exsmoker	2	1 (50)	1 (50)				2	1 (50)	1 (50)			
No smoker	16	6 (37.5)	10 (62.5)				14	6 (42.9)	8 (57.1)			
**Age at Dx**	20	34.2 ± 8.4	42.2 ± 11.2	0.127	-	-	17	40 ± 10.6	37.2 ± 10.2	0.606	-	-
**Years with RA**	20	14.8 ± 10.8	12.3 ± 6.1	0.486	-	-	17	13.6 ± 10	12.5 ± 5.8	0.797	-	-
**Years from Dx till JAK inhibitor treatment**	20	11.7 ± 10.6	10.1 ± 6.6	0.656	-	-	17	13.6 ± 10	12.5 ± 5.8	0.793	-	-
**JAK inhibitor start age**	20	46.1 ± 9.3	52.6 ± 9.5	0.167	-	-	17	51 ± 5.7	48.1 ± 12.8	0.597	-	-
**Treatment duration with JAK inhibitor (months)**	20	29 [17.5-36.3]	8.2 [6.6-19.3]	0.046*	0.91	[0.83-0.99]	17	27.6 [11.6 -36.2]	10.6 [7.5-15.9]	0.070	-	-
**Number of previous BTs**	20	1.2 ± 0.9	3 ± 1.7	0.010*	2.38	[1.10- 7.20]	17	2 ± 1.4	2.7 ± 1.8	0.399	-	-
**Previous BTs duration (months)**	20	5 [3-34]	52 [22-84]	0.157	-	-	17	20 [6.7-53.7]	51 [14.5-70]	0.875	-	-
**BTs cause of suspensión**												
**Primary failure**	7	3 (42.9)	4 (57.1)	0.651	-	-	5	4 (80)	1 (20)	0.338	-	-
**Secondary failure**	10	3 (30)	7 (70)	1*	-	-	9	5 (55.6)	4 (44.4)	1	-	-
**Adverse events**	3	1 (33.3)	2 (66.7)	1*	-	-	3	1 (33.3)	2 (66.7)	0.536	-	-
**Toxicity**	-	-	-	-	-	-	–	-	-	-	-	-
**Others**	-	-	-	-	-	-	-	-	-	-	-	-
**Baseline RF (Cualitative)**												
**Pos**	14	3 (21.4)	11 (78.6)	0.121	-	-	8	3 (37.5)	5 (62.5)	0.153	-	-
**Neg**	6	4 (66.7)	2 (33.3)				9	7 (77.8)	2 (22.2)			
**Baseline ACPA**												
**Pos**	18	6 (33.3)	12 (66.7)	1	-	-	15	8 (53.3)	7 (46.7)	0.485	-	-
**Neg**	2	1 (50)	1 (50)				2	2 (100)	0 (0)			
**Baseline CCI**												
**Absence**	13	5 (38.5)	8 (61.5)	1	-	-	12	7 (58.3)	5 (41.7)	1	-	-
**Low**	6	2 (33.3)	4 (66.7)				4	2 (50)	2 (50)			
**High**	1	0	1 (100)				1	1 (100)	0 (0)			
**BMI**	20	28.8 ± 4.6	29.7 ± 5.6	0.725	-	-	17	28.4 ± 4.1	30.2 ± 4.9	0.466	-	-
**JAK inhibitor dose change**												
**No**	18	5 (27.8)	13 (72.2)	0.110	-	-	15	8 (53.3)	7 (46.7)	0.485	-	-
**Yes**	2	2 (100)	0 (0)				2	2 (100)	0 (0)			
**JAK inhibitor suspensión**												
**No**	13	6 (46.2)	7 (53.8)	0.328	-	-	13	9 (69.2)	4 (30.8)	0.25	-	-
**Yes**	7	1 (14.3)	6 (85.7)				4	1 (25)	3 (75)			
**JAK inhibitor cause of suspension**												
**Primary failure**	2	0 (0)	2 (100)	0.571	-	-	1	0 (0)	1 (100)	1	-	-
**Secondary failure**	2	1 (50)	1 (50)				1	0 (0)	1 (100)			
**Adverse events**	3	0 (0)	3 (100)				2	1 (50)	1 (50)			
**Others**	0	0 (0)	0 (0)				0	0 (0)	0 (0)			
**BT after JAK inhibitor treatment**												
No	13	6 (46.2)	7 (53.8)	0.040	2×10^{-9}	[NA -Inf]	13	10 (76.9)	3 (23.1)	0.010	3.85×10^{8}	[NA-NA]
Yes	7	7 (100)	0 (0)				4	0 (0)	4 (100)			
**Adverse events to JAK inhibitors**												
Yes	7	2 (28.6)	5 (71.4)	1	-	-	6	5 (83.3)	1 (16.7)	0.304	-	-
No	13	5 (38.5)	8 (61.5)				11	5 (45.5)	6 (54.4)			
**Concomitant DMARDs**												
MTX	7	1 (!4.3)	6 (85.7)	0.229	-	-	5	2 (40)	3 (60)	0.762	-	-
HXQ	1	0 (0)	1 (100)				1	1 (100)	0 (0)			
SSZ	0	0	0				0	0	0			
LFN	0	0	0				0	0	0			

None	12	6 (50)	6 (50)				11	7 (63.6)	4 (36.4)			
**Concomitant statins**												
no	15	6 (40)	9 (60)	0.612	-	-	12	7 (58.3)	5 (41.7)	1	-	-
yes	5	1 (20)	4 (80)				5	3 (60)	2 (40)			
**Concomitant GC**												
No	10	5 (50)	5 (50)	0.349	-	-	9	6 (66.7)	3 (33.3)	0.637	-	-
yes	10	2 (20)	8 (80)				8	4 (50)	4 (50)			
**Concomitant vitamin D**												
No	13	6 (46.2)	7 (53.8)	0.328	-	-	11	8 (72.7)	3 (27.3)	0.161	-	-
yes	7	1 (14.3)	6 (85.7)				6	2 (33.3)	4 (66.7)			
**Baseline DAS28**	20	4.9 [2.4-5.5]	5.2 [4.6-5.2]	0.263	-	-	17	2.1 ± 0.7	4.3 ± 0.8	<0.001*	1.86×10^{89}	[0 - NA]
**Baseline TJC**	20	0 [0-1.5]	6 [4-8]	<0.001	0.28	[0.05- 0.65]	17	1.5 [0-2]	7 [6-9.5]	0.003	2.13×10^{18}	[0 - NA]
**Baseline SJC**	20	0 [0-0]	3 [1-4]	0.002	1.28×10^{8}	[2.97×10^{-169}- NA]	17	0 [0-0]	3 [1.5-3.5]	0.010	8.46	[0 - NA]
**Baseline PVAS**	20	3.2 ± 1.8	6.1 ± 1.9	0.007	2.31	[1.25-6.95]	17	3.4 ± 1.9	6 ± 1.5	0.007	2.65	[1.26 -9.71]
**Baseline MVAS**	20	3.1 ± 1.8	5.3 ± 2.1	0.027	1.76	[1.07-3.45]	17	2.6 ± 1.8	5.7 ± 1.9	0.006	2.27	[1.25-6.37]
**Baseline CRP**	20	1.9 [1.2-2.8]	1.9 [0.5-9]	0.160	-	-	17	1.7 [0.7-2.4]	3.1 [1.7-7]	0.365	-	-
**Baseline ESR**	20	17 [6.5-20]	14 [9-21]	0.609	-	-	17	9.5 [5.8-16.2]	11 [8-30]	0.315	-	-
**Baseline RF (Quantitative)**	20	15 [7-20]	61 [20-110]	0.742	-	-	17	11.5 [10-18.2]	50 [15-107]	0.456	-	-
**Baseline TC**	20	200.7 ± 40.9	215.3 ± 33.5	0.437	-	-	17	215 ± 32.9	196.4 ± 38.8	0.323	-	-
**Baseline LDL**	20	119.3 ± 30.3	116.7 ± 21	0.844	-	-	17	130.9 ± 24.4	103.5 ± 24.2	0.030	0.95	[0.80- 0.99]
**Baseline TG**	20	65 [56-113]	84 [66-98]	0.822	-	-	17	84.9 ± 38.4	78.4 ± 24.6	0.678	-	-

EULAR: European League Against Rheumatism; TJC: tender joints count; SJC: swallowed joint count; PVAS: patient visual analogue scale; MVAS: physician visual analogue scale; RF: rheumatoid factor; ACPA: anti-citrullinated protein antibodies; ESR: erythrocyte sedimentation rate; CRP: C-reactive protein; TC: total cholesterol; LDL: low-density lipoprotein; TG: triglycerides; BMI: body mass index; CCI: Charlson Comorbidity Index; JAK inhibitor: Janus kinase inhibitor; BTs: biologic therapies; GC: glucocorticoids; DMARDs: disease-modifying antirheumatic drugs; MTX: methotrexate; HXQ:, hydroxychloroquine; LFN, leflunomide; SSZ: sulfasalazine; OR, odds ratio; CI, confidence interval; NA: not available (indicates non-estimable values due to sparse data or quasi-complete separation); *: p value for Fisher's Exact Test

Table S43. Filgotinib EULAR response bivariate genetic analyses

SNPs	3 months							6 months					
	Genotipe	N	Satisfactory	Unsatisfactory	p-value	OR	$CI_{95\%}$	N	Satisfactory	Unsatisfactory	p-value	OR	$CI_{95\%}$
JAK1													
**rs2230587**	AG	5	1 (20)	4 (80)				5	2 (40)	3 (60)			
	GG	15	6 (40)	9 (60)	0.612	-	-	12	8 (66.7)	4 (44.4)	0.592	-	-
	AA	0	0	0				0	0	0			
	A	5	1 (20)	4 (80)	0.612	-	-	5	2 (40)	3 (60)	0.592	-	-
	G	-	-	-	-	-	-	-	-	-	-	-	-
**rs310241**	AA	13	3 (30.8)	9 (69.2)				11	6 (54.5)	5 (45.5)			
	AG	6	2 (33.3)	4 (66.7)	0.566	-	-	5	3 (60)	2 (40)	1	-	-
	GG	1	1 (100)	0 (0)				1	1 (100)	0 (0)			
	A	19	6 (31.6)	13 (68.4)	0.350	-	-	16	9 (56.2)	7 (43.8)	1	-	-
	G	7	3 (42.9)	4 (57.1)	0.651	-	-	6	4 (66.7)	2 (33.3)	1	-	-
**rs2230588**	CC	1	1 (100)	0 (0)				1	1 (100)	0 (0)			
	CT	6	1 (16.7)	5 (83.3)	0.295	-	-	5	3 (60)	2 (40)	1	-	-
	TT	13	5 (38.5)	8 (61.5)				11	6 (54.4)	5 (45.5)			
	T	19	6 (31.6)	13 (68.4)	0.350	-	-	16	9 (56.2)	7 (43.8)	1	-	-
	C	6	1 (16.7)	5 (83.3)	0.295	-	-	6	4 (66.7)	2 (33.3)	1	-	-
**rs10889504**	CG	3	0 (0)	3 (100)				3	1 (33.3)	2 (66.7)			
	GG	17	7 (41.2)	10 (58.8)	0.521	-	-	14	9 (64.3)	5 (35.7)	0.536	-	-
	CC	0	0	0				0	0	0			
	C	3	0 (0)	3 (100)	0.521	-	-	3	1 (33.3)	2 (66.7)	0.536	-	-
	G	-	-	-	-	-	-	-	-	-	-	-	-
**rs2780815**	GG	4	2 (50)	2 (50)				4	2 (50)	2 (50)			
	GT	8	1 (12.5)	7 (87.5)	0.292	-	-	7	4 (57.1)	3 (42.9)	1	-	-
	TT	8	4 (50)	4 (50)				6	4 (66.7)	2 (33.3)			
	T	16	5 (31.2)	11 (68.8)	0.586	-	-	13	8 (61.5)	5 (38.5)	1	-	-
	G	12	3 (25)	9 (75)	0.356			11	6 (54.5)	5 (45.5)	1	-	-
JAK2													
**rs10119004**	AA	5	2 (40)	3 (60)				5	2 (40)	3 (60)			
	AG	9	3 (33.3)	6 (66.7)	1	-	-	7	4 (57.1)	3 (42.9)	0.604	-	-
	GG	6	2 (33.3)	4 (66.7)				5	4 (80)	1 (20)			
	A	14	5 (35.7)	9 (64.3)	1	-	-	12	6 (50)	6 (50)	0.338	-	-
	G	15	5 (33.3)	10 (66.7)	1	-	-	12	8 (66.7)	4 (33.3)	0.592	-	-

	GG	5	2 (40)	3 (60)				5	3 (60)	2 (40)			
	GT	8	2 (25)	6 (75)	0.848	-	-	6	4 (66.7)	2 (33.3)	1	-	-
**rs7857730**	TT	7	3 (42.9)	4 (57.1)				6	3 (50)	3 (50)			
	G	13	4 (30.8)	9 (69.2)	0.651	-	-	11	7 (63.6)	4 (36.4)	0.643	-	-
	T	15	5 (33.3)	10 (66.7)	1	-	-	12	7 (58.3)	5 (41.7)	1	-	-
	CC	3	2 (66.7)	1 (33.3)				3	2 (66.7)	1 (33.3)			
	CT	12	3 (25)	9 (75)	0.457	-	-	9	6 (66.7)	3 (33.3)	0.805	-	-
**rs2274472**	TT	5	2 (40)	3 (60)				5	2 (40)	3 (60)			
	C	15	5 (33.3)	10 (66.7)	1	-	-	12	8 (66.7)	4 (33.3)	0.592	-	-
	T	17	5 (29.4)	12 (70.6)	0.270	-	-	14	8 (57.1)	6 (42.9)	1	-	-
	CC	11	4 (36.4)	7 (63.6)				9	6 (66.7)	3 (33.3)			
	CT	6	2 (33.3)	4 (66.7)	1	-	-	6	2 (33.3)	4 (66.7)	0.328	-	-
**rs2230722**	TT	3	1 (33.3)	2 (66.7)				2	2 (100)	0 (0)			
	C	17	6 (35.3)	11 (64.7)	1	-	-	15	8 (53.3)	7 (46.7)	0.485	-	-
	T	9	3 (33.3)	6 (66.7)	1	-	-	8	4 (50)	4 (50)	0.637	-	-
	AA	7	3 (42.9)	4 (57.1)				6	3 (50)	3 (50)			
	AG	7	1 (14.3)	6 (85.7)	0.507	-	-	5	3 (60)	2 (40)	1	-	-
**rs2230724**	GG	6	3 (50)	3 (50)				6	4 (66.7)	2 (33.3)			
	A	14	4 (28.6)	10 (71.4)	0.612	-	-	11	6 (54.5)	4 (45.5)	1	-	-
	G	13	4 (30.8)	9 (69.2)	0.651	-	-	11	7 (63.6)	4 (36.4)	0.643	-	-
JAK3													
	AA	2	0 (0)	2 (100)				2	1 (50)	1 (50)			
	AG	9	3 (33.3)	6 (66.7)	0.818	-	-	7	4 (57.1)	3 (42.9)	1	-	-
**rs3212780**	GG	9	4 (44.4)	5 (55.6)				8	5 (62.5)	3 (37.5)			
	A	11	3 (27.3)	8 (72.7)	0.642	-	-	9	5 (55.6)	4 (44.4)	1	-	-
	G	18	7 (38.9)	11 (61.1)	0.521	-	-	15	9 (60)	6 (40)	1	-	-
	AA	2	0 (0)	2 (100)		1	-	1	1 (100)	0 (0)			
	AG	12	7 (58.3)	5 (41.7)	0.029*	2.27×10^{-9}	[NA - NA]	11	7 (63.6)	4 (36.4)	0.762	-	-
**rs3008**	GG	6	0 (0)	6 (100)		1	[NA]	5	2 (40)	3 (60)			
	A	14	7 (50)	7 (50)	0.051*	1.16×10^{8}	[1.37×10^{-101} – NA]	12	8 (66.7)	4 (33.3)	0.592	-	-
	G	18	7 (38.9)	11 (61.1)	0.5211	-	-	16	9 (56.2)	7 (43.8)	1	-	-
	CT	1	0 (0)	1 (100)					-	-			
	TT	19	7 (36.8)	12 (63.2)	1	-	-	-	-	-	-	-	-
**rs3212752**	CC	0	0	0					-	-			
	C	1	0 (0)	1 (100)	1	-	-	-	-	-	-	-	-
	T	-	-	-	-	-	-	-	-	-	-	-	-

EULAR: European League Against Rheumatism; OR: odds ratio; CI: confidence interval; NA: not available (indicates non-estimable values due to sparse data or quasi-complete separation); *: p value for Fisher's Exact Test

Table S44. Filgotinib LDA bivariate demographic and clinical analyses

Clinical variables	3 months						6 months					
	N	LDA		OR	$CI_{5\%}$	p-value	N	LDA		OR	$CI_{95\%}$	p-value
		LDA	No LDA					LDA	No LDA			
**Sex**												
Woman	18	3 (16.7)	15 (83.3)	-	-	0.368*	15	3 (20)	12 (80)	-	-	0.426*
Man	2	1(50)	1 (50)				2	1 (50)	1 (50)			
**Smoking**												
Smoker	2	0 (0)	2 (100)				1	0 (0)	1 (100)			
Exsmoker	2	0 (0)	2 (100)	-	-	1*	2	0 (0)	2 (100)	-	-	1*
No smoker	16	4 (25)	12 (75)				14	4 (28.6)	10 (71.4)			
**Age at Dx**	20	33.2 ± 8	41 ± 11.1	-	-	0.160	17	40.7 ± 14.2	38.3 ± 9.3	-	-	0.764
**Years with RA**	20	11.5 ± 8.3	13.6 ± 8	-	-	0.668	17	13.7 ± 9.5	13 ± 8.3	-	-	0.893
**Years from Dx till JAK inhibitor treatment**	20	6.5 [4.2-10.7]	9.5 [3.7-17]	-	-	0.554	17	11.2 ± 9.5	10.6 ± 8.4	-	-	0.91
**JAK inhibitors start age**	20	44.5 [37-49.5]	54.5 [46.7-57.7]	-	-	0.138	17	52.2 ± 5.5	49.1 ± 10	-	-	0.436
**Treatment duration with JAK inhibitor (months)**	20	22.5 [8.2-36.4]	11.6 [6.8-26.9]	-	-	0.548	17	13.7 [7.1-24.3]	19.3 [8.7-32.7]	-	-	0.712
**Number of previous BTs**	20	1.7 ± 0.9	2.5 ± 1.8	-	-	0.249	17	2.7 ± 1.5	2.1 ± 1.6	-	-	0.523
**Previous BTs duration (months)**	20	26.5 [2.7-72.5]	46.5 [15.7-6]	-	-	0.789	17	81 [19.5-159.5]	18 [5-55]	-	-	0.341
**BTs cause of suspensión**												
**Primary failure**	7	1 (14.3)	6 (85.7)	-	-	1*	5	1 (20)	4 (80)	-	-	1*
**Secondary failure**	10	3 (30)	7 (70)	-	-	0.582*	9	3 (33.3)	6 (66.7)	-	-	0.576*
**Adverse events**	3	0 (0)	3 (100)	-	-	1*	3	0 (0)	3 (100)			0.541*
**Toxicity**	0	-	-	-	-	-	0	-	-	-	-	-
**Others**	0	-	-	-	-	-	0	-	-	-	-	-
**Baseline RF**												
**Pos**	14	2 (14.3)	12 (85.7)	-	-	0.549*	8	1 (12.5)	7 (87.5)	-	-	0.576*
**Neg**	6	2 (33.3)	4 (66.7)				9	3 (33.3)	6 (66.7)	-	-	
**Baseline ACPA**												
**Pos**	18	4 (22.2)	14 (77.8)	-	-	1*	15	3 (20)	12 (80)	-	-	0.426*
**Neg**	2	0 (0)	2 (100)				2	1 (50)	1 (50)			
**Baseline CCI**												
**Absence**	13	3 (23.1)	10 (76.9)				12	3 (25)	9 (75)			
**Low**	6	1 (16.7)	5 (83.3)	-	-	1*	4	1 (25)	3 (75)	-	-	1*
**High**	1	0 (0)	1 (100)				1	0 (0)	1 (100)			
**BMI**	20	29.8 ± 5.1	29.3 ± 5.4	-	-	0.852	17	29.9 ± 4.8	28.9 ± 4.4	-	-	0.718
**JAK inhibitors dose change**												
yes	2	0 (0)	2 (100)	-	-	1*	2	0 (0)	2 (100)	-	-	1*
No	18	4 (22.2)	14 (77.8)				15	4 (26.7)	11 (73.3)			

**JAK inhibitor suspensión**												
yes	7	1 (14.3)	6 (85.7)	-	-	1*	4	1 (25)	3 (75)	-	-	1*
No	13	3 (23.1)	10 (76.9)				13	3 (23.1)	10 (76.9)			
**JAK inhibitor cause of suspension**												
Primary failure	2	0 (0)	2 (100)	-	-	0.571*	1	0 (0)	1 (100)	-	-	1*
Secondary failure	2	1 (50)	1 (50)				1	0 (0)	1 (100)			
Adverse events	3	0 (0)	3 (100)				2	1 (50)	1 (50)			
Others	0	-	-				0	-	-			
**BT after JAK inhibitor treatment**												
Yes	7	0 (0)	7 (100)	-	-	0.248*	4	0 (0)	4 (100)	-	-	0.519*
No	13	4 (30.8)	9 (69.2)				13	4 (30.8)	9 (69.2)			
**Adverse events to JAK inhibitors**												
Yes	7	2 (28.6)	5 (71.4)	-	-	0.586*	6	3 (50)	3 (50)	-	-	0.098*
No	13	2 (15.4)	11 (84.6)				11	1 (9.1)	10 (90.9)			
**Concomitant DMARDs**												
MTX	7	0 (0)	7 (100)	-	-	0.396*	5	1 (20)	4 (80)	-	-	0.283*
HXQ	1	0 (0)	1 (!00)				1	1 (100)	0 (0)			
SSZ	0	-	-				0	-	-			
LFN	0	-	-				0	-	-			
None	12	4 (33.3)	8 (66.7)				11	2 (18.2)	9 (81.8)			
**Concomitant statins**												
yes	5	1 (20)	4 (80)	-	-	1*	5	2 (40)	3 (60)	-	-	0.537*
No	15	3 (20)	12(80)				12	2 (16.7)	10 (83.3)			
**Concomitant GC**												
yes	10	2 (20)	8 (80)	-	-	1*	8	2 (25)	6 (75)	-	-	1*
No	10	2 (20)	8 (80)				9	2 (22.2)	7 (77.8)			
**Concomitant vitamin D**												
Si	7	0 (0)	7 (100)	-	-	0.248*	6	0 (0)	6 (100)	-	-	0.237
No	13	4 (30.8)	9 (69.2)				11	4 (36.4)	7 (63.6)			
**Baseline DAS28**	20	2.4 ± 0.8	3.8 ± 1.3	1.57×10^9	[0-NA]	0.043	17	2.7±0.3	3.1 ± 1.5	-	-	0.436*
**Baseline TJC**	20	1 [0-2.2]	4 [1.7-6.5]	3.70×10^{18}	[0-NA]	0.009	17	2 [1.5-2]	5 [1-7]	1.44	[0.96-3.09]	0.025
**Baseline SJC**	20	0 [0-0]	2 [0-4]	2.40×10^8	[0-NA]	0.002	17	0 [0-0.25]	1 [0-3]	2.53	[0.85-40.42]	0.043
**Baseline PVAS**	20	3.2 ± 2	5.6 ± 2.2	3.90×10^6	[0-NA]	0.099	17	3.5 [3-4.7]	5 [4-6]	-	-	0.809
**Baseline MVAS**	20	3.2 ± 2.1	4.9 ± 2.2	6.22×10^6	[0-NA]	0.209	17	3 [2.7-4]	3 [2-5]	-	-	0.900
**Baseline CRP**	20	1.9 [0.8-3.6]	1.9 [0.5-4.5]	-	-	0.169	17	3.7 [2.1-7.2]	2.2 [0.6-3.1]	-	-	0.504
**Baseline ESR**	20	11.5 [6-23.7]	15.5 [8.7-21.2]	-	-	0.864	17	11.5 [8.7-14.7]	10 [6-18]	-	-	0.365
**Baseline RF (Quantitative)**	20	17.5 [13.2-298.7]	39 [17.2-76.2]	-	-	0.530	17	11.5 [10-23]	20 [10-52]	-	-	0.155*
**Baseline TC**	20	171 ± 23.4	220 ± 31.7	17.4	[0-NA]	0.012	17	209.5 ± 29.1	206.6 ± 38.4	-	-	0.881
**Baseline LDL**	20	105.7 ± 32.5	120.5 ± 21.5	-	-	0.440	17	131.7 ± 17.9	115.9 ± 29.1	-	-	0.224
**Baseline TG**	20	88 [57-133.5]	80.5 [62-100.5]	-	-	0.755	17	125.2 ± 23.1	69 ±21.9	0.79	[0.34-0.95]	0.008

LDA: Low disease activity; TJC: tender joints count; SJC: swallowed joint count; PVAS: patient visual analogue scale; MVAS: physician visual analogue scale; RF: rheumatoid factor; ACPA: anti-citrullinated protein antibodies; ESR: erythrocyte sedimentation rate; CRP: C-reactive protein; TC: total cholesterol; LDL: low-density lipoprotein; TG: triglycerides; BMI: body mass index; CCI: Charlson Comorbidity Index; JAK inhibitor: Janus kinase inhibitor; BTs: biologic therapies; GC: glucocorticoids; DMARDs: disease-modifying antirheumatic drugs; MTX: methotrexate; HXQ:, hydroxychloroquine; LFN, leflunomide; SSZ: sulfasalazine; OR, odds ratio; CI, confidence interval; NA: not available (indicates non-estimable values due to sparse data or quasi-complete separation); *: p value for Fisher's Exact Test.

Table S45. Filgotinib LDA bivariate genetic analyses

SNPs	Genotipe	3 months					6 months				
		N	LDA		OR	p-value	N	LDA		OR	p-value
			LDA	No LDA	CI95%			LDA	No LDA	CI95%	
JAK1											
**rs2230587**	**GG**	15	3 (20)	12 (80)	-	1*	12	3 (25)	9 (75)	-	1*
	**AA**	0	-	-			0	-	-		
	**AG**	5	1 (20)	4 (80)			5	1 (20)	4 (80)		
	**A**	5	1 (20)	4 (80)	-	1*	5	1 (20)	4 (80)	-	1*
	**G**	-	-	-	-	-	-	-	-	-	-
**rs310241**	**GG**	1	0 (0)	1 (100)	-	0.645*	1	0 (0)	1 (100)	-	1*
	**AA**	13	2 (15.4)	11 (84.6)			11	3 (27.3)	8 (72.7)		
	**AG**	6	2 (33.3)	4 (66.7)			5	1 (20)	4 (80)		
	**A**	19	4 (21.1)	15 (78.9)	-	1*	16	4 (25)	12 (75)	-	1*
	**G**	7	2 (28.6)	5 (71.4)	-	0.586*	6	1 (16.7)	5 (83.3)		1*
**rs2230588**	**CC**	1	0 (0)	1(100)	-	1*	1	0 (0)	1 (100)	-	0.653*
	**TT**	13	3 (23.1)	10 (76.9)			11	2 (18.2)	9 (81.8)		
	**CT**	6	1 (16.7)	5 (83.3)			5	2 (40)	3 (60)		
	**T**	19	4 (21.1)	15 (78.9)	-	1*	16	4 (25)	12 (75)	-	1*
	**C**	7	1 (14.3)	6 (85.7)	-	1*	6	2 (33.3)	4 (66.7)	-	0.584*
**rs10889504**	**GG**	17	4 (23.5)	13 (76.5)	-	1*	14	3 (21.4)	11 (78.6)	-	1*
	**CC**	0	-	-			0	-	-		
	**CG**	3	0 (0)	3 (100)			3	1 (33.3)	2 (66.7)		
	**C**	3	0 (0)	3 (100)	-	1*	3	1 (33.3)	2 (66.7)	-	1*
	**G**	-	-	-	-	-	-	-	-	-	-
**rs2780815**	**GG**	4	1 (25)	3 (75)	-	1*	4	0 (0)	4 (100)	-	0.611*
	**TT**	8	2 (25)	6 (75)			6	2 (33.3)	4 (66.7)		
	**GT**	8	1 (12.5)	7 (87.5)			7	2 (28.6)	5 (71.4)		
	**T**	16	3 (18.8)	13 (81.2)	-	1*	13	4 (30.8)	9 (69.2)	-	0.519*
	**G**	12	2 (16.7)	10 (83.3)	-	1*	11	2 (18.2)	9 (81.8)	-	0.584*
JAK2											
**rs10119004**	**GG**	6	1 (16.7)	5 (83.3)	-	1*	5	2 (20)	3 (80)	-	0.779*
	**AA**	5	1 (20)	4 (80)			5	1 (20)	4 (80)		
	**AG**	9	2 (22.2)	7 (77.8)			7	1 (14.3)	6 (85.7)		
	**A**	14	3 (21.4)	11 (78.6)	-	1*	12	2 (16.7)	10 (83.3)	-	0.537*
	**G**	15	3 (20)	12 (80)	-	1*	12	3 (25)	9 (75)	-	1*
**rs7857730**	**GG**	5	0 (0)	5 (100)	-	0.624	5	1 (20)	4 (80)	-	1*
	**TT**	7	2 (25)	6 (75)			6	2 (33.3)	4 (66.7)		
	**GT**	8	2 (28.6)	5 (71.4)			6	1 (16.7)	5 (83.3)		
	**G**	13	2 (15.4)	11 (84.6)	-	0.586	11	3 (27.3)	8 (72.7)	-	1*
	**T**	15	4 (26.7)	11 (73.3)	-	0.530	12	3 (25)	9 (75)	-	1*
**rs2274472**	**CC**	3	1 (33.3)	2 (66.7)	-	0.773*	3	1 (33.3)	2 (66.7)	-	0.331*
	**TT**	5	1 (20)	4 (80)			5	0 (0)	5 (100)		
	**CT**	12	2 (16.7)	12 (83.3)			9	3 (33.3)	6 (66.7)		
	**C**	15	3 (20)	12 (80)	-	1*	12	4 (33.3)	8 (66.7)	-	0.260*
	**T**	17	3 (17.6)	14 (82.4)	-	0.508*	14	3 (21.4)	11 (78.6)	-	1*
**rs2230722**	**CC**	11	2 (18.2)	9 (81.8)	-	0.591*	9	3 (33.3)	6 (66.7)	-	0.773*
	**TT**	3	0 (0)	3 (100)			2	0 (0)	2 (100)		
	**CT**	6	2 (33.3)	4 (66.7)			6	1 (16.7)	5 (83.3)		
	**C**	17	4 (23.5)	13 (76.5)	-	1*	15	4 (26.7)	11 (73.3)	-	1*
	**T**	9	2 (2.2)	7 (77.8)	-	1*	8	1 (12.5)	7 (87.5)	-	0.576
**rs2230724**	**GG**	6	1 (16.7)	5 (83.3)	-	1*	6	2 (33.3)	4 (66.7)	-	1*
	**AA**	7	2 (28.6)	5 (71.4)			6	1 (16.7)	5 (83.3)		
	**AG**	7	1 (14.3)	6 (85.7)			5	1 (20)	4 (80)		
	**A**	14	3 (21.4)	11 (78.6)	-	1*	11	2 (!8.2)	9 (81.8)	-	0.584*
	**G**	13	2 (15.4)	11 (84.6)	-	0.586*	11	3 (27.3)	8 (72.7)	-	1*
JAK3											
**rs3212780**	**GG**	9	3 (33.3)	6 (66.7)	-	0.732*	8	1 (12.5)	7 (87.5)	-	0.423*
	**AA**	2	0 (0)	2 (100)			2	1 (50)	1 (50)		
	**AG**	9	1 (11.1)	8 (88.9)			7	2 (28.6)	5 (71.4)		
	**A**	11	1 (9.1)	10 (90.9)	-	0.284*	9	3 (33.3)	6 (66.7)	-	0.576*
	**G**	18	4 (22.2)	14 (77.8)	-	1*	15	3 (20)	12 (80)	-	0.426*
**rs3008**	**GG**	6	0 (0)	6 (100)	-	0.359*	5	2 (40)	3 (60)	6.38×10^7 [0–NA]	0.098*

	**AA**	2	0 (0)	2 (2)			1	1 (100)	0 (0)	1	
	**AG**	12	4 (33.3)	8 (66.7)			11	1 (9.1)	10 (90.9)	4.25 × 10⁸ [0–NA]	
	**A**	14	4 (28.6)	10 (71.4)	-	0.267*	12	2 (16.7)	10 (83.3)	-	0.537*
	**G**	18	4 (22.2)	14 (77.8)	-	1*	16	3 (18.8)	13 (81.2)	-	0.235*
**rs3212752**	**TT**	19	4 (21.1)	15 (78.9)	-	1*	-	-	-	-	-
	**CC**	1	0 (0)	1 (100)			-	-	-		
	**CT**	-	-	-			-	-	-		
	**C**	1	0	1 (100)	-	1*	-	-	-	-	-
	**T**	-	-	-	-	-	-	-	-	-	-

LDA:Low disease activity; OR: odds ratio; CI: confidence interval; NA: not available (indicates non-estimable values due to sparse data or quasi-complete separation); *: p value for Fisher's Exact Test

Table S46. Filgotinib remission bivariate demographic and clinical analyses

**Clinical variables**	**3 meses**						**6 meses**					
	**N**	**Remission**		**OR**	**CI95%**	**p-value**	**N**	**Remission**		**OR**	**CI95%**	**p-value**
		**Remission**	**No Remission**					**Remission**	**No Remission**			
**Sex**												
Woman	18	4 (22.2)	14 (77.8)	-	-	1*	15	6 (40)	9 (60)	-	-	0.514*
Man	2	0 (0)	2 (100)				2	0 (0)	2 (100)			
**Smoking**												
Smoker	2	0 (0)	2 (100)	-	-	0.624*	1	1 (100)	0 (0)	-	-	0.433*
Exsmoker	2	1 (50)	1 (50)				2	1 (50)	1 (50)			
No smoker	16	3 (18.8)	13 (81.2)				14	4 (28.6)	10 (71.4)			
**Age at Dx**	20	37 ± 8.8	40.1±11.4	-	-	0.587	17	39.5±8.9	38.5±11.3	-	-	0.851
**Years with RA**	20	17.2 ± 12.1	12.1±6.6	-	-	0.473	17	13.5 ± 11.2	13±6.9	-	-	0.923
**Years from Dx till JAK inhibitor treatment**	20	14 ±12.5	9.8±6.7	-	-	0.558	17	10.6 ± 11.1	10.8 ± 7.2	-	-	0.976
**JAK inhibitors start age**	20	51 ±3.6	50.2 ±10.8	-	-	0.806	17	50.1 ± 6.2	49.6±10.6	-	-	0.898
**Treatment duration with JAK inhibitor (months)**	20	32.6 [28.3-36.3]	8.4 [6.7-19.5]	0.86	[0.60-0.99]	<0.001	17	32.5 [26.9-36.2]	10.6 [7.1-19.8]	0.89	[0.78-0.98]	0.031
**Number of previous BTs**	20	1.2 ± 1.2	2.6 ±1.7	-	-	0.106	17	1.5±1.2	2.7±1.6	-	-	0.102
**Previous BTs duration (months)**	20	11.5 [4.5-26]	51.5 [15.7-91.2]	1.03	[0.99-1.10]	0.028	17	11.5 [3.5-42]	51 [14.5-112]	1.02	[0.99-1.07]	0.063
**BTs cause of suspensión**												
**Primary failure**	7	2 (28.6)	5 (71.4)	-	-	0.586*	5	3 (60)	2 (40)	-	-	0.280*
**Secondary failure**	10	1 (10)	9 (90)	-	-	0.582*	9	2 (22.2)	7 (77.8)	-	-	0.334*
**Adverse events**	3	1 (33.3)	2 (66.7)	-	-	0.508*	3	1 (33.3)	2 (66.7)	-	-	1*
**Toxicity**	0	-	-	-	-	-	0	-	-	-	-	-
**Others**	0	-	-	-	-	-	0	-	-	-	-	-
**Baseline RF (Cualitative)**												
**Pos**	14	1 (7.1)	13 (92.9)	13	[1.20-323.74]	0.060*	8	2 (25)	6 (75)	-	-	0.619*
**Neg**	6	3 (50)	3 (50)				9	4 (44.4)	5 (55.6)			
**Baseline ACPA**												
**Pos**	18	3 (16.7)	15 (83.3)	-	-	0.368*	15	5 (33.3)	10 (66.7)	-	-	1*
**Neg**	2	1 (50)	1 (50)				2	1 (50)	1 (50)			
**Baseline CCI**												
**Absence**	13	3 (23.1)	10 (76.9)	-	-	1*	12	4 (33.3)	8 (66.7)	-	-	0.504*
**Low**	6	1 (16.7)	5 (76.9)				4	1 (25)	3 (75)			
**High**	1	0 (0)	1 (100)				1	1 (100)	0 (0)			
**BMI**	20	26.9±3.9	30±5.4	-	-	0.242	17	27.4±3.5	30.1±4.66	-	-	0.217
**JAK inhibitor dose change**												
**yes**	2	2 (100)	0 (0)	1 × 10⁻⁹	[NA-1.11 × 10³⁰⁴]	0.031*	2	2 (100)	0 (0)	-	-	0.110*
**No**	18	2 (11.1)	16 (88.9)				15	4 (26.7)	11 (73.3)			
**JAK inhibitor suspension**												
**yes**	7	0 (0)	7 (100)	-	-	0.248*	4	0 (0)	4 (100)	-	-	0.237*
**No**	13	4 (30.8)	9 (69.2)				13	6 (46.2)	7 (53.8)			
**JAK inhibitor cause of suspension**												
**Primary failure**	7	2 (28.6)	5 (71.4)	-	-	0.586*	1	0 (0)	1 (100)	-	-	1*
**Secondary failure**	10	1(10)	9 (90)	-	-	0.582*	1	0 (0)	1 (100)			
**Adverse events**	3	1 (33.3)	2 (66.7)	-	-	0.508*	2	0 (0)	2 (100)			
**Others**	0	-	-	-	-	-	0	-	-			
**BTs after JAK inhibitor treatment**												
**yes**	7	0 (0)	7 (100)	-	-	0.248	4	0 (0)	4 (100)	-	-	0.237*
**No**	13	4 (30.8)	9 (69.2)				13	6 (46.2)	7 (53.8)			
**Adverse events to JAK inhibitors**												
**yes**	7	1 (14.3)	6 (85.7)	-	-	1*	6	2 (33.3)	4 (66.7)	-	-	1*
**No**	13	3 (23.1)	10 (76.9)				11	4 (36.4)	7 (63.6)			
**Concomitant DMARDs**												
**MTX**	7	1 (14.3)	6 (85.7)	-	-	1*	5	1 (20)	4 (80)	-	-	0.733*
**HXQ**	1	0 (0)	1 (100)				1	0 (0)	1 (100)	-	-	
**SSZ**	0	-	-				0	-	-			
**LFN**	0	-	-				0	-	-			
**None**	12	3 (25)	9 (75)				11	5 (45.5)	6 (54.5)			
**Concomitant statins**												
**yes**	5	0 (0)	5 (100)	-	-	0.530*	5	1 (20)	4 (80)	-	-	0.6*
**No**	15	4 (26.5)	11 (73.3)				12	5 (41.7)	7 (58.3)			
**Concomitant GC**												
**yes**	10	0 (0)	10 (100)	2.10 × 10⁸	[1.98 × 10⁻¹⁶⁵-NA]	0.086*	8	2 (25)	6 (75)	-	-	0.619*
**No**	10	4 (40)	6 (60)				9	4 (44.4)	5 (55.6)			
**Concomitant vitamin D**												
**yes**	7	1 (14.3)	6 (85.7)	-	-	1*	6	2 (33.3)	4 (66.7)	-	-	1*
**No**	13	3 (23.1)	10 (76.9)				11	4 (36.4)	7 (63.6)			
**Baseline DAS28**	20	1.4 ±0.5	4.1±0.9	4.33×10⁴⁵	[0-NA]	<0.001	17	1.6 ±0.5	3.7±1.1	5.41 × 10⁸⁴	[0-NA]	<0.001
**Baseline TJC**	20	0 [0-0.2]	4 [2.7-6.5]	7.51	[1.65-242.30]	<0.001	17	0.5 [0-1.7]	6 [2-7.5]	1.86	[1.13-4.72]	0.009
**Baseline SJC**	20	0 [0-0]	2 [0-4]	4.44×10⁷	[NA-NA]	0.002	17	0 [0-0]	1 [0-3]	4.19	[1.17-81.11]	0.017
**Baseline PVAS**	20	2.7±2.1	5.7±2	2.20	[1.16-6.13]	0.051	20	2.8 ±1.9	5.3±1.8	2.39	[1.18-8.77]	0.025
**Baseline MVAS**	20	2.5±1.9	5.1±2	2.04	[1.09-5.36]	0.061	20	1.8±1.16	5±2.1	6.04	[1.55-131.35]	0.001
**Baseline CRP**	20	1.6 [1.2-2.1]	1.9 [0.5-6.3]	-	-	0.136	17	0.8 [0.6-1.9]	3.1 [1.8-7]	2.32	[1.11-9.46]	0.023
**Baseline ESR**	20	13 [7.7-21.3]	17.5 [8.7-42.2]	-	-	0.442	17	7.5 [4.2-16]	11 [8-17]	-	-	0.491
**Baseline RF (Quantitative)**	20	10.5 [4.8-16.2]	59.5 [19.7-120.5]	1.12	[1.01-1.42]	0.059	17	11.5 [8.5-18.2]	20 [10-52.5]	-	-	0.860
**Baseline TC**	20	219.2 ± 42.9	207.9 ± 35.1	-	-	0.650	17	218.6 ± 37.4	201.2 ± 34.7	-	-	0.368
**Baseline LDL**	20	127.2 ±24.7	115.1 ± 23.8	-	-	0.425	17	130.3 ± 29.5	113.8±25.5	-	-	0.277
**Baseline TG**	20	61.5 [55.7-75.7]	87.5 [63-113.5]	-	-	0.149	17	58 ± 12.5	95.4± 32.8	1.07	[1.01-1.18]	0.004*

TJC: tender joints count; SJC: swallowed joint count; PVAS: patient visual analogue scale; MVAS: physician visual analogue scale; RF: rheumatoid factor; ACPA: anti-citrullinated protein antibodies; ESR: erythrocyte sedimentation rate; CRP: C-reactive protein; TC: total cholesterol; LDL: low-density lipoprotein; TG: triglycerides; BMI: body mass index; CCI: Charlson Comorbidity Index; JAK inhibitor: Janus kinase inhibitor; BTs: biologic therapies; GC: glucocorticoids; DMARDs: disease-modifying antirheumatic drugs; MTX: methotrexate; HXQ:, hydroxychloroquine; LFN, leflunomide; SSZ: sulfasalazine; OR, odds ratio; CI, confidence interval; NA: not available (indicates non-estimable values due to sparse data or quasi-complete separation); *: p value for Fisher's Exact Test

Table S47. Filgotinib remission bivariate genetic analyses

**SNPs**	**Genotipe**	**3 meses**					**6 meses**				
		**N**	**Remission**		**OR CI95%**	**p-value**	**N**	**Remission**		**OR CI95%**	**p-value**
			**Remission**	**No Remission**				**Remission**	**No Remission**		
JAK1											
**rs2230587**	**GG**	15	4 (26.7)	11 (73.3)	-	0.530*	12	5 (41.7)	7 (58.3)	-	0.600*
	**AA**	0	-	-			0	-	-		
	**AG**	5	0 (0)	5 (100)			5	1 (20)	4 (80)		
	**A**	5	0 (0)	5 (100)	-	0.530*	5	1 (20)	4 (80)	-	0.600*
	**G**	-	-	-	-	-	-	-	-	-	-

rs310241	**GG**	1	1 (100)	0 (0)			1	1 (100)	0 (0)		
	**AA**	13	2 (15.4)	11 (84.6)	-	0.256*	11	3 (27.3)	8 (72.7)	-	0.546*
	**AG**	6	1 (16.7)	5 (83.3)			5	2 (40)	3 (60)		
	**A**	19	3 (15.8)	16 (84.2)	-	0.200*	16	5 (31.2)	11 (68.8)	-	0.352*
	**G**	7	2 (28.6)	5 (71.4)	-	0.586*	6	3 (50)	3 (50)	-	0.600*
rs2230588	**CC**	1	1 (100)	0 (0)			1	1 (100)	0 (0)		
	**TT**	13	2 (15.4)	11 (84.6)	-	0.256*	11	4 (36.4)	7 (63.6)	-	0.546*
	**CT**	6	1 (16.7)	5 (83.3)			5	1 (20)	4 (80)		
	**T**	19	3 (15.8)	16 (84.2)	-	0.200*	16	5 (31.2)	11 (68.8)	-	0.352*
	**C**	7	2 (28.6)	5 (71.4)	-	0.586*	6	2 (33.3)	4 (66.7)	-	1*
rs10889504	**GG**	17	4 (23.5)	13 (76.5)			14	6 (42.9)	8 (57.1)		
	**CC**	0	-	-	-	1*	0	-	-	-	0.514*
	**GC**	3	0 (0)	3 (100)			3	0 (0)	3 (100)		
	**C**	3	0 (0)	3 (100)	-	1*	3	0 (0)	3 (100)	-	0.514*
	**G**	-	-	-	-	-	-	-	-	-	-
rs2780815	**GG**	4	1 (25)	3 (75)			4	2 (50)	2 (50)		
	**TT**	8	2 (25)	5 (75)	-	1*	6	2 (33.3)	4 (66.7)	-	0.830*
	**GT**	8	1 (12.5)	7 (87.5)			7	2 (28.6)	5 (71.4)		
	**T**	16	3 (18.8)	13 (81.2)	-	1*	13	4 (30.8)	9 (69.2)	-	0.584*
	**G**	12	2 (16.7)	10 (83.3)	-	1*	11	4 (36.4)	7 (63.6)	-	1*
JAK2											
rs10119004	**GG**	6	1 (16.7)	5 (83.3)			5	2 (40)	3 (60)		
	**AA**	5	1 (20)	4 (80)	-	1*	5	1 (20)	4 (80)	-	0.830*
	**AG**	9	2 (22.2)	7 (77.8)			7	3 (42.9)	4 (57.1)		
	**A**	14	3 (21.4)	11 (78.6)	-	1*	12	4 (33.3)	8 (66.7)	-	1*
	**G**	15	3 (20)	12 (80)	-	1*	12	5 (41.7)	7 (58.3)	-	0.600*
rs7857730	**GG**	5	2 (40)	3 (60)			5	2 (40)	3 (60)		
	**TT**	7	2 (28.6)	5 (71.4)	-	0.133*	6	2 (33.3)	4 (66.7)	-	1*
	**GT**	8	0 (0)	8 (100)			6	2 (33.3)	4 (66.7)		
	**G**	13	2 (15.4)	11 (84.6)	-	0.586*	11	4 (36.4)	7 (63.6)	-	1*
	**T**	15	3 (13.3)	12 (86.7)	-	0.248*	12	4 (33.3)	8 (66.7)	-	1*
rs2274472	**CC**	3	1 (33.3)	2 (66.7)			3	1 (33.3)	2 (66.7)		
	**TT**	5	2 (40)	3 (60)	-	0.194*	5	2 (40)	3 (60)	-	1*
	**CT**	12	1 (8.3)	11 (91.7)			9	3 (33.3)	6 (66.7)		
	**C**	15	2 (13.3)	13 (86.7)	-	0.248*	12	4 (33.3)	8 (66.7)	-	1*
	**T**	17	3 (17.6)	14 (82.4)	-	0.508*	14	5 (35.7)	9 (64.3)	-	1*
rs2230722	**CC**	11	3 (27.3)	8 (72.7)			9	3 (33.3)	6 (66.7)		
	**TT**	3	1 (33.3)	2 (66.7)	-	0.421*	2	2 (100)	0 (0)	-	0.121*
	**CT**	6	0 (0)	6 (100)			6	1 (16.7)	5 (83.3)		
	**C**	17	3 (17.6)	14 (82.4)	-	0.508*	15	4 (26.7)	11 (73.3)	-	0.110*
	**T**	9	1(11.1)	8 (88.9)	-	0.591*	8	3 (37.5)	5 (62.5)	-	1*
rs2230724	**GG**	6	2 (33.3)	4 (66.7)			6	2 (33.3)	4 (66.7)		
	**AA**	7	2 (28.6)	5 (71.4)	-	0.393*	6	2 (33.3)	4 (66.7)	-	1*
	**AG**	7	0 (0)	7 (100)			5	2 (40)	3 (60)		
	**A**	14	2 (14.3)	12 (85.7)	-	0.549*	11	4 (36.4)	7 (63.6)	-	1*
	**G**	13	2 (15.4)	11 (84.6)	-	0.586*	11	4 (36.4)	7 (63.6)		1*
JAK3											
rs3212780	**GG**	9	2 (22.2)	7 (77.8)			8	4 (50)	4 (50)		
	**AA**	2	0 (0)	2 (100)	-	1*	2	0 (0)	2 (100)	-	0.493*
	**AG**	9	2 (22.2)	7 (77.8)			7	2 (28.6)	5 (71.4)		
	**A**	11	2 (18.2)	9 (81.8)	-	1*	9	2 (22.2)	7 (77.8)	-	0.334*
	**G**	18	4 (22.2)	14 (77.8)	-	1*	15	6 (40)	9 (60)	-	0.514*
rs3008	**GG**	6	0 (0)	6 (100)			5	0 (0)	5 (100)		
	**AA**	2	0 (0)	2 (100)	-	0.359*	1	0 (0)	1 (100)	-	0.102*
	**AG**	12	4 (33.3)	8 (66.7)			11	6 (54.5)	5 (45.5)		
	**A**	14	4 (28.6)	10 (71.4)	-	0.267	12	6 (50)	6 (50)	-	0.102*
	**G**	18	4 (22.2)	14 (77.8)	-	1*	16	6 (37.5)	10 (62.5)	-	1*
rs3212752	**TT**	19	4 (21.1)	15 (78.9)			-	-	-		
	**CC**	0	-	-	-	1*	-	-	-	-	-
	**CT**	1	0 (0)	1 (100)			-	-	-		
	**C**	1	0 (0)	1 (100)	-	1*	-	-	-	-	-
	**T**	-	-	-	-	-	-	-	-	-	-
OR: odds ratio; CI: confidence interval; NA: not available (indicates non-estimable values due to sparse data or quasi-complete separation); *: p value for Fisher's Exact Test											
