# Supplementary material for: Clinical and Pharmacogenetic Factors Associated with Response to JAK Inhibitors in Patients with Rheumatoid Arthritis: A Real-World Study of JAK1, JAK2, and JAK3 Gene Variants
Source: Pharmaceutics. 2026 Jul 11;18(7):846. doi: 10.3390/pharmaceutics18070846 (PMC13415438; doi:10.3390/pharmaceutics18070846)
Supplement: Supplementary file 1 [file pharmaceutics-18-00846-s001.zip › Table S48-S53. Predictors of EULAR response LDA and remission at 3 and 6 months in RA patients treated with upadacitinib (Bivariate analyisis).pdf]

Table S48. Upadacitinib EULAR response bivariate demographic and clinical analyses												
	3 months						6 months					
Clinical variables	N	EULAR response		OR	CI95%	p-value	N	EULAR response		OR	CI95%	p-value
		Satisfactory	Unsatisfactory					Satisfactory	Unsatisfactory			
**Sex**												
**Woman**	27	8 (29.6)	19 (70.4)	-	-	0.667	23	10 (43.5)	13 (56.5)	-	-	0.432
**Men**	9	2 (22.2)	7 (77.8)				8	2 (25.0)	6 (75.0)			
**Smoking**												
Smoker	8	3 (37.5)	5 (62.5)	-	-	0.665	6	3 (50.0)	3 (50.0)	-	-	0.665
Exsmoker	6	2 (33.3)	4 (66.7)				6	3 (50.0)	3 (50.0)			
No smoker	22	5 (22.7)	17 (77.3)				19	6 (31.6)	13 (68.4)			
**Age at DX**	36	46±12.2	38.8±13.6	-	-	0.145	31	44.1±11.9	38.1±14.1	-	-	0.218
**Years with RA**	36	9(6.3-11.5)	14.5 (7-23.8)	0.91	0.81-1.00	0.029	31	9.5 (5.8-13.5)	15 (7-21.5)	-	-	0.155
**Years from Dx till JAK inhibitor treatment**	36	4.0 (4.0-9.8)	10.5 (3.0-17.0)	0.91	0.81-1.00	0.057	31	6.5 (4.0-11.3)	9.0 (3.0-17.0)	-	-	0.367
**JAK inhibitor start age**	36	55.5 (43.7-62.0)	53.5 (45.3-60.0)	-	-	0.617	31	53.6±12.2	51.0±11.9	-	-	0.217
**Treatment duration with JAK inhibitor (months)**	36	24.8±9.6	19.8±15.4	-	-	0.263	31	22.4±7.7	25.4±15.3	-	-	0.473
**Biologic-naïve**												
**yes**	3	1 (33.3)	2 (66.7)	-	-	1	3	2 (66.7)	1 (33.3)	-	-	0.543
**No**	33	9 (27.3)	24 (72.7)				28	10 (35.7)	18 (64.3)			
**Number of previous BTs**	36	2.0 (1.0-3.0)	3.0 (1.3-3.8)	-	-	0.172	31	2.0 (0.8-3.3)	3.0 (1.5-3.5)	-	-	0.311
**Previous BTs duration (months)**	36	67.7 (29.3-123.4)	69.4 (19.0-140.1)	-	-	0.641	31	73.4 (37.6-124.04)	56.9 (14.7-124.4)	-	-	0.737
**BTs cause of suspensión**												
**Primary failure**	9	1 (11.1)	8 (88.9)	-	-	0.163	6	1 (16.7)	5 (83.3)	1	-	0.046
**Secondary failure**	18	7 (38.9)	11 (61.1)				16	9 (56.2)	7 (43.8)	6.42	0.79-138.7	
**Adverse events**	5	0 (0.0)	5 (100.0)				5	0 (0.0)	5 (100.0)	0	-	
**Toxicity**	0	-	-				0	-	-	-	-	
**Others**	0	-	-				0	-	-	-	-	
**Baseline RF (Cualtiative)**												
**Pos**	28	6 (21.4)	22 (78.6)	-	-	0.179	23	8 (34.8)	15 (65.2)	-	-	0.675
**Neg**	8	4 (50.0)	4 (50.0)				8	4 (50.0)	4 (50.0)			
**Baseline ACPA**												
**Pos**	31	8 (25.8)	23 (74.2)	-	-	0.603	26	9 (34.6)	17 (65.4)	-	-	0.349
**Neg**	5	2 (40.0)	3 (60.0)				5	3 (60.0)	2 (40.0)			
**Baseline CCI**												
**Absence**	20	6 (30.0)	14 (70.0)	-	-	0.672	17	7 (41.2)	10 (58.8)	-	-	0.773
**Low**	11	2 (18.2)	9 (81.8)				10	3 (30.0)	7 (70.0)			
**High**	5	2 (40.0)	3 (60.0)				4	2 (50.0)	2 (50.0)			
**BMI**	36	29.3±4.5	27.2±5.1	-	-	0.238	31	27.1±5.0	28.7±5.4	-	-	0.396
**JAK inhibitor dose change**												
**yes**	2	0 (0.0)	2 (100.0)	-	-	0.366	2	0 (0.0)	2 (100.0)	-	-	0.509
**no**	34	10 (29.4)	24 (70.6)				29	12 (41.4)	17 (58.6)			
**JAK inhibitor suspensión**												
**yes**	16	2 (12.5)	14 (87.5)	1	-	0.067	11	1 (9.1)	10 (90.9)	1	-	0.012
**No**	20	8 (40.0)	12 (60.0)	4.66	0.95-35.08		20	11 (55.0)	9 (45.0)	12.22	1.82-246.83	
**JAK inhibitor cause of suspension**												
**Primary failure**	4	0 (0.0)	4 (100.0)	-	-	1	1	0 (0.0)	1 (100.0)	-	-	0.454
**Secondary failure**	11	2 (18.2)	9 (81.8)				9	1 (11.1)	8 (88.9)			
**Adverse events**	2	0 (0.0)	2 (100.0)				2	1 (50.0)	1 (50.0)			
**Others**	0	-	-				0	-	-			
**BT after JAK inhibitor treatment**												
**yes**	17	1 (5.9)	16 (94.1)	1	-	0.005	12	2 (16.7)	10 (83.3)	1	-	0.045
**No**	19	9 (47.4)	10 (52.6)	14.4	2.22-286.96		19	10 (52.6)	9 (47.4)	5.55	1.08-42.93	
**Adverse events to JAK inhibitors**												
**yes**	15	3 (20.0)	12 (80.0)	-	-	0.378	13	4 (30.8)	9 (69.2)	-	-	0.440
**No**	21	7 (33.3)	14 (66.7)				18	8 (44.4)	10 (55.6)			
**Concomitant DMARDs**												
**MTX**	7	1 (14.3)	6 (85.7)	-	-	0.746	6	2 (33.3)	4 (66.7)	-	-	0.637
**HXQ**	1	0 (0.0)	1 (100.0)				1	1 (100.0)	0 (0.0)			
**SSZ**	0	0 (0.0)	0 (0.0)				0	-	-			
**LFN**	5	1 (20.0)	4 (80.0)				3	1 (33.3)	2 (66.7)			
**None**	23	8 (34.8)	15 (65.2)				21	8 (38.1)	13 (61.9)			
**Concomitant statins**												
**Yes**	11	3 (27.3)	8 (72.7)	-	-	1	9	3 (33.3)	6 (66.7)	-	-	1
**No**	25	7 (28.0)	18 (72.0)				22	9 (40.9)	13 (59.1)			
**Concomitant GC**												
**Yes**	22	4 (18.2)	18 (81.8)	-	-	0.140	17	3 (17.6)	14 (82.4)	1	-	0.007
**No**	14	6 (42.9)	8 (57.1)				14	9 (64.3)	5 (35.7)	8.40	1.75-51.54	
**Concomitant Vitamin D**												
**Yes**	13	2 (15.4)	11 (84.6)	-	-	0.269	11	2 (18.2)	9 (81.8)	1	-	0.081
**No**	23	8 (34.8)	15 (65.2)				20	10 (50.0)	10 (50.0)	4.50	0.87-34.70	
**Baseline DAS28**	36	4.4±1.3	4.9±1.2	-	-	0.399	31	4.3±1.4	5.1±1.2	-	-	0.140
**Baseline TJC**	36	6.0 (4.0-6.8)	6.0 (2.5-11.3)	-	-	0.093	31	6.0 (2.8-6.5)	7.0 (4.0-11.5)	0.88	0.73-1.02	0.082
**Baseline SJC**	36	3.0 (2.0-3.8)	4.0 (1.0-6.0)	-	-	0.141	31	2.5 (1.0-4.0)	5.0 (2.0-7.0)	0.78	0.56-0.99	0.042
**Baseline PVAS**	36	6.0 (4.3-6.8)	7.0 (5.0-8.0)	-	-	0.359	31	6.0 (4.5-7.0)	7.0 (5.0-8.0)	-	-	0.312
**Baseline MVAS**	36	5.0 (3.5-5.0)	6.0 (5.0-7.8)	-	-	0.137	31	5.0 (4.3-5.5)	6.0 (5.0-8.0)	-	-	0.118
**Baseline CRP**	36	4.0 (2.9-8.8)	7.8 (3.2-24.2)	-	-	0.323	31	4.0 (2.5-9.2)	8.0 (2.5-29.2)	-	-	0.230
**Baseline ESR**	36	16.5 (11.8-22.3)	26.0 (9.5-31.8)	-	-	0.373	31	16.5 (11.0-21.2)	27.0 (7.5-37.5)	0.96	0.89-1.01	0.072
**Baseline RF (Quantitative)**	36	26.5 (6.8-159.8)	73.5 (37.5-157.5)	-	-	0.976	31	51.5 (10.0-196.7)	57.0 (26.5-150.0)	-	-	0.472
**Baseline TC**	36	200.2±38.3	204.5±45.2	-	-	0.774	31	213.5±36.5	197.5±48.6	-	-	0.307
**Baseline LDL**	36	122.0±40.1	124.1±32.5	-	-	0.879	31	132.1±31	120.8±38.5	-	-	0.378
**Baseline TG**	36	78.0 (69.0-94.8)	97.5 (77.5-133.8)	0.98	0.95-1.00	0.078	31	85.0 (71.8-106.0)	96.0 (75.0-132.0)	-	-	0.324

EULAR: European League Against Rheumatism; TJC: tender joints count; SJC: swallowed joint count; PVAS: patient visual analogue scale; MVAS: physician visual analogue scale; RF: rheumatoid factor; ACPA: anti-citrullinated protein antibodies; ESR: erythrocyte sedimentation rate; CRP: C-reactive protein; TC: total cholesterol; LDL: low-density lipoprotein; TG: triglycerides; BMI: body mass index; CCI: Charlson Comorbidity Index; JAK inhibitor: Janus kinase inhibitor; BTs: biologic therapies; GC: glucocorticoids; DMARDs: disease-modifying antirheumatic drugs; MTX: methotrexate; HXQ:, hydroxychloroquine; LFN, leflunomide; SSZ: sulfasalazine; OR, odds ratio; CI, confidence interval; NA: not available (indicates non-estimable values due to sparse data or quasi-complete separation); *: p value for Fisher's Exact Test

Table S49. Upadacitinib EULAR response bivariate genetic analyses											
		3 months					6 months				
SNPs	Genotipe	N	EULAR response		OR CI95%	p-value	N	EULAR response		OR CI95%	p-value
			Satisfactory	Unsatisfactory				Satisfactory	Unsatisfactory		
JAK1											
**rs2230587**	**GG**	27	6 (22.2)	21 (77.8)	-	0.163	24	8 (33.3)	16 (66.7)	-	0.361
	**AA**	1	1 (100.0)	0 (0.0)			1	1 (100.0)	0 (0.0)		
	**AG**	8	3 (37.5)	5 (62.5)			6	3 (50.0)	3 (50.0)		
	**A**	9	4 (44.4)	5 (55.6)	-	0.226	7	4 (57.1)	3 (42.9)	-	0.383
	**G**	35	9 (25.7)	26 (74.3)	-	0.278	30	11 (36.7)	19 (63.3)	-	0.387
**rs310241**	**GG**	3	0 (0.0)	3 (100.0)	-	0.740	0	-	-	-	0.697
	**AA**	21	6 (28.6)	15 (71.4)			21	9 (42.9)	12 (57.1)		
	**AG**	12	4 (33.3)	8 (66.7)			10	3 (30.0)	7 (70.0)		
	**A**	33	10 (30.3)	23 (69.7)	-	0.544	31	-	-	-	-
	**G**	15	4 (26.7)	11 (73.3)	-	0.899	10	3 (30.0)	7 (70.0)	-	0.697

rs2230588	CC	3	0 (0.0)	3 (100.0)			0	-	-		
	TT	24	8 (33.3)	16 (66.7)	-	0.605	24	10 (41.7)	14 (58.3)	-	0.675
	CT	9	2 (22.2)	7 (77.8)			7	2 (28.6)	5 (71.4)		
	T	33	10 (30.3)	23 (69.7)	-	0.544	31	-	-	-	-
	C	12	2 (16.7)	10 (83.3)	-	0.438	7	2 (28.6)	5 (71.4)	-	0.675
rs10889504	GG	28	7 (25.0)	21 (75.0)			25	9 (36.0)	16 (64.0)		
	CC	2	1 (50.0)	1 (50.0)	-	0.506	1	1 (100.0)	0 (0.0)	-	0.610
	GC	6	2 (33.3)	4 (66.7)			5	2 (40.0)	3 (60.0)		
	C	8	3 (37.5)	5 (62.5)	-	0.658	6	3 (50.0)	3 (50.0)	-	0.652
	G	34	9 (26.5)	25 (73.5)	-	0.484	30	11 (36.7)	19 (63.3)	-	0.387
rs2780815	GG	7	2 (28.6)	5 (71.4)			2	2 (100.0)	0 (0.0)		
	TT	14	3 (21.4)	11 (78.6)	-	0.887	14	6 (42.9)	8 (57.1)	-	0.144
	GT	15	5 (33.3)	10 (66.7)			15	4 (26.7)	11 (73.3)		
	T	29	8 (27.6)	21 (72.4)	-	1	29	10 (34.5)	19 (65.5)	-	0.141
	G	22	7 (31.8)	15 (68.2)	-	0.706	17	6 (35.3)	11 (64.7)	-	0.667
JAK2											
rs10119004	GG	10	3 (30.0)	7 (70.0)			8	2 (25.0)	6 (75.0)		
	AA	10	1 (10.0)	9 (90.0)	-	0.401	7	2 (28.6)	5 (71.4)	-	0.438
	AG	16	6 (37.5)	10 (62.5)			16	8 (50.0)	8 (50.0)		
	A	26	7 (26.9)	19 (73.1)	-	1	23	10 (43.5)	13 (56.5)	-	0.432
	G	26	9 (34.6)	17 (65.4)	-	0.223	24	10 (41.7)	14 (58.3)	-	0.675
rs7857730	GG	9	2 (22.2)	7 (77.8)			8	2 (25.0)	6 (75.0)		
	TT	12	3 (25.0)	9 (75.0)	-	0.805	9	2 (22.2)	7 (77.8)	-	0.209
	GT	15	5 (33.3)	10 (66.7)			14	8 (57.1)	6 (42.9)		
	G	24	7 (29.2)	17 (70.8)	-	1	22	10 (45.5)	12 (54.5)	-	0.418
	T	27	8 (29.6)	19 (70.4)	-	1	23	10 (43.5)	13 (56.5)	-	0.432
rs2274472	CC	5	0 (0.0)	5 (100.0)	NA		4	0 (0.0)	4 (100.0)		
	TT	9	5 (55.6)	4 (44.4)	0.23 (0.04-1.19)	0.059	9	5 (55.6)	4 (44.4)	-	0.165
	CT	22	5 (22.7)	17 (77.3)	-		18	7 (38.9)	11 (61.1)		
	C	27	5 (18.5)	22 (81.5)	0.18 (0.03-0.91)	0.032	22	7 (31.8)	15 (68.2)	-	0.253
	T	31	10 (32.3)	21 (67.7)	-	0.293	27	12 (44.4)	15 (55.6)	-	0.138
rs2230722	CC	20	3 (15.0)	17 (85.0)			18	3 (16.7)	15 (83.3)	1	
	TT	2	1 (50.0)	1 (50.0)	-	0.147	2	1 (50.0)	1 (50.0)	5.00 (0.17-154.47)	0.005
	CT	14	6 (42.9)	8 (57.1)			11	8 (72.7)	3 (27.3)	13.33 (2.44-99.01)	
	C	34	9 (26.5)	25 (73.5)	-	0.484	29	11 (37.9)	18 (62.1)	-	1
	T	16	7 (43.8)	9 (56.2)	4.40 (0.97-24.59)	0.056	13	9 (69.2)	4 (30.8)	11.25 (2.26-73.48)	0.003
rs2230724	GG	11	3 (27.3)	8 (72.7)			9	2 (22.2)	7 (77.8)		
	AA	10	1 (10.0)	9 (90.0)	-	0.328	7	2 (28.6)	5 (71.4)	-	0.281
	AG	15	6 (40.0)	9 (60.0)			15	8 (53.3)	7 (46.7)		
	A	25	7 (28.0)	18 (72.0)	-	1	22	10 (45.5)	12 (54.5)	-	0.418
	G	26	9 (34.6)	17 (65.4)	-	0.223	24	10 (41.7)	14 (58.3)	-	0.675
JAK3											
rs3212780	GG	19	4 (21.1)	15 (78.9)			16	6 (37.5)	10 (62.5)		
	AA	0	-	-	-	0.341	0	-	-	-	0.886
	AG	17	6 (35.3)	11 (64.7)			15	6 (40.0)	9 (60.0)		
	A	17	6 (35.3)	11 (64.7)	-	0.341	15	6 (40.0)	9 (60.0)	-	0.886
	G	36	-	-	-	-	31	-	-	-	-
rs3008	GG	8	2 (25.0)	6 (75.0)			8	2 (25.0)	6 (75.0)		
	AA	11	3 (27.3)	8 (72.7)	-	1	9	3 (33.3)	6 (66.7)	-	0.578
	AG	17	5 (29.4)	12 (70.6)			14	7 (50.0)	7 (50.0)		
	A	28	8 (28.6)	20 (71.4)	-	1	23	10 (43.5)	13 (56.5)	-	0.432
	G	25	7 (28.0)	18 (72.0)	-	1	22	9 (40.9)	13 (59.1)	-	0.694
rs3212752	TT	32	9 (28.1)	23 (71.9)			27	10 (37.0)	17 (63.0)		
	CC	0	-	-	-	1	0	-	-	-	0.630
	CT	4	1 (25.0)	3 (75.0)			4	2 (50.0)	2 (50.0)		
	C	4	1 (25.0)	3 (75.0)	-	1	4	2 (50.0)	2 (50.0)	-	0.630
	T	36	-	-	-	-	31	-	-	-	-

OR: odds ratio; CI: confidence interval; NA: not available (indicates non-estimable values due to sparse data or quasi-complete separation); *: p value for Fisher's Exact Test

Table S50. Upadacitinib LDA bivariate demographic and clinical analyses

Clinical variables	3 months						6 months					
	N	LDA		OR	CI95%	p-value	N	LDA		OR	CI95%	p-value
		LDA	No LDA					LDA	No LDA			
**Sex**												
**Women**	27	4 (14.8)	23 (85.2)	-	-	0.553*	23	2 (8.7)	21 (91.3)	-	-	1*
**Men**	9	0 (0.0)	9 (100.0)				8	0 (0.0)	8 (100.0)			
**Smoking**												
Smoker	8	1 (12.5)	7 (87.5)				6	1 (16.7)	5 (83.3)			
Exsmoker	6	1 (16.7)	5 (83.3)	-	-	0.790*	6	0 (0.0)	6 (100.0)	-	-	0.632*
No smoker	22	2 (9.1)	20 (90.9)				19	1 (5.3)	18 (94.7)			
**Age at DX**	36	41.7±13.0	40.7±13.7	-	-	0.889	31	41.5±7.7	40.3±13.8	-	-	0.871
**Years with RA**	36	8.0 (6.8-9.8)	13.0 (7.0-20.8)	0.89	0.69-1.03	0.009	31	8.0 (7.5-8.5)	12 (7.0-20.0)	0.88	0.58-1.07	0.007
**Years from Dx till JAK inhibitor treatment**	36	4.0 (3.8-5.5)	9.5 (3.0-17.0)	0.88	0.67-1.04	0.028	31	3.5 (3.3-3.8)	9.0 (3.0-15.0)	0.80	0.41-1.06	0.001
**JAK inhibitor start age**	36	46.0 (39.8-54.5)	54.5(45.7-61.3)	-	-	0.454	31	46.00±8.48	52.44±12.13	-	-	0.465
**Treatment duration with JAK inhibitor (months)**	36	24.91±8.84	20.80±14.68	-	-	0.456	31	28.10±9.89	24.01±13.11	-	-	0.663
**Biologic-naïve**												
**Yes**	3	1 (33.3)	2 (66.7)	-	-	0.305*	3	1 (33.3)	2 (66.7)	-	-	0.187*
**No**	33	3 (9.1)	30 (90.9)				28	1 (3.6)	27 (96.4)			
**Number of previous BTs**	36	1.0 (0.8-1.8)	3.0 (1.0-3.3)	-	-	0.232	31	2 (1-3)	3 (1-8)	-	-	0.829
**Previous BTs duration (months)**	36	62.6 (43.9-93.5)	72.8(29.2-136)	-	-	0.643	31	62.6 (62.6-62.6)	68.7 (26.2-124.4)	-	-	0.783
**BTs cause of suspension**												
**Primary failure**	9	0 (0.0)	9 (100.0)				6	0 (0.0)	6 (100.0)			
**Secondary failure**	18	3 (16.7)	15 (83.3)				16	1 (6.2)	15 (93.8)			
**Adverse events**	5	0 (0.0)	5 (100.0)	-	-	0.722*	5	0 (0.0)	5 (100.0)	-	-	1*
**Toxicity**	0	-	-				0	-	-			
**Others**	0	-	-				0	-	-			
**Baseline RF (Cualtiative)**												
**Pos**	28	1 (3.6)	27 (96.4)	1	-	0.027*	23	0 (0.0)	23 (100.0)	1	-	0.060*
**Neg**	8	3 (37.5)	5 (62.5)	16.20	1.70-368.08		8	2 (25.0)	6 (75.0)	NA	NA-NA	
**Baseline ACPA**												
**Pos**	31	3 (9.7)	28 (90.3)	-	-	0.465*	26	2 (7.7)	24 (92.3)	-	-	1*
**Neg**	5	1 (20.0)	4 (80.0)				5	0 (0.0)	5 (100.0)			
**Baseline CCI**												
**Absence**	20	3 (15.0)	17 (85.0)				17	2 (11.8)	15 (88.2)			
**Low**	11	1 (9.1)	10 (90.9)	-	-	1*	10	0 (0.0)	10 (100.0)	-	-	0.634*
**High**	5	0 (0.0)	5 (100.0)				4	0 (0.0)	4 (100.0)			
**BMI**	36	26.1±5.0	28.1±5.1	-	-	0.506	31	30.2±1.3	27.9±5.3	-	-	0.172
**JAK inhibitor dose change**												
**Yes**	2	0 (0.0)	2 (100.0)	-	-	1*	2	0 (0.0)	2 (100.0)	-	-	1*
**No**	34	4 (11.8)	30 (88.2)				29	2 (6.9)	27 (93.1)			
**JAK inhibitor suspensión**												
**Yes**	16	2 (12.5)	14 (87.5)	-	-	1*	11	2 (18.2)	9 (81.8)	-	-	0.118*
**No**	20	2 (10.0)	18 (90.0)				20	0 (0.0)	20 (100.0)			
**JAK inhibitor cause of suspension**												
**Primary failure**	4	0 (0.0)	4 (100.0)	-	-	1*	1	0 (0.0)	1 (100.0)	-	-	1*
**Secondary failure**	11	2 (18.2)	9 (81.8)				9	2 (22.2)	7 (77.8)			

**Adverse events**	2	0 (0.0)	2 (100.0)				2	0 (0.0)	2 (100.0)			
**Others**	0	-	-				0	-	-			
**BT after JAK inhibitor treatment**												
**Yes**	17	1 (5.9)	16 (94.1)	-	-	0.605*	12	1 (8.3)	11 (91.7)	-	-	1*
**No**	19	3 (15.8)	16 (84.2)				19	1 (5.3)	18 (94.7)			
**Adverse events to JAK inhibitors**												
**Yes**	15	1 (6.7)	14 (93.3)	-	-	0.625*	13	0 (0.0)	13 (100.0)	-	-	0.496*
**No**	21	3 (14.3)	18 (85.7)				18	2 (11.1)	16 (88.9)			
**Concomitant DMARDs.**												
**MTX**	7	0 (0.0)	7 (100.0)	-	-	0.789*	6	0 (0.0)	6 (100.0)	-	-	0.277*
**HXQ**	1	0 (0.0)	1 (100.0)				1	0 (0.0)	1 (100.0)			
**SSZ**	0	-	-				0	-	-			
**LFN**	5	1 (20.0)	4 (80.0)				3	1 (33.3)	2 (66.7)			
**Ninguno**	23	3 (13.0)	20 (87.0)				21	1 (4.8)	20 (95.2)			
**Concomitant statins**												
**Yes**	11	0 (0.0)	11 (100.0)	-	-	0.290*	9	0 (0.0)	9 (100.0)	-	-	1*
**No**	25	4 (16.0)	21 (84.0)				22	2 (9.1)	20 (90.9)			
**Concomitant GC**												
**Yes**	22	1 (4.5)	21 (95.5)	-	-	0.277*	17	1 (5.9)	16 (94.1)	-	-	1*
**No**	14	3 (21.4)	11 (78.6)				14	1 (7.1)	13 (92.9)			
**Concomitant vitamin D**												
**Yes**	13	1 (7.7)	12 (92.3)	-	-	1*	11	1 (9.1)	10 (90.9)	-	-	1*
**No**	23	3 (13.0)	20 (87.0)				20	1 (5.0)	19 (95.0)			
**Baseline DAS28**	36	4.5±0.2	4.8±1.3	-	-	0.310	31	4.3±0.1	4.8±1.3	0.76	0.25-2.36	0.074
**Baseline TJC**	36	6.0 (5.5-6.5)	6.0 (2.8-9.0)	-	-	0.376	31	5.0 (4.5-5.5)	6.0 (3.0-9.0)	-	-	0.156
**Baseline SJC**	36	2.5 (1.8-3.8)	3.5 (1.0-6.0)	-	-	0.398	31	1.5 (1.3-1.8)	3.0 (1.0-6.0)	0.67	0.21-1.14	0.012
**Baseline PVAS**	36	6.0 (6.0-6.8)	6.5 (4.8-8.0)	-	-	0.352	31	7.5 (6.8-8.2)	6.0 (5.0-8.0)	-	-	0.478
**Baseline MVAS**	36	5.0 (5.0-5.0)	6.0 (3.0-7.0)	-	-	0.347	31	5.0 (5.0-5.0)	6.0 (5.0-7.0)	-	-	0.279
**Baseline CRP**	36	3.2 (2.4-4.0)	7.8 (3.6-22.6)	0.82	0.53-0.98	0.013	31	4.0 (4.0-4.0)	5.8 (2.4-18.4)	0.91	0.55-1.01	0.030
**Baseline ESR**	36	18.7±3.7	23.0±15.4	-	-	0.214	31	18.5 (16.3-20.8)	20.0 (9.0-31.0)	-	-	0.510
**Baseline RF (Quantitative)**	36	7.2 (5.9-10.8)	76.5 (33.5-190.5)	0.89	0.69-0.97	0.001	31	6.3 (5.4-7.1)	57.5 (23.0-190.0)	0.67	0.20-0.90	0.001
**Baseline TC**	36	172.5±35.0	207.2±42.7	-	-	0.139	31	163.0±52.3	206.5±43.4	-	-	0.442
**Baseline LDL**	36	95.7±46.5	127.1±31.6	-	-	0.273	31	101.5±75.6	126.8±33.4	-	-	0.717
**Baseline TG**	36	68.0 (56.8-87.5)	96.5 (75.5-130.8)	-	-	0.314	31	107.0 (87.5-126.5)	89.0 (74.0-129.0)	-	-	0.923

LDA: Low Disease activity; TJC: tender joints count; SJC: swallowed joint count; PVAS: patient visual analogue scale; MVAS: physician visual analogue scale; RF: rheumatoid factor; ACPA: anti-citrullinated protein antibodies; ESR: erythrocyte sedimentation rate; CRP: C-reactive protein; TC: total cholesterol; LDL: low-density lipoprotein; TG: triglycerides; BMI: body mass index; CCI: Charlson Comorbidity Index; JAK inhibitor: Janus kinase inhibitor; BTs: biologic therapies; GC: glucocorticoids; DMARDs: disease-modifying antirheumatic drugs; MTX: methotrexate; HXQ:, hydroxychloroquine; LFN, leflunomide; SSZ: sulfasalazine; OR, odds ratio; CI, confidence interval; NA: not available (indicates non-estimable values due to sparse data or quasi-complete separation); *: p value for Fisher's Exact Test

Table S51. Upadacitinib LDA bivariate genetic analyses

Gen / SNPs	Genotipe	3 months					6 months				
		N	LDA		OR CI95%	p-value	N	LDA		OR CI95%	p-value
			LDA	LDA				LDA	LDA		
JAK1											
**rs2230587**	**GG**	27	2 (7.4)	25 (92.6)	-	0.304*	24	1 (4.2)	23 (95.8)	-	0.406*
	**AA**	1	0 (0.0)	1 (100.0)			1	0 (0.0)	1 (100.0)		
	**AG**	8	2 (25.0)	6 (75.0)			6	1 (16.7)	5 (83.3)		
	**A**	9	2 (22.2)	7 (77.8)	-	0.255*	7	1 (14.3)	6 (85.7)	-	0.406*
	**G**	35	4 (11.4)	31 (88.6)	-	1*	30	2 (6.7)	28 (93.3)	-	1*
**rs310241**	**GG**	3	0 (0.0)	3 (100.0)	-	0.729*	0	-	-	-	0.034
	**AA**	21	2 (9.5)	19 (90.5)			21	0 (0.0)	21 (100.0)	1	
	**AG**	12	2 (16.7)	10 (83.3)			10	2 (20.0)	8 (80.0)	NA	
	**A**	33	4 (12.1)	29 (87.9)	-	1*	31	-	-	-	-
	**G**	15	2 (13.3)	13 (86.7)	-	1*	10	2 (20.0)	8 (80.0)	NA	0.034
**rs2230588**	**CC**	3	0 (0.0)	3 (100.0)	-	0.690*	0	-	-	-	1*
	**TT**	24	4 (16.7)	20 (83.3)			24	2 (8.3)	22 (91.7)		
	**CT**	9	0 (0.0)	9 (100.0)			7	0 (0.0)	7 (100.0)		
	**T**	33	4 (12.1)	29 (87.9)	-	1*	31	-	-	-	-
	**C**	12	0 (0.0)	12 (100.0)	-	0.278*	7	0 (0.0)	7 (100.0)	-	1*
**rs10889504**	**GG**	28	3 (10.7)	25 (89.3)	-	0.652*	25	2 (8.0)	23 (92.0)	-	1*
	**CC**	2	0 (0.0)	2 (100.0)			1	0 (0.0)	1 (100.0)		
	**GC**	6	1 (16.7)	5 (83.3)			5	0 (0.0)	5 (100.0)		
	**C**	8	1 (12.5)	7 (87.5)	-	1*	6	0 (0.0)	6 (100.0)	-	1*
	**G**	34	4 (11.8)	30 (88.2)	-	1*	30	2 (6.7)	28 (93.3)	-	1*
**rs2780815**	**GG**	7	0 (0.0)	7 (100.0)	-	0.500*	2	0 (0.0)	2 (100.0)	-	0.548*
	**TT**	14	1 (7.1)	13 (92.9)			14	0 (0.0)	14 (100.0)		
	**GT**	15	3 (20.0)	12 (80.0)			15	2 (13.3)	13 (86.7)		
	**T**	29	4 (13.8)	25 (86.2)	-	0.565*	29	2 (6.9)	27 (93.1)	-	1*
	**G**	22	3 (13.6)	19 (86.4)	-	1*	17	2 (11.8)	15 (88.2)	-	0.488*
JAK2											
**rs10119004**	**GG**	10	2 (20.0)	8 (80.0)	-	0.361*	8	1 (12.5)	7 (87.5)	-	1*
	**AA**	10	0 (0.0)	10 (100.0)			7	0 (0.0)	7 (100.0)		
	**AG**	16	2 (12.5)	14 (87.5)			16	1 (6.2)	15 (93.8)		
	**A**	26	2 (7.7)	24 (92.3)	-	0.304*	23	1 (4.3)	22 (95.7)	-	0.455*
	**G**	26	4 (15.4)	22 (84.6)	-	0.558*	24	2 (8.3)	22 (91.7)	-	1*
**rs7857730**	**GG**	9	2 (22.2)	7 (77.8)	-	0.538*	8	1 (12.5)	7 (87.5)	-	0.292*
	**TT**	12	1 (8.3)	11 (91.7)			9	1 (11.1)	8 (88.9)		
	**GT**	15	1 (6.7)	14 (93.3)			14	0 (0.0)	14 (100.0)		
	**G**	24	3 (12.5)	21 (87.5)	-	1*	22	1 (4.5)	21 (95.5)	-	0.503*
	**T**	27	2 (7.4)	25 (92.6)	-	0.255*	23	1 (4.3)	22 (95.7)	-	0.455*
**rs2274472**	**CC**	5	0 (0.0)	5 (100.0)	-	1*	4	0 (0.0)	4 (100.0)	-	0.651*
	**TT**	9	1 (11.1)	8 (88.9)			9	0 (0.0)	9 (100.0)		
	**CT**	22	3 (13.6)	19 (86.4)			18	2 (11.1)	16 (88.9)		
	**C**	27	3 (11.1)	24 (88.9)	-	1*	22	2 (9.1)	20 (90.9)	-	1*
	**T**	31	4 (12.9)	27 (87.1)	-	1*	27	2 (7.4)	25 (92.6)	-	1*
**rs2230722**	**CC**	20	2 (10.0)	18 (90.0)	-	1*	18	1 (5.6)	17 (94.4)	-	1*
	**TT**	2	0 (0.0)	2 (100.0)			2	0 (0.0)	2 (100.0)		
	**CT**	14	2 (14.3)	12 (85.7)			11	1 (9.1)	10 (90.9)		
	**C**	34	4 (11.8)	30 (88.2)	-	1*	29	2 (6.9)	27 (93.1)	-	1*
	**T**	16	2 (12.5)	14 (87.5)	-	1*	13	1 (7.7)	12 (92.3)	-	1*
**rs2230724**	**GG**	11	3 (27.3)	8 (72.7)	-	0.197*	9	2 (22.2)	7 (77.8)	-	0.122*
	**AA**	10	0 (0.0)	10 (100.0)			7	0 (0.0)	7 (100.0)		
	**AG**	15	1 (6.7)	14 (93.3)			15	0 (0.0)	15 (100.0)		
	**A**	25	1 (4.0)	24 (96.0)	0.11 (0.01-1)	0.040	22	0 (0.0)	22 (100.0)	4×10^{-9} (NA-NA)	0.022
	**G**	26	4 (15.4)	22 (84.6)	-	0.558*	24	2 (8.3)	22 (91.7)	-	1*
JAK3											
**rs3212780**	**GG**	19	2 (10.5)	17 (89.5)	-	1*	16	0 (0.0)	16 (100.0)	-	0.225*
	**AA**	0	-	-			0	-	-		
	**AG**	17	2 (11.8)	15 (88.2)			15	2 (13.3)	13 (86.7)		
	**A**	17	2 (11.8)	15 (88.2)	-	1*	15	2 (13.3)	13 (86.7)	-	0.225*
	**G**	36	-	-	-	-	31	-	-	-	-
**rs3008**	**GG**	8	1 (12.5)	7 (87.5)	-	0.415*	8	1 (12.5)	7 (87.5)	-	0.729*
	**AA**	11	0 (0.0)	11 (100.0)			9	0 (0.0)	9 (100.0)		
	**AG**	17	3 (17.6)	14 (82.4)			14	1 (7.1)	13 (92.9)		
	**A**	28	3 (10.7)	25 (89.3)	-	1*	23	1 (4.3)	22 (95.7)	-	0.455*
	**G**	25	4 (16.0)	21 (84.0)	-	0.290*	22	2 (9.1)	20 (90.9)	-	1*
**rs3212752**	**TT**	32	3 (9.4)	29 (90.6)	-	0.389*	27	2 (7.4)	25 (92.6)	-	1*
	**CC**	0	-	-			0	-	-		
	**CT**	4	1 (25.0)	3 (75.0)			4	0 (0.0)	4 (100.0)		

	C	4	1 (25.0)	3 (75.0)	-	0.389*	4	0 (0.0)	4 (100.0)	-	1*
	T	36	-	-	-	-	31	-	-	-	-

LDA: low disease activity; OR: odds ratio; CI: confidence interval; NA: not available (indicates non-estimable values due to sparse data or quasi-complete separation); *: p value for Fisher's Exact Test

Table S52. Upadacitinib remission bivariate demographic and clinical analyses

Clinical variables	3 months						6 months					
	N	Remission		OR	CI95%	p-value	N	Remission		OR	CI95%	p-value
		Sí Remission	No Remission					Remission	No Remission			
**Sex**												
**Woman**	27	3 (11.1)	24 (88.9)	-	-	0.581	23	8 (34.8)	15 (65.2)	-	-	0.610
**Men**	9	2 (22.2)	7 (77.8)				8	2 (25.0)	6 (75.0)			
**Smoking**												
Smoker	8	2 (25.0)	6 (75.0)	-	-	0.532	6	2 (33.3)	4 (66.7)	-	-	1
Exsmoker	6	0 (0.0)	6 (100.0)				6	2 (33.3)	4 (66.7)			
No smoker	22	3 (13.6)	19 (86.4)				19	6 (31.6)	13 (68.4)			
**Age at DX**	36	45.8±10.4	40±13.8	-	-	0.315	31	43.±12.8	39.1±13.8	-	-	0.461
**Years with RA**	36	10.0 (9.0-18.0)	12.0 (7.0-21.5)	-	-	0.515	31	11.0 (6.7-16.5)	12.0 (7.0-20.0)	-	-	0.428
**Years from Dx till JAK inhibitor treatment**	36	9 (4-15)	9 (3-17)	-	-	0.730	31	9.5 (4.0-13.8)	6.0 (3.0-17.0)	-	-	0.818
**JAK inhibitor start age**	36	57 (54-62)	53 (45-60.5)	-	-	0.395	31	53.9±13.5	51.1±11.3	-	-	0.585
**Treatment duration with JAK inhibitor (months)**	36	21.6±8.9	21.2±14.9	-	-	0.925	31	22.1±8.3	25.3±14.5	-	-	0.436
**Biologic-naïve**												
**yes**	3	0 (0.0)	3 (100.0)	-	-	1	3	1 (33.3)	2 (66.7)	-	-	1
**No**	33	5 (15.2)	28 (84.8)				28	9 (32.1)	19 (67.9)			
**Number of previous BTs**	3	3 (3-3)	3 (1-3.5)	-	-	0.810	31	2 (1-3)	3 (1-4)	-	-	0.388
**Previous BTs duration (months)**	36	72.8 (29.4-123.1)	64.6 (22.8-130.2)	-	-	0.860	31	74.1 (29.4-124.4)	59.8 (16.1-121.6)	-	-	0.646
**BTs cause of suspensión**												
**Primary failure**	9	1 (11.1)	8 (88.9)	-	-	0.671	6	1 (16.7)	5 (83.3)	NA	NA-NA	0.088
**Secondary failure**	18	4 (22.2)	14 (77.8)				16	8 (50.0)	8 (50.0)	1	-	
**Adverse events**	5	0 (0.0)	5 (100.0)				5	0 (0.0)	5 (100.0)	NA	NA-NA	
**Toxicity**	0	-	-				0	-	-	-	-	
**Others**	0	-	-				0	-	-	-	-	
**Baseline RF (Cualtiative)**												
**Pos**	28	4 (14.3)	24 (85.7)	-	-	1	23	7 (30.4)	16 (69.6)	-	-	0.712
**Neg**	8	1 (12.5)	7 (87.5)				8	3 (37.5)	5 (62.5)			
**Baseline ACPA**												
**Pos**	31	4 (12.9)	27 (87.1)	-	-	0.549	26	7 (26.9)	19 (73.1)	-	-	0.295
**Neg**	5	1 (20.0)	4 (80.0)				5	3 (60.0)	2 (40.0)			
**Baseline CCI**												
**Absence**	20	3 (15.0)	17 (85.0)	0.26	0.03-2.63	0.098	17	5 (29.4)	12 (70.6)	-	-	0.748
**Low**	11	0 (0.0)	11 (100.0)	0.01	NA-NA		10	3 (30.0)	7 (70.0)			
**High**	5	2 (40.0)	3 (60.0)	1	-		4	2 (50.0)	2 (50.0)			
**BMI**	36	31.8±2.9	27.1±5.0	1.19	0.99-1.52	0.019	31	27.2±5.3	28.5±5.2	-	-	0.553
**JAK inhibitor dose change**												
**yes**	2	0 (0.0)	2 (100.0)	-	-	1	2	0 (0.0)	2 (100.0)	-	-	1
**no**	34	5 (14.7)	29 (85.3)				29	10 (34.5)	19 (65.5)			
**JAK inhibitor suspensión**												
**yes**	16	0 (0.0)	16 (100.0)	1	-	0.031	11	0 (0.0)	11 (100.0)	0.01	NA-NA	0.004
**No**	20	5 (25.0)	15 (75.0)	NA	NA-NA		20	10 (50.0)	10 (50.0)	1	-	
**JAK inhibitor cause of suspension**												
**Primary failure**	4	0 (0.0)	4 (100.0)	-	-	1	1	0 (0.0)	1 (100.0)	-	-	0.250
**Secondary failure**	11	0 (0.0)	11 (100.0)				9	0 (0.0)	9 (100.0)			
**Adverse events**	2	0 (0.0)	2 (100.0)				2	1 (50.0)	1 (50.0)			
**Others**	0	-	-				0	-	-			
**BT after JAK inhibitor treatment**												
**Yes**	17	0 (0.0)	17 (100.0)	1	-	0.047	12	1 (8.3)	11 (91.7)	1	-	0.023
**No**	19	5 (26.3)	14 (73.7)	NA	NA-NA		19	9 (47.4)	10 (52.6)	9.90	1.47-199.79	
**Adverse events to JAK inhibitors**												
**Yes**	15	1 (6.7)	14 (93.3)	-	-	0.376	13	3 (23.1)	10 (76.9)	-	-	0.352
**No**	21	4 (19.0)	17 (81.0)				18	7 (38.9)	11 (61.1)			
**Concomitant DMARDs.**												
**MTX**	7	1 (14.3)	6 (85.7)	-	-	1	6	2 (33.3)	4 (66.7)	-	-	0.726
**HXQ**	1	0 (0.0)	1 (100.0)				1	0 (0.0)	1 (100.0)			
**SSZ**	0	-	-				0	-	-			
**LFN**	5	0 (0.0)	5 (100.0)				3	0 (0.0)	3 (100.0)			
**Ninguno**	23	4 (17.4)	19 (82.6)				21	8 (38.1)	13 (61.9)			
**Concomitant statins**												
**Yes**	11	2 (18.2)	9 (81.8)	-	-	0.630	9	3 (33.3)	6 (66.7)	-	-	0.934
**No**	25	3 (12.0)	22 (88.0)				22	7 (31.8)	15 (68.2)			
**Concomitant GC**												
**Yes**	22	2 (9.1)	20 (90.9)	-	-	0.296	17	2 (11.8)	15 (88.2)	1	-	0.007
**No**	14	3 (21.4)	11 (78.6)				14	8 (57.1)	6 (42.9)	10.00	1.87-80.79	
**Concomitant vitamin D**												
**Yes**	13	1 (7.7)	12 (92.3)	-	-	0.418	11	1 (9.1)	10 (90.9)	1	-	0.040
**No**	23	4 (17.4)	19 (82.6)				20	9 (45.0)	11 (55.0)	8.18	1.21-165.04	
**Baseline DAS28**	36	4.3±2	4.8±1.1	-	-	0.585	31	4.1±1.4	5.09±1.16	-	-	0.108
**Baseline TJC**	36	4 (3-6)	6 (4-9)	0.85	0.61-1.05	0.081	31	5.0 (2.3-6.0)	7.0 (4.0-13.0)	0.82	0.63-0.98	0.017
**Baseline SJC**	36	3 (2-4)	3 (1-6)	-	-	0.229	31	2.5 (1.0-3.8)	4.0 (2.0-7.0)	0.80	0.57-1.02	0.055
**Baseline PVAS**	36	5 (3-7)	6 (5-8)	-	-	0.252	31	5.5 (3.5-6.0)	8.0 (5.0-8.0)	0.71	0.47-1.01	0.048
**Baseline MVAS**	36	5 (2-7)	5 (5-7)	-	-	0.434	31	5.0 (2.8-5.0)	6.0 (5.0-8.0)	0.73	0.49-1.03	0.072
**Baseline CRP**	36	5.0 (4.0-14.3)	6.1 (2.7-20.1)	-	-	0.976	31	3.5 (2.4-6.9)	8.0 (3.8-29.0)	-	-	0.272
**Baseline ESR**	36	13.6±9.9	23.9±14.8	0.93	0.82-1.01	0.083	31	16.5 (11.0-19.5)	27.0 (9.0-37.0)	0.94	0.88-1.01	0.040
**Baseline RF (Quantitative)**	36	69 (34-211)	61 (21-155)	-	-	0.535	31	51.5 (13.2-165.8)	57.0 (23.0-160.0)	-	-	0.841
**Baseline TC**	36	217±33	201.1±44.3	-	-	0.378	31	212.1±39	199.7±47.1	-	-	0.449
**Baseline LDL**	36	139.6±29.1	121±34.7	-	-	0.244	31	128.2±32.7	123.8±37.7	-	-	0.746
**Baseline TG**	36	88 (82-97)	96 (73-132)	-	-	0.437	31	78.0 (69.3-92.5)	98.0 (76.0-140.0)	0.97	0.93-0.99	0.016

TJC: tender joints count; SJC: swallowed joint count; PVAS: patient visual analogue scale; MVAS: physician visual analogue scale; RF: rheumatoid factor; ACPA: anti-citrullinated protein antibodies; ESR: erythrocyte sedimentation rate; CRP: C-reactive protein; TC: total cholesterol; LDL: low-density lipoprotein; TG: triglycerides; BMI: body mass index; CCI: Charlson Comorbidity Index; JAK inhibitor: Janus kinase inhibitor; BTs: biologic therapies; GC: glucocorticoids; DMARDs: disease-modifying antirheumatic drugs; MTX: methotrexate; HXQ:, hydroxychloroquine; LFN, leflunomide; SSZ: sulfasalazine; OR, odds ratio; CI, confidence interval; NA: not available (indicates non-estimable values due to sparse data or quasi-complete separation); *: p value for Fisher's Exact Test

Table S53. Upadacitinib remission bivariate genetic analyses

SNPs	Genotipe	3 months					6 months				
		N	Remission		OR CI95%	p-value	N	Remission		OR CI95%	p-value
			Remission	No Remission				Remission	No Remission		
**JAK1**											
**rs2230587**	GG	27	3 (11.1)	24 (88.9)	-	0.196	24	7 (29.2)	17 (70.8)	-	0.418
	AA	1	1 (100.0)	0 (0.0)			1	1 (100.0)	0 (0.0)		
	AG	8	1 (12.5)	7 (87.5)			6	2 (33.3)	4 (66.7)		
	A	9	2 (22.2)	7 (77.8)	-	0.581	7	3 (42.9)	4 (57.1)	-	0.651
	G	35	4 (11.4)	31 (88.6)	-	0.138	30	9 (30.0)	21 (70.0)	-	0.322
**rs310241**	GG	3	0 (0.0)	3 (100.0)	-	1	0	-	-	-	0.313

	AA	21	3 (14.3)	18 (85.7)			21	8 (38.1)	13 (61.9)		
	AG	12	2 (16.7)	10 (83.3)			10	2 (20.0)	8 (80..0)		
	A	33	5 (15.2)	28 (84.8)	-	1	31	-	-	-	-
	G	15	2 (13.3)	13 (86.7)	-	1	10	2 (20.0)	8 (80..0)	-	0.313
**rs2230588**	CC	3	0 (0.0)	3 (100.0)			0	-	-		
	TT	24	3 (12.5)	21 (87.5)	-	0.746	24	8 (33.3)	16 (66.7)	-	1
	CT	9	2 (22.2)	7 (77.8)			7	2 (28.6)	5 (71.4)		
	T	33	5 (15.2)	28 (84.8)	-	1	31	-	-	-	-
	C	12	2 (16.7)	10 (83.3)	-	1	7	2 (28.6)	5 (71.4)	-	1
**rs10889504**	GG	28	3 (10.7)	25 (89.3)			25	7 (28.0)	18 (72.0)		
	CC	2	1 (50.0)	1 (50.0)	-	0.174	1	1 (100.0)	0 (0.0)	-	0.403
	GC	6	1 (16.7)	5 (83.3)			5	2 (40.0)	3 (60.0)		
	C	8	2 (25.0)	6 (75.0)	-	0.304	6	3 (50.0)	3 (50.0)	-	0.357
	G	34	4 (11.8)	30 (88.2)	-	0.261	30	9 (30.0)	21 (70.0)	-	0.322
**rs2780815**	GG	7	2 (28.6)	5 (71.4)			2	2 (100.0)	0 (0.0)	1	
	TT	14	1 (7.1)	13 (92.9)	-	0.391	14	5 (35.7)	9 (64.3)	5.00 × 10^{-9} (NA-NA)	0.070
	GT	15	2 (13.3)	13 (86.7)			15	3 (20.0)	12 (80.0)	1.00 × 10^{-8} (NA-NA)	
	T	29	3 (10.3)	26 (89.7)	-	0.244	29	8 (27.6)	21 (72.4)	8.00 × 10^{-9} (NA-NA)	0.034
	G	22	4 (18.2)	18 (81.8)	-	0.628	17	5 (29.4)	12 (70.6)	-	0.708
**JAK2**											
**rs10119004**	GG	10	1 (10.0)	9 (90.0)			8	2 (25.0)	6 (75.0)		
	AA	10	1 (10.0)	9 (90.0)	-	1	7	2 (28.6)	5 (71.4)	-	0.803
	AG	16	3 (18.8)	13 (81.2)			16	6 (37.5)	10 (62.5)		
	A	26	4 (15.4)	22 (84.6)	-	1	23	8 (34.8)	15 (65.2)	-	0.610
	G	26	4 (15.4)	22 (84.6)	-	1	24	8 (33.3)	16 (66.7)	-	0.812
**rs7857730**	GG	9	0 (0.0)	9 (100.0)			8	1 (12.5)	7 (87.5)		
	TT	12	1 (8.3)	11 (91.7)	-	0.269	9	2 (22.2)	7 (77.8)	-	0.145
	GT	15	4 (26.7)	11 (73.3)			14	7 (50.0)	7 (50.0)		
	G	24	4 (16.7)	20 (83.3)	-	0.645	22	8 (36.4)	14 (63.6)	-	0.444
	T	27	5 (18.5)	22 (81.5)	-	0.301	23	9 (39.1)	14 (60.9)	-	0.165
**rs2274472**	CC	5	0 (0.0)	5 (100.0)	1		4	0 (0.0)	4 (100.0)		
	TT	9	4 (44.4)	5 (55.6)	NA	0.008	9	5 (55.6)	4 (44.4)	-	0.133
	CT	22	1 (4.5)	21 (95.5)	NA		18	5 (27.8)	13 (72.2)		
	C	27	1 (3.7)	26 (96.3)	0.05 (0.01-0.40)	0.009	22	5 (22.7)	17 (77.3)	0.23 (0.04-1.19)	0.075
	T	31	5 (16.1)	26 (83.9)	-	0.333	27	10 (37.0)	17 (63.0)	-	0.277
**rs2230722**	CC	20	1 (5.0)	19 (95.0)	1		18	3 (16.7)	15 (83.3)	1	
	TT	2	1 (50.0)	1 (50.0)	18.99 (0.51-976.46)	0.090	2	1 (50.0)	1 (50.0)	5.00 (1.15-38.34)	0.060
	CT	14	3 (21.4)	11 (78.6)	5.18 (0.58-112.12)		11	6 (54.5)	5 (45.5)	6.00 (0.17-154.47)	
	C	34	4 (11.8)	30 (88.2)	-	0.261	29	9 (31.0)	20 (69.0)	-	1
	T	16	4 (25.0)	12 (75.0)	-	0.149	13	7 (53.8)	6 (46.2)	5.83 (1.20-35.07)	0.028
**rs2230724**	GG	11	0 (0.0)	11 (100.0)			9	1 (11.1)	8 (88.9)		
	AA	10	1 (10.0)	9 (90.0)	-	0.153	7	2 (28.6)	5 (71.4)	-	0.225
	AG	15	4 (26.7)	11 (73.3)			15	7 (46.7)	8 (53.3)		
	A	25	5 (20.0)	20 (80.0)	-	0.295	22	9 (40.9)	13 (59.1)	-	0.107
	G	26	4 (15.4)	22 (84.6)	-	0.675	24	8 (33.3)	16 (66.7)	-	1
**JAK3**											
**rs3212780**	GG	19	1 (5.3)	18 (94.7)			16	6 (37.5)	10 (62.5)		
	AA	0	-	-	-	0.113	0	-	-	-	0.519
	AG	17	4 (23.5)	13 (76.5)			15	4 (26.7)	11 (73.3)		
	A	17	4 (23.5)	13 (76.5)	-	0.167	15	4 (26.7)	11 (73.3)	-	0.519
	G	36	-	-	-	-	31	-	-	-	-
**rs3008**	GG	8	1 (12.5)	7 (87.5)			8	2 (25.0)	6 (75.0)		
	AA	11	2 (18.2)	9 (81.8)	-	1	9	3 (33.3)	6 (66.7)	-	1
	AG	17	2 (11.8)	15 (88.2)			14	5 (35.7)	9 (64.3)		
	A	28	4 (14.3)	24 (85.7)	-	1	23	8 (34.8)	15 (65.2)	-	1
	G	25	3 (12.0)	22 (88.0)	-	0.630	22	7 (31.8)	15 (68.2)	-	1
**rs3212752**	TT	32	5 (15.6)	27 (84.4)			27	8 (29.6)	19 (70.4)		
	CC	0	-	-	-	1	0	-	-	-	0.577
	CT	4	0 (0.0)	4 (100.0)			4	2 (50.0)	2 (50.0)		
	C	4	0 (0.0)	4 (100.0)	-	1	4	2 (50.0)	2 (50.0)	-	0.577
	T	36	-	-	-	-	31	-	-	-	-

OR: odds ratio; CI: confidence interval; NA: not available (indicates non-estimable values due to sparse data or quasi-complete separation); *: p value for Fisher's Exact Test
