# Supplementary material for: Clinical and Pharmacogenetic Factors Associated with Response to JAK Inhibitors in Patients with Rheumatoid Arthritis: A Real-World Study of JAK1, JAK2, and JAK3 Gene Variants
Source: Pharmaceutics. 2026 Jul 11;18(7):846. doi: 10.3390/pharmaceutics18070846 (PMC13415438; doi:10.3390/pharmaceutics18070846)
Supplement: Supplementary file 1 [file pharmaceutics-18-00846-s001.zip › Table S5. Linkage desequilibrium.pdf]

Table S3. Linkage disequilibrium

Drug	CHR_A	BP_A	SNP_A	CHR_B	BP_B	SNP_B	R2	D′
**Tofacitinib**	1	64845579	rs2230587	1	64924820	rs10889504	0.651	1
		64837655	rs310241		64844806	rs2230588	0.785	0.881
	9	5071049	rs10119004	9	5084049	rs7857730	0.510	0.771
		5071049	rs10119004		4985542	rs2274472	0.225	0.517
		5071049	rs10119004		5050706	rs2230722	0.200	0.669
		5071049	rs10119004		5081780	rs2230724	0.639	0.823
		5084049	rs7857730		4985542	rs2274472	0.246	0.509
		5084049	rs7857730		5081780	rs2230724	0.796	1
		4985542	rs2274472		5081780	rs2230724	0.293	0.612
**Baricitinib**	1	64845579	rs2230587	1	64924820	rs10889504	0.690	0.903
		64837655	rs310241		64844806	rs2230588	0.953	1
		64837655	rs310241		64835928	rs2780815	0.356	1
		64844806	rs2230588		64835928	rs2780815	0.342	1
	9	5071049	rs10119004	9	5084049	rs7857730	0.527	0.818
		5071049	rs10119004		5081780	rs2230724	0.607	0.838
		5084049	rs7857730		4985542	rs2274472	0.210	0.485
		5084049	rs7857730		5081780	rs2230724	0.892	1
		4985542	rs2274472		5081780	rs2230724	0.288	0.592
**Filgotinib**	1	64845579	rs2230587	1	64924820	rs10889504	0.529	1
		64845579	rs2230587		64835928	rs2780815	0.214	1
		64837655	rs310241		64844806	rs2230588	0.727	0.839
		64837655	rs310241		64835928	rs2780815	0.411	1
	9	5071049	rs10119004	9	5084049	rs7857730	0.390	0.735
		5071049	rs10119004		5081780	rs2230724	0.456	0.757
		5084049	rs7857730		4985542	rs2274472	0.250	0.537
		5084049	rs7857730		5081780	rs2230724	0.926	1
		4985542	rs2274472		5081780	rs2230724	0.237	0.508
**Upadacitinib**	1	64845579	rs2230587	1	64924820	rs10889504	0.503	0.640
		64845579	rs2230587		64835928	rs2780815	0.266	1
		64837655	rs310241		64844806	rs2230588	0.823	1
		64837655	rs310241		64835928	rs2780815	0.530	1
		64844806	rs2230588		64835928	rs2780815	0.490	1
	9	5071049	rs10119004	9	5084049	rs7857730	0.616	0.870
		5071049	rs10119004		5081780	rs2230724	0.860	0.941
		5084049	rs7857730		5081780	rs2230724	0.751	1

CHR: chromosome; SNP: single nucleotide polymorphisms; BP: physical position in base pairs (bp) for each locus; D': linkage disequilibrium coefficient; r2 correlation coefficient of allele frequencies
