# Supplementary material for: Clinical and Pharmacogenetic Factors Associated with Response to JAK Inhibitors in Patients with Rheumatoid Arthritis: A Real-World Study of JAK1, JAK2, and JAK3 Gene Variants
Source: Pharmaceutics. 2026 Jul 11;18(7):846. doi: 10.3390/pharmaceutics18070846 (PMC13415438; doi:10.3390/pharmaceutics18070846)
Supplement: Supplementary file 1 [file pharmaceutics-18-00846-s001.zip › Table S6-S11. Haplotype frequency estimation EULAR repsonse, LDA, remission at 3 and 6 months Baricitinib.pdf]

Table S6. Haplotype frequency estimation EULAR response at 3 months of Baricitinib.

JAK1 SNPs					EULAR Response			
**rs2230587**	**rs310241**	**rs2230588**	**rs10889504**	**rs2780815**	**Total**	**Satisfactory**	**Unsatisfactory**	**Cumulative frequency**
G	A	T	G	T	0.454	0.500	0.423	0.454
G	G	C	G	G	0.261	0.166	0.326	0.715
A	A	T	C	G	0.136	0.250	0.057	0.852
G	A	T	G	G	0.090	0.055	0.115	0.943
A	A	T	G	G	0.034	NA	0.057	0.977
G	G	T	G	G	0.011	NA	0.019	0.988
G	A	T	C	G	0.011	0.027	NA	1
G	A	T	C	T	0	0	NA	1
***JAK2* SNPs**					**EULAR Response**			
**rs10119004**	**rs7857730**	**rs2274472**	**rs2230722**	**rs2230724**	**Total**	**Satisfactory**	**Unsatisfactory**	**Cumulative frequency**
A	T	T	C	A	0.287	0.406	0.288	0.287
G	G	C	C	G	0.190	0.163	0.201	0.477
A	T	T	T	A	0.135	0	0.162	0.612
G	G	T	C	G	0.134	0.066	0.119	0.746
G	T	T	C	A	0.050	0.103	NA	0.796
A	T	C	C	A	0.046	NA	0.063	0.843
A	G	C	C	G	0.036	0.037	0.038	0.880
G	T	C	C	G	0.034	NA	0.038	0.914
A	T	C	T	A	0.028	NA	0.042	0.942
G	T	T	T	A	0.026	0.073	NA	0.969
G	G	T	T	G	0.025	0.072	0.025	0.994
G	T	C	C	A	0.005	NA	0.019	1
A	G	T	C	G	0	NA	2e–04	1
G	T	T	C	G	0	0.027	NA	1
**JAK3 SNPs**					**EULAR Response**			
**rs3212780**	**rs3008**		**rs3212752**		**Total**	**Satisfactory**	**Unsatisfactory**	**Cumulative frequency**
G	G		T		0.296	0.287	0.283	0.296
G	A		T		0.291	0.351	0.287	0.587
A	A		T		0.262	0.176	0.264	0.850
A	G		T		0.070	0.101	0.087	0.920
G	A		C		0.032	NA	0.044	0.953
A	G		C		0.026	0	0.032	0.979
G	G		C		0.015	0.027	NA	0.995
A	A		C		0.004	0.055	0	1

SNP: single nucleotide polymorphism; JAK: Janus kinase; NA: not available (frequency could not be estimated due to low counts); Total: overall haplotype frequency in the study population. EULAR: European Alliance of Associations for Rheumatology; Satisfactory/Unsatisfactory: clinical response categories according to EULAR criteria.

Table S7. Haplotype frequency estimation LDA at 3 months of Baricitinib

JAK1 SNPs					LDA			
**rs2230587**	**rs310241**	**rs2230588**	**rs10889504**	**rs2780815**	**Total**	**LDA**	**No LDA**	**Cumulative frequency**
G	A	T	G	T	0.454	0.600	0.433	0.454
G	G	C	G	G	0.261	0.100	0.282	0.715
A	A	T	C	G	0.136	0.300	0.115	0.852
G	A	T	G	G	0.090	NA	0.102	0.943
A	A	T	G	G	0.034	NA	0.038	0.977
G	G	T	G	G	0.011	NA	0.012	0.988
G	A	T	C	G	0.011	NA	0.012	1
G	A	T	C	T	0	NA	0	1
***JAK2* SNPs**					**LDA**			
**rs10119004**	**rs7857730**	**rs2274472**	**rs2230722**	**rs2230724**	**Total**	**LDA**	**No LDA**	**Cumulative frequency**
A	T	T	C	A	0.287	0.500	0.284	0.287
G	G	C	C	G	0.190	0.100	0.180	0.477
A	T	T	T	A	0.135	NA	0.133	0.612
G	G	T	C	G	0.134	NA	0.144	0.746
G	T	T	C	A	0.050	0.100	0.047	0.796
A	T	C	C	A	0.046	NA	0.051	0.843
A	G	C	C	G	0.036	0	0.040	0.880
G	T	C	C	G	0.034	NA	0.038	0.914
A	T	C	T	A	0.028	NA	0.028	0.942
G	T	T	T	A	0.026	0.100	0.010	0.969
G	G	T	T	G	0.025	NA	0.032	0.994
G	T	C	C	A	0.005	NA	0.007	1

A	G	T	C	G	0	NA	0	1
G	T	T	C	G	0	NA	0	1
JAK3 SNPs					LDA			
rs3212780	rs3008		rs3212752		Total	LDA	No LDA	Cumulative frequency
G	G		T		0.296	0.570	0.261	0.296
G	A		T		0.291	0.129	0.315	0.587
A	A		T		0.262	0.070	0.284	0.850
A	G		T		0.070	0.129	0.062	0.920
G	A		C		0.032	NA	0.038	0.953
A	G		C		0.026	NA	0.035	0.979
G	G		C		0.015	0.100	NA	0.995
A	A		C		0.004	NA	0.002	1

SNP: single nucleotide polymorphism; JAK: Janus kinase; NA: not available (frequency could not be estimated due to low counts); Total: overall haplotype frequency in the study population; LDA: low disease activity

Table S8. Haplotype frequency estimation remission at 3 months of Baricitinib

JAK1 SNPs					Remission			
rs2230587	rs310241	rs2230588	rs10889504	rs2780815	Total	Remission	No Remission	Cumulative frequency
G	A	T	G	T	0.454	0.461	0.451	0.454
G	G	C	G	G	0.261	0.192	0.290	0.715
A	A	T	C	G	0.136	0.230	0.096	0.852
G	A	T	G	G	0.090	0.076	0.096	0.943
A	A	T	G	G	0.034	NA	0.048	0.977
G	G	T	G	G	0.011	NA	0.016	0.988
G	A	T	C	G	0.011	0.038	NA	1
G	A	T	C	T	0	0	NA	1
JAK2 SNPs					Remission			
rs10119004	rs7857730	rs2274472	rs2230722	rs2230724	Total	Remission	No Remission	Cumulative frequency
A	T	T	C	A	0.287	0.382	0.283	0.287
G	G	C	C	G	0.190	0.190	0.221	0.477
A	T	T	T	A	0.135	0	0.166	0.612
G	G	T	C	G	0.134	0.087	0.094	0.746
G	T	T	C	A	0.050	0.106	0.021	0.796
A	T	C	C	A	0.046	NA	0.060	0.843
A	G	C	C	G	0.036	0.040	0.025	0.880
G	T	C	C	G	0.034	0	0.032	0.914
A	T	C	T	A	0.028	NA	0.036	0.942
G	T	T	T	A	0.026	0.049	0.016	0.969
G	G	T	T	G	0.025	0.104	0.022	0.994
G	T	C	C	A	0.005	NA	0.011	1
A	G	T	C	G	0	NA	0.008	1
G	T	T	C	G	0	0	NA	1
JAK3 SNPs					Remission			
rs3212780	rs3008		rs3212752		Total	Remission	No Remission	Cumulative frequency
G	G		T		0.296	0.230	0.324	0.296
G	A		T		0.291	0.384	0.252	0.587
A	A		T		0.262	0.307	0.243	0.850
A	G		T		0.070	0	0.098	0.920
G	A		C		0.032	0	0.036	0.953
A	G		C		0.026	0.038	0.012	0.979
G	G		C		0.015	NA	0.031	0.995
A	A		C		0.004	0.038	0	1

SNP: single nucleotide polymorphism; JAK: Janus kinase; NA: not available (frequency could not be estimated due to low counts); Total: overall haplotype frequency in the study population.

Table S9. Haplotype frequency estimation EULAR response at 6 months of Baricitinib

JAK1 SNPs					EULAR Response			
rs2230587	rs310241	rs2230588	rs10889504	rs2780815	Total	Satisfactory	Unsatisfactory	Cumulative frequency
G	A	T	G	T	0.507	0.555	0.476	0.507
G	G	C	G	G	0.204	0.250	0.166	0.712
G	A	T	G	G	0.146	0.055	0.214	0.858
A	A	T	C	G	0.076	0.083	0.0714	0.935
G	A	C	G	T	0.018	0	0.023	0.953
G	G	T	G	G	0.013	0.027	NA	0.966
A	A	T	G	G	0.012	NA	0.023	0.979
A	G	T	C	G	0.012	NA	0.023	0.992
G	A	C	G	G	0.007	0.027	NA	1

G	G	T	G	T	0	0	NA	1
A	A	T	C	T	0	0	0	1
A	G	T	C	T	0	NA	0	1
JAK2 SNPs					EULAR Response			
**rs10119004**	**rs7857730**	**rs2274472**	**rs2230722**	**rs2230724**	**Total**	**Satisfactory**	**Unsatisfactory**	**Cumulative frequency**
G	G	C	C	G	0.314	0.237	0.378	0.314
A	T	T	C	A	0.179	0.239	0.116	0.494
A	T	T	T	A	0.128	0.165	0.084	0.622
G	G	T	C	G	0.093	0.090	0.101	0.716
A	G	C	C	G	0.057	0.086	0	0.774
G	T	T	C	A	0.040	0.031	0.043	0.814
G	T	C	C	G	0.039	0.027	0.056	0.854
A	T	C	C	A	0.038	0.064	0.030	0.89
G	G	T	T	G	0.030	0.029	0.031	0.923
A	T	C	T	A	0.020	NA	0.050	0.944
G	T	T	C	G	0.016	0.027	NA	0.960
A	G	T	C	G	0.016	0	0.060	0.976
G	T	C	C	A	0.015	NA	0.031	0.992
A	T	T	C	G	0.007	NA	0.014	1
G	T	C	T	G	0	NA	NA	1
A	T	C	C	G	0	NA	NA	1
A	G	C	T	G	0	NA	0	1
G	G	C	T	G	0	NA	NA	1
JAK3 SNPs					EULAR Response			
**rs3212780**	**rs3008**		**rs3212752**		**Total**	**Satisfactory**	**Unsatisfactory**	**Cumulative frequency**
G	A		T		0.370	0.397	0.355	0.370
G	G		T		0.309	0.241	0.382	0.679
A	G		T		0.203	0.258	0.141	0.883
A	A		T		0.065	0.047	0.073	0.948
G	G		C		0.038	0.055	0	0.987
A	A		C		0.012	NA	0.023	1
G	A		C		0	0	0	1
SNP: single nucleotide polymorphism; JAK: Janus kinase; NA: not available (frequency could not be estimated due to low counts); Total: overall haplotype frequency in the study population. EULAR: European Alliance of Associations for Rheumatology; Satisfactory/Unsatisfactory: clinical response categories according to EULAR criteria								
**Table S10. Haplotype frequency estimation LDA at 6 months of Baricitinib**								
JAK1 SNPs					LDA			
**rs2230587**	**rs310241**	**rs2230588**	**rs10889504**	**rs2780815**	**Total**	**LDA**	**No LDA**	**Cumulative frequency**
G	A	T	G	T	0.507	0	0.546	0.507
G	G	C	G	G	0.204	0	0.171	0.712
G	A	T	G	G	0.146	0.5	0.140	0.858
A	A	T	C	G	0.076	NA	0.093	0.935
G	A	C	G	T	0.018	0.071	NA	0.953
G	G	T	G	G	0.013	NA	0.015	0.966
A	A	T	G	G	0.012	NA	0.015	0.979
A	G	T	C	G	0.012	NA	0.015	0.992
G	A	C	G	G	0.007	0.071	NA	1
G	G	T	G	T	0	NA	0	1
A	A	T	C	T	0	NA	0	1
A	G	T	C	T	0	NA	0	1
JAK2 SNPs					LDA			
**rs10119004**	**rs7857730**	**rs2274472**	**rs2230722**	**rs2230724**	**Total**	**LDA**	**No LDA**	**Cumulative frequency**
G	G	C	C	G	0.314	0.428	0.293	0.314
A	T	T	C	A	0.179	0.214	0.177	0.494
A	T	T	T	A	0.128	0.142	0.123	0.622
G	G	T	C	G	0.093	NA	0.113	0.716
A	G	C	C	G	0.057	0	0.071	0.774
G	T	T	C	A	0.040	0.071	0.032	0.814
G	T	C	C	G	0.039	NA	0.045	0.854
A	T	C	C	A	0.038	0.071	0.027	0.893
G	G	T	T	G	0.030	NA	0.036	0.923
A	T	C	T	A	0.020	NA	0.027	0.944
G	T	T	C	G	0.016	NA	0.022	0.960
A	G	T	C	G	0.016	0.071	0	0.976
G	T	C	C	A	0.015	NA	0.018	0.992
A	T	T	C	G	0.007	NA	0.010	1

G	T	C	T	G	0	NA	NA	1
A	T	C	C	G	0	NA	NA	1
A	G	C	T	G	0	NA	NA	1
G	G	C	T	G	0	NA	NA	1
JAK3 SNPs					LDA			
rs3212780	rs3008		rs3212752		Total	LDA	No LDA	Cumulative frequency
G	A		T		0.370	0.428	0.369	0.370
G	G		T		0.309	0.142	0.333	0.679
A	G		T		0.203	0.428	0.166	0.883
A	A		T		0.065	0	0.067	0.948
G	G		C		0.038	NA	0.046	0.987
A	A		C		0.012	NA	0.015	1
G	A		C		0	NA	0	1
SNP: single nucleotide polymorphism; JAK: Janus kinase; NA: not available (frequency could not be estimated due to low counts); Total: overall haplotype frequency in the study population; LDA: low disease activity								

Table S11. Haplotype frequency estimation of remission at 6 months Baricitinib.

JAK1 SNPs					Remission			
rs2230587	rs310241	rs2230588	rs10889504	rs2780815	Total	Remission	No Remission	Cumulative frequency
G	A	T	G	T	0.507	0.590	0.467	0.507
G	G	C	G	G	0.204	0.227	0.196	0.712
G	A	T	G	G	0.146	NA	0.210	0.858
A	A	T	C	G	0.076	0.136	0.053	0.935
G	A	C	G	T	0.018	NA	0.032	0.953
G	G	T	G	G	0.013	0.045	NA	0.966
A	A	T	G	G	0.012	NA	0.017	0.979
A	G	T	C	G	0.012	NA	0.017	0.992
G	A	C	G	G	0.007	NA	0.003	1
G	G	T	G	T	0	NA	NA	1
A	A	T	C	T	0	NA	NA	1
A	G	T	C	T	0	NA	NA	1
JAK2 SNPs					Remission			
rs10119004	rs7857730	rs2274472	rs2230722	rs2230724	Total	Remission	No Remission	Cumulative frequency
G	G	C	C	G	0.314	0.084	0.421	0.314
A	T	T	C	A	0.179	0.357	0.118	0.494
A	T	T	T	A	0.128	0.181	0.080	0.622
G	G	T	C	G	0.093	0.045	0.099	0.716
A	G	C	C	G	0.057	0.097	0	0.774
G	T	T	C	A	0.040	0.052	0.033	0.814
G	T	C	C	G	0.039	0.045	0.041	0.854
A	T	C	C	A	0.038	0	0.045	0.89
G	G	T	T	G	0.030	0.045	0.023	0.923
A	T	C	T	A	0.020	0.045	0.039	0.944
G	T	T	C	G	0.016	0.045	NA	0.960
A	G	T	C	G	0.016	NA	0.062	0.976
G	T	C	C	A	0.015	NA	0.023	0.992
A	T	T	C	G	0.007	NA	0.011	1
G	T	C	T	G	0	NA	NA	1
A	T	C	C	G	0	NA	NA	1
A	G	C	T	G	0	NA	0	1
G	G	C	T	G	0	NA	NA	1
JAK3 SNPs					Remission			
rs3212780	rs3008		rs3212752		Total	Remission	No Remission	Cumulative frequency
G	A		T		0.370	0.454	0.330	0.370
G	G		T		0.309	0.272	0.347	0.679
A	G		T		0.203	0.181	0.187	0.883
A	A		T		0.065	NA	0.097	0.948
G	G		C		0.038	0.090	0	0.987
A	A		C		0.012	NA	0.017	1
G	A		C		0	0	0	1
SNP: single nucleotide polymorphism; JAK: Janus kinase; NA: not available (frequency could not be estimated due to low counts); Total: overall haplotype frequency in the study population.								
